# Ischaemic preconditioning regulates cardiac transcriptome via DNA methylation conferring cardio-protection from ischaemic reperfusion injury

**DOI:** 10.1093/ehjopen/oeaf124

**Published:** 2025-10-10

**Authors:** Abdul Waheed Khan, Shafaat Hussain, Ahmed Elmahdy, Yalda Kakaei, Aaron Shekka Espinosa, Abhishek Jha, Elmir Omerovic, Misbah Aziz, Scott Maxwell, Karin A M Jandeleit-Dahm, Bjorn Redfors

**Affiliations:** Department of Diabetes, School of Translational Medicine, Monash University, Level 5, 99 Commercial Road, Melbourne 3004, Australia; Department of Molecular and Clinical Medicine, Institute of Medicine, Gothenburg University, PO Box 100, 405 30 Gothenburg, Sweden; Wallenberg Centre for Molecular and Translational Medicine, Institute of Medicine, University of Gothenburg, Blå stråket 5 b wallenberglab/su 41345, Gothenburg, Sweden; Department of Molecular and Clinical Medicine, Institute of Medicine, Gothenburg University, PO Box 100, 405 30 Gothenburg, Sweden; Wallenberg Centre for Molecular and Translational Medicine, Institute of Medicine, University of Gothenburg, Blå stråket 5 b wallenberglab/su 41345, Gothenburg, Sweden; Department of Molecular and Clinical Medicine, Institute of Medicine, Gothenburg University, PO Box 100, 405 30 Gothenburg, Sweden; Wallenberg Centre for Molecular and Translational Medicine, Institute of Medicine, University of Gothenburg, Blå stråket 5 b wallenberglab/su 41345, Gothenburg, Sweden; Department of Molecular and Clinical Medicine, Institute of Medicine, Gothenburg University, PO Box 100, 405 30 Gothenburg, Sweden; Wallenberg Centre for Molecular and Translational Medicine, Institute of Medicine, University of Gothenburg, Blå stråket 5 b wallenberglab/su 41345, Gothenburg, Sweden; Department of Molecular and Clinical Medicine, Institute of Medicine, Gothenburg University, PO Box 100, 405 30 Gothenburg, Sweden; Wallenberg Centre for Molecular and Translational Medicine, Institute of Medicine, University of Gothenburg, Blå stråket 5 b wallenberglab/su 41345, Gothenburg, Sweden; Department of Molecular and Clinical Medicine, Institute of Medicine, Gothenburg University, PO Box 100, 405 30 Gothenburg, Sweden; Department of Cardiology, Sahlgrenska University Hospital, 405 83 Gothenburg, Sweden; Department of Diabetes, School of Translational Medicine, Monash University, Level 5, 99 Commercial Road, Melbourne 3004, Australia; Department of Diabetes, School of Translational Medicine, Monash University, Level 5, 99 Commercial Road, Melbourne 3004, Australia; Department of Diabetes, School of Translational Medicine, Monash University, Level 5, 99 Commercial Road, Melbourne 3004, Australia; Leibniz Institute for Diabetes Research, Heinrich Heine University Dusseldorf, Auf'm Hennekamp 65, 40225 Düsseldorf, Germany; Department of Molecular and Clinical Medicine, Institute of Medicine, Gothenburg University, PO Box 100, 405 30 Gothenburg, Sweden; Wallenberg Centre for Molecular and Translational Medicine, Institute of Medicine, University of Gothenburg, Blå stråket 5 b wallenberglab/su 41345, Gothenburg, Sweden; Department of Cardiology, Sahlgrenska University Hospital, 405 83 Gothenburg, Sweden

**Keywords:** Myocardial ischaemic preconditioning: Myocardial stunning, Myocardial infarction, Cardiac transcriptomics, DNA methylation, Ischaemia-reperfusion injury

## Abstract

**Aims:**

Myocardial ischaemic preconditioning (IPC) increases myocardial ability to withstand ischaemic injury. Myocardial stunning is a reversible dysfunction, while necrosis results in irreversible cell death. The link between IPC, stunning, and necrosis remains unclear. This study aimed to utilize a novel 13.5-min ischaemia-reperfusion (I/R) rat model, distinct from conventional I/R models, to identify transcriptomic changes associated with IPC and investigate the role of DNA methylation in regulating these changes, particularly in relation to myocardial stunning and necrosis.

**Methods and results:**

A novel rat model of cardiac I/R injury was used, with IPC induced by two 5-min ischaemia-reperfusion cycles followed by 13.5-min of ischaemia, and a control group undergoing 13.5-min of ischaemia without IPC. Myocardial samples were collected at early (T1) and 4-h (T2) post-reperfusion, representing stunned myocardium in the IPC group and necrosis in the control group. RNA sequencing, DNA methyltransferase (DNMT) activity assay, Chromatin immunoprecipitation (ChIP), and DNA methylation analyses were performed. IPC reprogrammed the cardiac transcriptome, with 53 genes differentially expressed at T1 and 166 at T2, including key regulators of inflammation (Nfkbia), DNA repair (Gadd45b, Parp14), and stress responses (Cebpd, Jun). IPC reduced global DNMT activity, promoting hypomethylation of protective genes like *Cebpd*, *Nfkbia*, *Gadd45b*, *Jun*, and *Aplod1* at T1, while selectively hypermethylating maladaptive genes like *Tmem200c* and *Fgfr4*. ChIP assays revealed reduced Dnmt1 binding at *Jun* and *Parp14* promoters, aligning with increased protein levels.

**Conclusion:**

IPC re-programmes the cardiac transcriptome through dynamic DNA methylation, enhancing myocardial resilience while increasing stunning as an adaptive mechanism to limit necrosis.

## Graphical Abstract

**Graphical Abstract:**
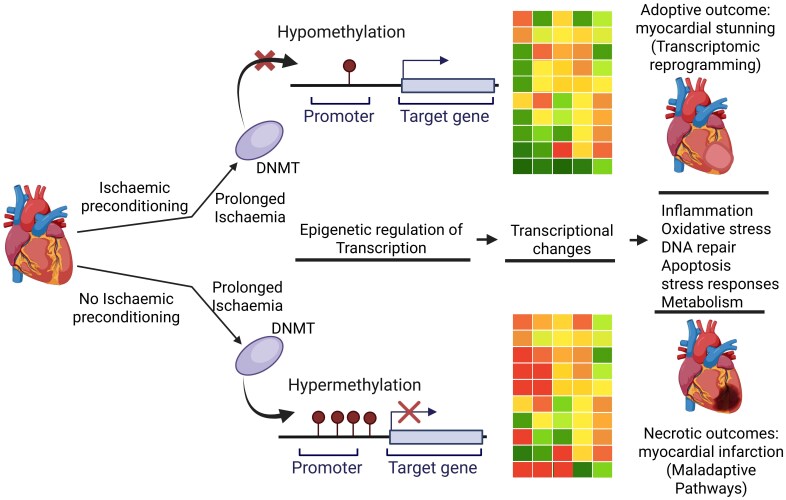

Translational perspective Myocardial ischaemic preconditioning (IPC) reduces myocardial infarction size while intensifying reversible myocardial stunning, highlighting its dual effects on ischaemia-reperfusion injury. Our study reveals that IPC exerts these effects through dynamic reprogramming of the cardiac transcriptome and epigenome. IPC reduces DNA methyltransferase activity, leading to selective hypomethylation of protective genes (Cebpd, Nfkbia, Gadd45b, Jun, and Aplod1) and hypermethylation of maladaptive genes (Tmem200c, Fgfr4, and Stk32c). These epigenetic changes regulate key pathways related to inflammation, oxidative stress, DNA repair, and cellular adaptation, providing a mechanistic basis for IPC’s cardioprotective effects while explaining its distinct impacts on myocardial necrosis and stunning. Traditionally viewed as detrimental, myocardial stunning might serve as an adaptive mechanism to limit necrosis.

The reversible nature of both myocardial stunning and epigenetic changes identified in this study suggests exciting therapeutic opportunities to modulate injury outcomes. Strategies that replicate IPC-induced epigenetic reprogramming could shift the balance from irreversible necrosis to reversible stunning, thereby improving myocardial recovery. Mechanistic studies are essential to further unravel the pathways mediating these epigenetic modifications, their phase-specific effects, and their potential reversibility. Progressively, these insights could inform the development of precision therapies targeting epigenetic regulators, with the ultimate goal of translating these findings into clinical applications for ischaemic heart disease.

## Introduction

The interplay between ischaemic preconditioning (IPC), myocardial stunning, and myocardial necrosis represents a complex spectrum of cardiac responses to ischaemic stress, each with distinct pathophysiological and molecular underpinnings.^1^ Ischaemic preconditioning, a phenomenon where brief episodes of ischaemia confer protection against subsequent prolonged ischaemic insults, offers a paradoxical yet potentially therapeutic insight into cardiac resilience.^2^ In contrast, myocardial stunning, characterized by reversible post-ischaemic contractile dysfunction in the absence of irreversible damage,^3^ and myocardial necrosis, the irreversible loss of cardiac myocytes due to prolonged ischaemia, depict the spectrum of injury and adaptation in ischaemic heart disease.^4^

Our previous work has suggested a connection between IPC and myocardial stunning, demonstrating that IPC intensifies stunning while reducing infarct size following ischaemia-reperfusion (I/R).^5^ Specifically, we found that when rats were subjected to 13.5 min of ischaemia-reperfusion preceded by two 5-min cycles of IPC, myocardial stunning occurred at 4 h post-reperfusion but resolved within 48 h. In contrast, rats subjected to 13.5 min of ischaemia without IPC developed irreversible myocardial necrosis at 4 h post-reperfusion.^5^ This raises an important question: how does IPC provide protection against necrosis while intensifying myocardial stunning, a phenomenon traditionally regarded as detrimental?

Epigenetics mechanisms including DNA methylation are emerging as key players in regulating gene expression changes, influencing genes involved in inflammation, oxidative stress, apoptosis, and DNA repair in the settings of I/R injury.^6^ However, the specific role of epigenetic mechanisms in the relationship between IPC, myocardial stunning, and myocardial necrosis remains poorly understood.

This study aims to utilize our novel rat model of I/R injury to profile the transcriptomic and DNA methylation changes associated with IPC. By comparing the early changes immediately following IPC and the later phases of reperfusion, where stunning or necrosis develop, we aim to identify the molecular alterations induced by IPC that protect against myocardial necrosis while promoting myocardial stunning. Additionally, we seek to distinguish IPC-induced myocardial stunning from irreversible necrosis, providing insights into IPC's protective mechanisms.

## Methods

### Animals

**Figure 1:**
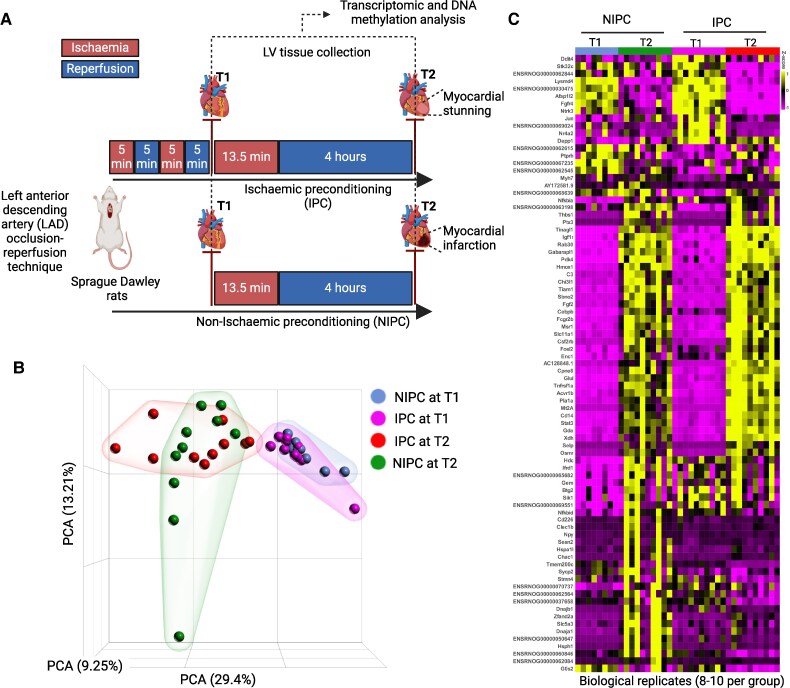
Transcriptomic profiling of myocardial ischaemic preconditioning. (*A*) Schematic diagram of the experimental design for RNA-seq in a novel rat model of ischaemia-reperfusion injury for profiling transcriptomic changes induced by ischaemic preconditioning at an early time point (T1) and at time point (T2) 4 h post prolonged ischaemia. (*B*) Principal component analysis plot for RNA sequencing data for all four groups of rats including non-ischaemic preconditioning at T1, ischaemic preconditioning at T1, ischaemic preconditioning at T2 and non-ischaemic preconditioning at T2 (*n* = 8–10). (*C*) The heatmap showing top 25 differentially expressed genes fold change (FC) < −2, >2, false discovery rate step up <0.05) in multiple comparison analysis among all experimental groups (*n* = 8–10). Figures generated with Biorender.

This study involved 40 male Sprague-Dawley rats, each weighing between 300–350 grams and aged 6–8 weeks. These rats were obtained from Janvier Labs (Le Genest-Saint-Isle, France) and underwent a one-week acclimatization period at the Laboratory of Experimental Biomedicine, Gothenburg, Sweden, before any surgical interventions. During both the acclimatization and the experimental stages, the rats were kept under strict environmental controls, including a steady temperature of 21°C and a 12-h day/night cycle, with free access to a standard lab diet and water. The experimental procedures adhered to the ARRIVE guidelines in compliance with Swedish regulations and received the approval of the North Stockholm Animal Ethics Committee (Dnr 5.8.18-11014/2023). The experimental design is outlined in *Figure 1A*.

### Study design

Ischaemia-reperfusion injury with or without IPC was induced via temporary left anterior descending artery ligation (LAD) as previously described.^5^ Animals were initially sedated with ketamine and xylazine, followed by continuous intravenous infusion for anaesthesia throughout the experiment. Mechanical ventilation with supplemental oxygen ensured consistent physiological conditions. The rats were systematically categorized into two distinct groups to explore the differential impacts of IPC and direct ischaemic exposure on myocardial tissue.

1. Ischaemic preconditioning (*n* = 20) group of rats underwent two cycles of ischaemia-reperfusion (5 min each) before a sustained 13.5-min ischaemia. Successful occlusion was confirmed by observing cardiac akinesia, left ventricular blanching, and electrocardiogram changes. Reperfusion was visually confirmed after removing the suture. Tissue samples from ten rats were collected immediately after preconditioning (T1) and from another ten rats 4 h post-surgery (T2), representing stunned myocardium.
2. Non-ischaemic preconditioning (*n* = 20) group of rats served as the control, experiencing only the sustained 13.5-min ischaemic insult without preconditioning cycles. Tissue samples from ten rats were collected immediately after the chest incision (T1) and from another ten rats 4 h after surgery (T2), representing infarcted myocardium.

All collected tissue samples were immediately frozen in liquid nitrogen for subsequent ribonucleic acid (RNA) sequencing aiming to profile gene expression changes that occur under different ischaemic conditions.

### Total RNA extraction

Left ventricle tissue samples were homogenized in liquid nitrogen. Total RNA was then extracted from homogenized tissue samples of all experimental groups using TRIzol reagent® (Invitrogen Australia, Mt Waverely, Vic, Australia) and Direct-zol^TM^ RNA miniprep kit (Zymo Research; Irvine CA, USA), following the manufacturer’s instructions, as described previously.^7^ Isolated RNA was either used for RNA sequencing or reverse transcription polymerase chain reaction (RT-PCR).

### RNA sequencing

NEBNext® Ribo-Zero Magnetic kit (ribosomal RNA removal) module (New England Biolabs, Ipswich, MA, USA) was used to enrich messenger RNA (mRNA) from 1μg of total RNA. Barcoded libraries were generated using the NEBNext® Ultra™ Directional RNA Library Prep Kit for Illumina® (New England Biolabs) following the manufacturer’s instructions. Deep sequencing was performed using Illumina NovaSeq PE 150 (San Diego, CA) at the Novogene Company Limited (Hong Kong). Sequence reads underwent quality trimming with Skewer.^8^ Trimmed reads were mapped to the rat genome using STAR aligner.^9^ Tags aligning to genes were counted using FeatureCounts with Ensembl annotations (rn7-Rat Ensembl 108). Two control samples with higher ribosomal RNA percentage (4.31% and 17.73%) were excluded from further analysis. Interpretive analysis was performed using Partek Flow software. Normalisation and differential gene expression was determined using the DESeq2 method within the Partek Flow. Genes with less than 10 reads average across all samples were excluded from the analysis. The false discovery rate (FDR) threshold was set to less than 0.05 and fold change (FC) of at least 2 (+2, −2) was considered as significant. Heatmaps, volcano plots and box and whiskers dot plots for RNA-seq data were generated in the Partek flow software.

### Quantitative PCR

Total RNA was reverse transcribed to complementary DNA (cDNA) using the high-capacity cDNA conversion kit (Applied Biosystems; Foster City, CA, USA). Levels of transcripts were quantified by RT-PCR with the FastStart Universal SYBR Green Master Mix (Roche; Melbourne Australia) on an ABI 7900HT PCR cycler (Applied Biosystems). Transcript levels of gene of interest were normalized to the expression of the housekeeping gene *18S*. The relative quantification was calculated using the ΔCT formula. Primers used in RT-PCR are listed in Supplementary material online, *Table S1*.

### DNA methyltransferase activity

DNA methyltransferase (DNMT) activity was measured using the ab11347—DNMT activity quantification kit (colorimetric) by Abcam (UK) blindly. In this DNMT activity assay, a universal DNMT substrate is coated onto microplate wells. DNA methyltransferase enzymes transfer methyl group to cytosine from Adomet to methylate DNA substrate and then this methylated DNA can be recognized with an anti-5-methylcytosine antibody. The amount of methylated DNA can then be measured through reading the absorbance in a spectrophotometer at a wavelength of 450 nm. DNA methyltransferase activity assay was performed using 10μg nuclear extracts from all cardiac tissues which were prepared using nuclear extraction kit (ab113474, Abcam). DNA methyltransferase activity was calculated using the following formula: DNMT activity (opticat density (OD)/h/μg) = sample OD − Blank OD/(Protein amount (μg)×hours)×1000.

### Gene promoter DNA methylation analysis

DNA methylation analysis was performed using a MethylMiner Enrichment Kit (Invitrogen, CA, USA). Genomic DNA was purified from LV cardiac tissue using DNeasy Blood and Tissue Kit (Qiagen, Germany). Purified DNA (1 µg) was used to assess DNA methylation of gene promoters as described previously.^10^ Briefly, methylated cytosines were captured with MethylMiner Enrichment Kit (Invitrogen, CA, USA) and level of methylation was assessed with promoter-specific primers coupled with ABI 7900HT RT-qPCR system and fluorescence-based FastStart Universal SYBR Green technology (Roche, Basel, Switzerland). Methylated and non-methylated control duplexes provided by manufacturer were used as controls for methyl-CpG-binding-domain (MBD) capture. The amount of DNA pulled down by MBD protein was normalized to input (starting DNA material) of each sample. The primers used for detection of CpG islands in the gene promoter are indicated in Supplementary material online in supplementary material online, *Table S2*.

### Chromatin immunoprecipitation

Chromatin immunoprecipitation (ChIP) was performed as described previously.^11^ Briefly, cells were crosslinked with 1% formaldehyde, lysed, and chromatin was sheared by sonication. Immunoprecipitation of soluble chromatin was carried out using antibodies targeting rat Dnmt1 (AB188453, Abcam, Cambridge, UK) overnight at 4°C. DNA-protein complexes were captured with dynabeads coated with protein A (Invitrogen), washed, and eluted. Crosslinks were reversed, and DNA was purified for downstream analysis by qPCR.

### Enzyme-linked immunosorbent assay

Quantification of Jun and Parp14 in cardiac tissues was performed using commercially available enzyme-linked immunosorbent assay (ELISA) kits (For rat Jun: Antibodies, A80322; For rat Parp14: MyBioSource, MBS9939587) according to the manufacturer’s instructions. Briefly, 96-well plates pre-coated with capture antibody were incubated with standards and samples for 2 h at 37°C. After washing, a biotinylated detection antibody was added, followed by incubation with streptavidin-HRP conjugate. Colour development was achieved using tetramethylbenzidine substrate, and the reaction was stopped with stop solution. Absorbance was measured at 450 nm using a microplate reader. Concentrations were calculated from a standard curve generated using known concentrations of the target analyte.

### Statistical analysis

Data are presented as mean ± standard error of the mean (SEM). The statistical analysis for RT-PCR and DNMT assay data was performed using GraphPad Prism Software (version 9.0.1). The unpaired two-tailed *t*-test for parametric data was used to determine significance between two groups. A *P*-value <0.05 was considered statistically significant.

## Results

### Transcriptome profiling of myocardial ischaemic preconditioning

We utilised our novel rat model of I/R injury where acute myocardial ischaemia was induced in 6- to 8-week-old male Sprague-Dawley rats by an open-chest LAD artery occlusion-reperfusion technique.^5^ The animals were randomized into either an IPC or a non-ischaemic preconditioning (NIPC) group, and IPC was induced by two short ischaemia-reperfusion episodes followed by a prolonged ischaemic exposure (*Figure 1A*). Cardiac tissue from the affected myocardium were harvested either at early time point (T1) or after a 4 h time point (T2) from the IPC and NIPC groups of animals for RNA sequencing and DNA methylation analysis (*Figure 1A*). In our previous study,^5^ we assessed cardiac function following thoracotomy using echocardiography at 4, 24, and 48 h of reperfusion. With IPC, which involved two cycles of 5 min of ischaemia/reperfusion followed by 13.5 min of ischaemia, echocardiography revealed marked myocardial stunning at 4 h of reperfusion, which resolved completely by 48 h of reperfusion. In contrast, without IPC, myocardial necrosis was evident at 4 h of reperfusion, with sustained injury persisting at 48 h, as assessed by 23,5-triphenyltetrazolium (TTC) staining and echocardiography.^5^ Principal component analysis plot was used to visualize the sample variation between NIPC and IPC group of animals at T1 and T2. The results showed a clear clustering of the biological replicates of NIPC and IPC group of animals at T1 and T2 (*Figure 1B*). These analyses indicate that all the variability due to biological replicates or sequencing depth have been normalized and clear clustering of all four sample groups were visible making it ready for further analysis including differential gene expression analysis and pathway enrichment analysis.

### Differential gene expression and pathway analysis

**Figure 2:**
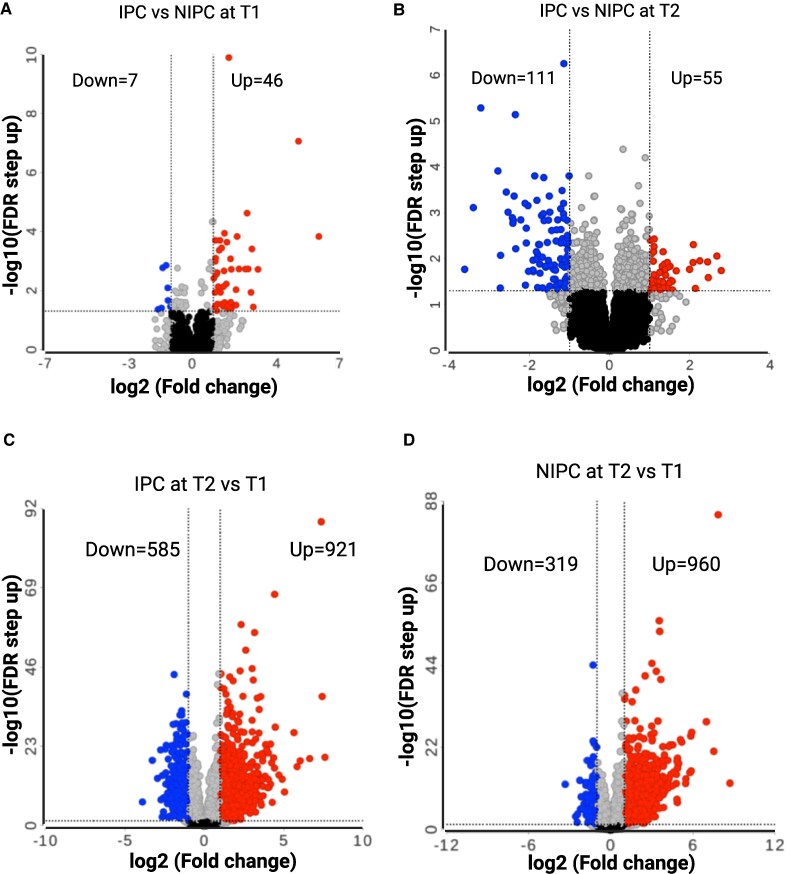
Differential gene expression analysis of transcriptomic data. (*A–D*) The Volcano plots showing number of differentially expressed genes (FC < −2, >2, false discovery rate step up <0.05) in multiple comparison analysis including ischaemic preconditioning vs. non-ischaemic preconditioning at T1, ischaemic preconditioning vs. non-ischaemic preconditioning at T2, ischaemic preconditioning at T2 vs. T1, non-ischaemic preconditioning at T2 vs. T1.

To investigate the changes in myocardial gene expression after exposure to different ischaemic conditions, multiple comparison analysis was performed. The DESeq2 analysis of the gene count files in the Partek flow software revealed differentially expressed genes (DEGs) at both time points T1 and T2 in absence or presence of IPC (*Figure 1C*). The DESeq2 analysis identified DEGs in multiple comparisons including IPC vs. NIPC at T1, IPC vs. NIPC at T2, IPC at T2 vs. T1 and NIPC at T2 vs. T1 (*Figure 2A–D*). Comparing IPC to NIPC at time points T1 and T2 identified transcriptional changes caused by IPC at an early time point and at 4 h time point post-IPC in presence of a 13.5 min prolonged ischaemia (*Figure 2A* and *B*). A total of 53 genes were differentially expressed by IPC when compared with NIPC at T1 where 7 genes were down-regulated and 46 genes were up-regulated (*Figure 2A* and *Table 1*). Seven of these DEGs were long non-coding RNAs (LncRNAs) (13.21%) (see Supplementary material online, *Figure S1* and *Table 1*). The comparison of IPC to NIPC at T2 identified a differential gene expression profile of myocardial stunning when compared with myocardial infarction (*Figure 2B*). The DESeq2 analysis identified 166 genes differentially expressed in this comparison (IPC to NIPC at T2) where 111 were down-regulated and 55 genes were up-regulated. (*Figure 2B* and *Table 2*). Fourteen of these DEGs were LncRNAs (8.43%) (see Supplementary material online, *Figure S1* and *Table 2*).

**Table 1:** Ischaemic preconditioning vs. non-ischaemic preconditioning at T1

Gene ID	Gene name	*P*-value	FDR step up	Fold change
Pdk4	Pdk4	8.37E-15	1.27E−10	3.34E + 00
ENSRNOG00000050647	—	1.15E-11	8.68E−08	3.31E + 01
Nr4a3	Nr4a3	4.75E-09	2.39E−05	6.07E + 00
Cebpd	Cebpd	4.57E-08	1.15E−04	2.88E + 00
ENSRNOG00000045654	—	7.74E-08	1.48E−04	6.46E + 01
Has1	Has1	7.83E-08	1.48E−04	4.37E + 00
Depp1	Depp1	1.25E-07	1.99E−04	2.15E + 00
Nfkbia	Nfkbia	1.32E-07	1.99E−04	2.48E + 00
Gadd45b	Gadd45b	1.68E-07	2.30E−04	3.13E + 00
Jun	Jun	2.86E-07	3.61E−04	2.63E + 00
Fam110c	Fam110c	3.37E-07	3.91E−04	7.14E + 00
Hdc	Hdc	4.01E-07	4.33E−04	2.43E + 00
Cebpb	Cebpb	7.98E-07	8.04E−04	2.16E + 00
Apold1	Apold1	8.95E-07	8.46E−04	3.53E + 00
Ddit4	Ddit4	1.16E−06	1.03E−03	2.13E + 00
ENSRNOG00000068639	—	1.77E−06	1.41E−03	−2.37E + 00
ENSRNOG00000062545	—	2.26E−06	1.70E−03	−2.65E + 00
Atf3	Atf3	2.90E−06	1.86E−03	6.31E + 00
Ptgs2	Ptgs2	3.08E−06	1.86E−03	4.66E + 00
Mgam	Mgam	3.22E−06	1.87E−03	5.76E + 00
Errfi1	Errfi1	3.41E−06	1.91E−03	3.62E + 00
Cxcl1	Cxcl1	3.56E−06	1.92E−03	8.71E + 00
Ch25h	Ch25h	4.21E−06	2.17E−03	2.85E + 00
Nr4a2	Nr4a2	5.26E−06	2.48E−03	2.26E + 00
Enc1	Enc1	6.22E−06	2.85E−03	2.19E + 00
Ifrd1	Ifrd1	8.74E−06	3.88E−03	2.03E + 00
Rasd1	Rasd1	1.47E−05	6.02E−03	3.06E + 00
Btg2	Btg2	1.85E−05	7.37E−03	2.80E + 00
ENSRNOG00000069024	—	1.95E−05	7.43E−03	2.35E + 00
ENSRNOG00000062615	—	2.27E−05	8.17E−03	−2.25E + 00
Il1b	Il1b	2.75E−05	9.43E−03	4.23E + 00
Fosl2	Fosl2	3.56E−05	1.16E−02	2.12E + 00
Retnlg	Retnlg	3.68E−05	1.16E−02	6.91E + 00
Gem	Gem	3.69E−05	1.16E−02	2.53E + 00
ENSRNOG00000067235	—	7.58E−05	2.16E−02	−2.23E + 00
Serpine1	Serpine1	9.60E−05	2.51E−02	3.56E + 00
Socs3	Socs3	1.02E−04	2.57E−02	2.91E + 00
ENSRNOG00000069551	—	1.17E−04	2.89E−02	2.65E + 00
Nfkbid	Nfkbid	1.19E−04	2.89E−02	2.29E + 00
Mmp9	Mmp9	1.42E−04	3.13E−02	4.23E + 00
Mt2A	Mt2A	1.45E−04	3.13E−02	2.84E + 00
Zfp36	Zfp36	1.59E−04	3.30E−02	3.18E + 00
Junb	Junb	1.69E−04	3.45E−02	2.98E + 00
ENSRNOG00000065682	—	1.73E−04	3.49E−02	2.80E + 00
ENSRNOG00000063198	—	1.77E−04	3.52E−02	−2.06E + 00
Sik1	Sik1	1.82E−04	3.57E−02	2.12E + 00
Cxcl2	Cxcl2	1.90E−04	3.63E−02	7.46E + 00
Rgs1	Rgs1	2.05E−04	3.88E−02	3.42E + 00
Myh7	Myh7	2.11E−04	3.94E−02	−2.75E + 00
Egr2	Egr2	2.21E−04	4.02E−02	3.62E + 00
Ptprh	Ptprh	2.51E−04	4.37E−02	−3.09E + 00
Otud1	Otud1	2.52E−04	4.37E−02	3.34E + 00
ENSRNOG00000070737	—	2.81E−04	4.82E−02	2.23E + 00

**Table 2:** Ischaemic preconditioning vs. non-ischaemic preconditioning at T2

Gene ID	Gene name	*P*-value	FDR step up	Fold change
ENSRNOG00000060846	—	3.71E−11	5.60E−07	−2.20E + 00
Tmem200c	Tmem200c	6.86E−10	5.19E−06	−9.25E + 00
Hsph1	Hsph1	1.44E−09	7.26E−06	−5.10E + 00
Npy	Npy	4.88E−08	1.23E−04	−6.88E + 00
Ntrk3	Ntrk3	7.65E−08	1.57E−04	−2.01E + 00
Fgfr4	Fgfr4	9.28E−08	1.57E−04	−3.65E + 00
Hspa1l	Hspa1l	1.13E−07	1.71E−04	−3.12E + 00
Stk32c	Stk32c	2.60E−07	3.28E−04	−2.27E + 00
ENSRNOG00000062084	—	3.05E−07	3.55E−04	−5.96E + 00
ENSRNOG00000062564	—	4.58E−07	4.33E−04	−2.84E + 00
Clec1b	Clec1b	4.04E−07	4.33E−04	−5.21E + 00
Stmn4	Stmn4	6.39E−07	5.37E−04	−3.51E + 00
G0s2	G0s2	8.50E−07	6.22E−04	−4.31E + 00
Slc5a3	Slc5a3	8.65E−07	6.22E−04	−2.22E + 00
Dnajb1	Dnajb1	1.02E−06	6.99E−04	−4.10E + 00
ENSRNOG00000050647	—	1.23E−06	7.72E−04	−1.05E + 01
Sycp2	Sycp2	1.41E−06	8.17E−04	−2.33E + 00
Dnaja1	Dnaja1	1.86E−06	9.69E−04	−2.81E + 00
AY172581.9	AY172581.9	1.85E−06	9.69E−04	−5.78E + 00
Zfand2a	Zfand2a	2.11E−06	1.06E−03	−3.14E + 00
ENSRNOG00000037658	—	2.16E−06	1.06E−03	−2.22E + 00
Cd226	Cd226	2.59E−06	1.15E−03	−3.24E + 00
Chac1	Chac1	3.06E−06	1.29E−03	−5.37E + 00
Sesn2	Sesn2	3.20E−06	1.31E−03	−2.21E + 00
ENSRNOG00000062844	—	3.69E−06	1.43E−03	−2.45E + 00
Tubb1	Tubb1	3.64E−06	1.43E−03	−4.64E + 00
Tg	Tg	4.00E−06	1.45E−03	−2.88E + 00
ENSRNOG00000064282	—	4.25E−06	1.46E−03	−5.29E + 00
Pitpnm3	Pitpnm3	4.23E−06	1.46E−03	−2.08E + 00
Mpig6b	Mpig6b	4.46E−06	1.48E−03	−3.11E + 00
Gdf15	Gdf15	5.46E−06	1.70E−03	−5.32E + 00
Treml1	Treml1	7.62E−06	2.17E−03	−4.22E + 00
ENSRNOG00000063182	—	8.49E−06	2.31E−03	−2.32E + 00
Slc6a4	Slc6a4	9.03E−06	2.35E−03	−2.50E + 00
ENSRNOG00000064726	—	9.80E−06	2.44E−03	−2.72E + 00
Slc52a3	Slc52a3	1.36E−05	3.12E−03	−2.07E + 00
Phgdh	Phgdh	1.72E−05	3.61E−03	−2.08E + 00
Ism2	Ism2	1.85E−05	3.68E−03	−2.26E + 00
Eps8l1	Eps8l1	1.98E−05	3.73E−03	−2.10E + 00
Bmp4	Bmp4	1.95E−05	3.73E−03	2.16E + 00
Enpp4	Enpp4	2.15E−05	3.96E−03	2.02E + 00
Hsp90aa1	Hsp90aa1	2.30E−05	4.14E−03	−2.52E + 00
Nptx1	Nptx1	2.50E−05	4.39E−03	−2.21E + 00
Ccdc168	Ccdc168	2.65E−05	4.44E−03	−2.93E + 00
Nes	Nes	2.77E−05	4.47E−03	−2.03E + 00
ENSRNOG00000047204	—	3.04E−05	4.57E−03	−4.22E + 00
Syt17	Syt17	3.04E−05	4.57E−03	−2.33E + 00
Il23a	Il23a	3.21E−05	4.67E−03	−2.13E + 00
Dusp5	Dusp5	3.50E−05	4.90E−03	−3.41E + 00
Phf11	Phf11	3.60E−05	4.95E−03	4.25E + 00
Tspan1	Tspan1	3.60E−05	4.95E-03	−3.36E + 00
Olah	Olah	4.12E−05	5.46E-03	2.15E + 00
ENSRNOG00000064743	—	4.75E−05	6.08E−03	−5.06E + 00
Dedd2	Dedd2	5.11E−05	6.44E−03	−2.07E + 00
Rtp4	Rtp4	5.95E−05	7.09E−03	2.14E + 00
Parp14	Parp14	5.92E−05	7.09E−03	2.52E + 00
Itga2b	Itga2b	5.86E−05	7.09E−03	−3.54E + 00
Lgals3bp	Lgals3bp	7.07E−05	7.92E−03	2.05E + 00
ENSRNOG00000068391	—	7.34E−05	8.04E−03	−3.47E + 00
Slc25a25	Slc25a25	7.94E−05	8.38E−03	−2.04E + 00
Fosb	Fosb	8.18E−05	8.46E−03	−6.59E + 00
Sox4	Sox4	8.67E−05	8.74E−03	−2.07E + 00
AC112568.1	AC112568.1	8.79E−05	8.78E−03	6.39E + 00
Pf4	Pf4	9.89E−05	9.27E−03	−3.20E + 00
Krt24	Krt24	1.12E−04	1.01E−02	−2.33E + 00
Mpzl2	Mpzl2	1.14E−04	1.02E−02	−3.54E + 00
Rimbp3	Rimbp3	1.19E−04	1.04E−02	−2.18E + 00
Ly6g6d	Ly6g6d	1.26E−04	1.09E−02	−3.76E + 00
Nanos1	Nanos1	1.29E−04	1.09E−02	−2.88E + 00
Angptl7	Angptl7	1.35E−04	1.12E−02	4.76E + 00
Wars1	Wars1	1.41E−04	1.14E−02	2.13E + 00
Rgs18	Rgs18	1.47E−04	1.17E−02	−2.14E + 00
Cxcl9	Cxcl9	1.51E−04	1.18E−02	5.44E + 00
Tmem106a	Tmem106a	1.57E−04	1.21E−02	2.71E + 00
Cxcl11	Cxcl11	1.60E−04	1.22E−02	4.23E + 00
ENSRNOG00000070694	—	1.66E−04	1.23E−02	−2.10E + 00
Ifi47	Ifi47	1.82E−04	1.31E−02	2.58E + 00
NEWGENE_2724	NEWGENE_2724	1.82E−04	1.31E−02	−3.35E + 00
Alox12	Alox12	1.92E−04	1.36E−02	−2.27E + 00
Dgki	Dgki	1.97E−04	1.38E−02	−2.16E + 00
ENSRNOG00000062599	—	2.00E−04	1.38E−02	−2.60E + 00
Sptbn4	Sptbn4	1.99E−04	1.38E−02	−2.77E + 00
ENSRNOG00000049853	—	2.00E−04	1.38E−02	−2.28E + 00
F5	F5	2.17E−04	1.46E−02	−2.80E + 00
AABR07025272.1	AABR07025272.1	2.33E−04	1.47E−02	2.48E + 00
Ifit2	Ifit2	2.26E−04	1.47E−02	2.84E + 00
Pacrg	Pacrg	2.31E−04	1.47E−02	−3.04E + 00
Ociad2	Ociad2	2.40E−04	1.49E−02	−2.07E + 00
Fam83g	Fam83g	2.50E−04	1.53E−02	−2.18E + 00
Gnaz	Gnaz	2.56E−04	1.53E−02	−3.02E + 00
Irf1	Irf1	2.57E−04	1.53E−02	2.75E + 00
Irf7	Irf7	2.98E−04	1.70E−02	2.61E + 00
ENSRNOG00000045654	—	3.07E−04	1.71E−02	−1.22E + 01
Mx1	Mx1	3.09E−04	1.71E−02	3.98E + 00
Espnl	Espnl	3.08E-04	1.71E-02	−2.63E + 00
Muc20	Muc20	3.30E−04	1.77E−02	−3.76E + 00
Tmem229a	Tmem229a	3.33E−04	1.79E−02	−2.55E + 00
Cxcl10	Cxcl10	3.43E−04	1.82E−02	6.88E + 00
MGC105567	MGC105567	3.50E−04	1.84E−02	3.16E + 00
Tnfsf18	Tnfsf18	3.69E−04	1.89E−02	−4.06E + 00
Igtp	Igtp	3.74E−04	1.92E−02	2.42E + 00
Gp9	Gp9	3.76E−04	1.92E−02	−3.73E + 00
Oas1a	Oas1a	3.98E−04	2.00E−02	2.38E + 00
Mgam	Mgam	4.25E−04	2.08E−02	−3.33E + 00
Slamf8	Slamf8	4.80E−04	2.24E−02	2.15E + 00
Gbp2	Gbp2	4.94E−04	2.29E−02	2.63E + 00
Cyp8b1	Cyp8b1	5.20E−04	2.35E−02	2.24E + 00
ENSRNOG00000025115	—	5.50E−04	2.43E−02	−2.08E + 00
Comp	Comp	6.10E−04	2.59E−02	5.51E + 00
ENSRNOG00000069461	—	6.81E−04	2.80E-02	−2.15E + 00
Rab44	Rab44	6.82E−04	2.80E−02	−2.64E + 00
Gp1bb	Gp1bb	6.79E−04	2.80E−02	−2.57E + 00
Oas1i	Oas1i	7.16E−04	2.89E−02	2.05E + 00
ENSRNOG00000029191	—	7.51E−04	2.98E−02	2.88E + 00
Lilrb3a	Lilrb3a	7.91E−04	3.09E−02	−2.15E + 00
MGC108823	MGC108823	7.99E−04	3.11E−02	2.95E + 00
Gng2	Gng2	8.02E−04	3.12E−02	2.27E + 00
Ifi44l	Ifi44l	8.09E−04	3.13E−02	2.17E + 00
Phf11b	Phf11b	8.20E−04	3.13E−02	2.48E + 00
Tap1	Tap1	8.23E−04	3.13E−02	2.37E + 00
Usp18	Usp18	8.82E−04	3.28E−02	2.52E + 00
Treml2	Treml2	9.05E−04	3.30E−02	−2.13E + 00
Irgm	Irgm	9.36E−04	3.34E−02	2.09E + 00
St8sia2	St8sia2	9.50E−04	3.38E−02	−2.35E + 00
Oasl	Oasl	9.65E−04	3.43E−02	2.80E + 00
U1	U1	9.98E−04	3.48E−02	−2.16E + 00
ENSRNOG00000067729	—	1.13E−03	3.71E−02	−2.14E + 00
Hcar2	Hcar2	1.16E−03	3.79E−02	−4.28E + 00
Mx2	Mx2	1.19E−03	3.82E−02	2.51E + 00
Lctl	Lctl	1.24E−03	3.93E−02	−2.52E + 00
AABR07025140.1	AABR07025140.1	1.27E−03	3.98E−02	2.28E + 00
ENSRNOG00000070853	—	1.27E−03	3.98E−02	−2.81E + 00
Bsn	Bsn	1.33E−03	4.09E−02	−2.28E + 00
Proz	Proz	1.34E−03	4.11E−02	−2.19E + 00
ENSRNOG00000070434	—	1.39E−03	4.17E−02	−2.16E + 00
Rnf213	Rnf213	1.39E−03	4.17E−02	2.38E + 00
Rsad2	Rsad2	1.40E−03	4.17E−02	2.50E + 00
ENSRNOG00000062907	—	1.41E−03	4.19E−02	−2.30E + 00
ENSRNOG00000065208	—	1.43E−03	4.20E−02	2.34E + 00
AABR07030544.1	AABR07030544.1	1.43E−03	4.20E−02	−2.02E + 00
Cmpk2	Cmpk2	1.42E−03	4.20E−02	2.67E + 00
AABR07038029.1	AABR07038029.1	1.43E−03	4.20E−02	−2.18E + 00
AABR07071000.1	AABR07071000.1	1.43E−03	4.20E−02	−2.56E + 00
ENSRNOG00000071198	—	1.45E−03	4.22E−02	−2.24E + 00
ENSRNOG00000070963	—	1.45E−03	4.22E−02	−2.02E + 00
ENSRNOG00000062337	—	1.49E−03	4.26E−02	−3.47E + 00
Herc6	Herc6	1.53E−03	4.33E−02	2.41E + 00
Osm	Osm	1.55E−03	4.35E−02	−3.19E + 00
Trh	Trh	1.56E−03	4.35E−02	2.53E + 00
ENSRNOG00000063744	—	1.57E−03	4.37E−02	−6.61E + 00
ENSRNOG00000066757	—	1.59E−03	4.38E−02	−2.25E + 00
Il13ra2	Il13ra2	1.58E−03	4.38E−02	2.70E + 00
Ifih1	Ifih1	1.60E−03	4.39E−02	2.03E + 00
Pcnx2	Pcnx2	1.63E−03	4.45E−02	−2.15E + 00
F10	F10	1.64E−03	4.46E−02	4.40E + 00
ENSRNOG00000063679	—	1.66E-03	4.48E-02	2.24E + 00
Xaf1	Xaf1	1.69E−03	4.49E−02	2.16E + 00
ENSRNOG00000031167	—	1.74E−03	4.58E−02	−2.03E + 00
Mpl	Mpl	1.79E−03	4.70E−02	−2.46E + 00
Oasl2	Oasl2	1.81E−03	4.72E−02	2.23E + 00
Mmp8	Mmp8	1.84E−03	4.79E−02	−2.91E + 00
Dhx58	Dhx58	1.87E−03	4.79E−02	2.41E + 00
ENSRNOG00000068187	—	1.89E−03	4.80E−02	−2.38E + 00
Gbp5	Gbp5	1.90E−03	4.83E−02	2.97E + 00
Zbp1	Zbp1	1.91E−03	4.84E−02	2.39E + 00
Ifi44	Ifi44	1.94E−03	4.86E-02	2.00E + 00

The comparison of IPC at T2 to T1 in presence and absence of prolonged 13.5 myocardial ischaemia identified 1506 DEGs where 585 genes were down-regulated and 921 genes were up-regulated (*Figure 2C* and Supplementary material online, *Table S3*). Of these 1506 DEGs, 110 (7.30%) were LncRNAs (see Supplementary material online, *Figure S1* and *Table S3*). Furthermore, the comparison of NIPC at T2 to T1 in presence and absence of prolonged 13.5 myocardial ischaemia identified transcriptional changes caused by prolonged ischaemia alone representing myocardial tissue subjected to myocardial infarction or necrosis. In this comparative analysis, the DESeq2 identified 1279 DEGs where 319 genes were down-regulated and 960 genes were up-regulated (*Figure 2D* and Supplementary material online, *Table S4*). Ninety-eight (7.66%) of these DEGs were LncRNAs (see Supplementary material online, *Figure S1* and *Table S4*). Differentially expressed genes in each comparative analysis were applied in Gene Set Enrichment Analysis (GSEA) to identify KEGG pathways significantly enriched in each comparison (see Supplementary material online, *Figures S2–S5*). The identified top enriched pathways primarily include immune-related pathways such as tumor necrosis factor signalling, IL-17 signalling and NK-kappa B signalling pathways when IPC was compared with NIPC at T1 (see Supplementary material online, *Figure S2*). At time point T2, IPC effects were observed on pathways that were primarily related to immune response to viral infections including cytokine–cytokine receptor interaction, Influenza A and RIG-I-like receptor signalling pathway (see Supplementary material online, *Figure S3*). The GSEA also discovered top enriched pathways when DEGs identified in the comparative analysis of IPC at T2 vs. T1 were used. The enriched pathways included immune-related pathways, metabolism, apoptosis, necroptosis, transcriptional misregulation, and hypoxia-related pathways (see Supplementary material online, *Figure S4*). The GSEA also identified enriched KEGG pathways in prolonged hypoxic condition when DEGs from the comparative analysis of NIPC at T2 vs. T1 were used. The enriched pathways included immune-related pathways, metabolism, apoptosis, necroptosis, transcriptional misregulation and hypoxia-related pathways with reduced enrichment score relative to IPC at T2 vs. T1 (see Supplementary material online, *Figure S5*).

### DNA methylation analysis at T1

**Figure 3:**
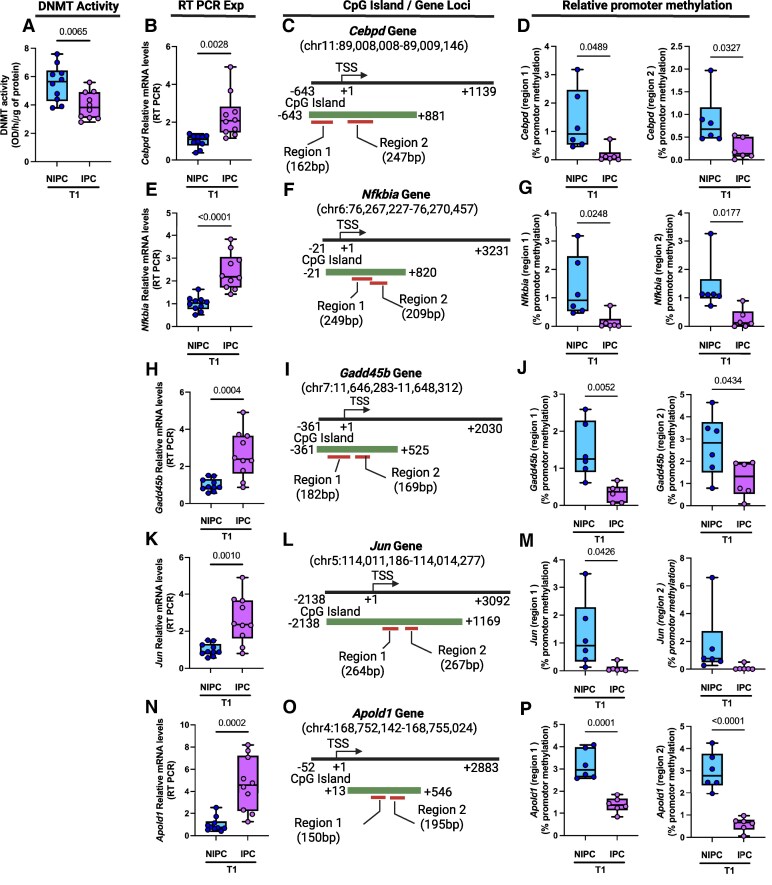
Ischaemic preconditioning effects on DNA methyltransferase activity and DNA methylation at gene promoters. (*A*) DNA methyltransferase activity of ischaemic preconditioning vs. non-ischaemic preconditioning at T1 (*n* = 10 per group. (*B*) RT-PCR data for mRNA levels of the CCAAT enhancer binding protein delta *(Cebpd)* gene (*n* = 10 per group). (*C*) Schematic diagram showing *Cebpd* genomic location, CpG island, and regions amplified with primers designed within CpG island and gene promoter region (*D*) MethylMiner quantitative PCR data showing relative promoter methylation for Regions 1 and 2 of the *Cebpd* gene (*n* = 6 per group). (*E*) RT-PCR data for mRNA levels of the NFKB inhibitor alpha *(Nfkbia*) gene (*n* = 10 per group). (*F*) Schematic diagram showing *Nfkbia* genomic location, CpG island, and regions amplified with primers designed within CpG island and gene promoter region (*G*) MethylMiner quantitative PCR data showing relative promoter methylation for Regions 1 and 2 of the *Nfkbia* gene (*n* = 6 per group). (*H*) RT-PCR data for mRNA levels of the Growth arrest and DNA damage inducible beta *(Gadd45b)* gene (*n* = 10 per group). (*I*) Schematic diagram showing *Gadd45b* genomic location, CpG island, and regions amplified with primers designed within CpG island and gene promoter region (*J*) MethylMiner quantitative PCR data showing relative promoter methylation for Regions 1 and 2 of the *Gadd45b* gene (*n* = 6 per group). (*K*) RT-PCR data for mRNA levels of the Jun proto-oncogene (*Jun*) gene (*n* = 10 per group). (*L*) Schematic diagram showing *Jun* genomic location, CpG island, and regions amplified with primers designed within CpG island and gene promoter region (*M*) MethylMiner quantitative PCR data showing relative promoter methylation for Regions 1 and 2 of the *Jun* gene (*n* = 6 per group). (*N*) RT-PCR data for mRNA levels of the Apolipoprotein L domain containing 1 (*Aplod1*) gene (*n* = 10 per group). (*O*) Schematic diagram showing *Aplod1* genomic location, CpG island, and regions amplified with primers designed within CpG island and gene promoter region (*P*) MethylMiner quantitative PCR data showing relative promoter methylation for Regions 1 and 2 of the *Aplod1* gene (*n* = 6 per group). Data are shown as mean ± SEM. *P*-value was determined using an unpaired two-tailed *t*-test.

Since DNA methylation is a very well-known epigenetic regulator of gene transcription and a recent study has shown that targeting DNA methylation can reduce cardiac injury associated with I/R, we aimed to further investigate if IPC-mediated transcriptomic changes are in part associated with DNA methylation. We measured DNMT activity in cardiac tissues collected from animals at both time points T1 and T2 and compared IPC group to the NIPC to identify IPC-mediated changes in DNMT activity. In addition, promoters of DEGs regulated by IPC at both time points T1 and T2 were analysed for DNA methylation using MethylMiner and qPCR. As a proof of principal, DEGs with highest statistical significance, fold change and higher density of CpGs in their CpG island at the gene promoter were analysed for DNA methylation at both time points T1 and T2 and compared IPC group to NIPC to identify IPC-mediated DNA methylation status of DEGs promoters. DNA methyltransferase activity was significantly reduced in cardiac tissue of rat exposed to IPC when compared with the NIPC group of animals (*Figure 3A*). Five genes were selected based on the criteria stated above to comprehensively examine the methylation status of the CpG islands at the promoter regions in the two experimental groups at T1 (*Figure 3B–P*). CCAAT enhancer binding protein delta (*Cebpd)* mRNA levels were significantly increased in the IPC group when compared with the NIPC group consistent with the RNA-seq data (*Figure 3B* and Supplementary material online, *Figure S6A*). Two different sets of primers were designed to amplify the CpG rich regions in the *Cebpd* promoter (−643/+881 bp of the transcription start site) and to comprehensively examine the methylation status of the CpG islands in the two experimental groups (*Figure 3C*). A significant reduction of DNA methylation was observed in both regions in the IPC group when compared with the NIPC group (*Figure 3D*). NFKB inhibitor alpha *(Nfkbia*) mRNA levels were significantly elevated in the IPC group when compared with NIPC group consistent with the RNA-seq data (*Figure 3E* and Supplementary material online, *Figure S6B*). Two different sets of primers were designed to amplify the CpG dense regions in the CpG island at the *Nfkbia* promoter (−21/+820 bp of the transcription start site) (*Figure 3F*). There was a significant reduction in the DNA methylation status of both amplified regions in the IPC group when compared with the NIPC group (*Figure 3G*). Growth arrest and DNA damage inducible beta *(Gadd45b)* mRNA levels were also significantly elevated in the IPC group when compared with the NIPC group consistent with the RNA-seq data (*Figure 3H* and Supplementary material online, *Figure S6C*). Two different sets of primers were used to amplify the CpG dense regions at the *Gadd45b* promoter (−361/+525 bp of the transcription start site) (*Figure 3I*). There was a significant reduction in the DNA methylation status of both amplified regions in the IPC group when compared with the NIPC group (*Figure 3J*). Jun proto-oncogene (*Jun*) mRNA levels were significantly increased in the IPC group when compared with the NIPC group and this observation was consistent with the RNA-seq data (*Figure 3K* and Supplementary material online, *Figure S6D*). Two sets of primers were used to amplify the CpG rich regions at the *Jun* promoter (−2138/+1169 bp of the transcription start site) (*Figure 3L*). A significant reduction of DNA methylation was observed in one of two regions in the IPC group when compared with the NIPC group (*Figure 3M*). Apolipoprotein L domain containing 1 (*Aplod1*) mRNA levels were significantly elevated in the IPC group when compared with the NIPC group consistent with the RNA-seq data (*Figure 3N* and Supplementary material online, *Figure S6E*). Two sets of primers were used to amplify the CpG islands at the *Aplod1* promoter (+13/+546 bp of the transcription start site) (*Figure 3O*). A significant reduction of DNA methylation was observed in both regions in the IPC group when compared with the NIPC group (*Figure 3P*).

### DNA methylation analysis at T2

**Figure 4:**
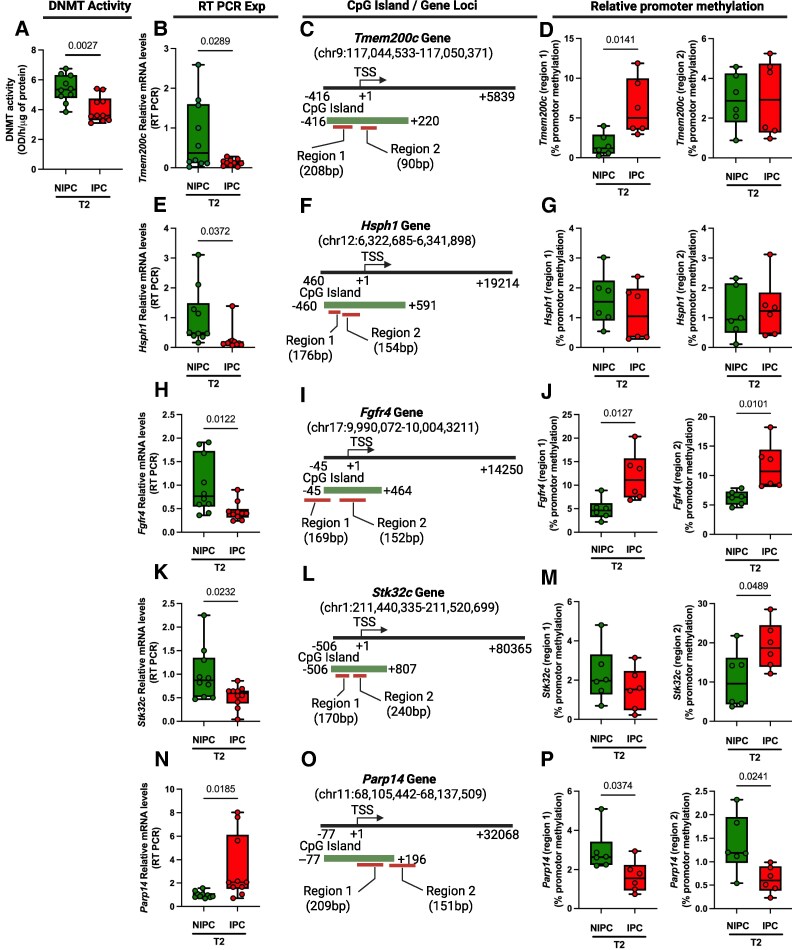
Ischaemic preconditioning effects on DNA methyltransferase activity and DNA methylation at gene promoters in presence of prolonged ischaemia. (*A*) DNA methyltransferase activity of ischaemic preconditioning vs. non-ischaemic preconditioning at T2 (*n* = 10 per group. (*B*) RT-PCR data for mRNA levels of the Transmembrane protein 200C (*Tmem200c*) gene (*n* = 10 per group). (*C*) Schematic diagram showing *Tmem200c* genomic location and CpG island (green line). Red lines indicate the CpG rich regions amplified with specific primers designed within CpG island and gene promoter (*D*) MethylMiner quantitative PCR data showing relative promoter methylation for Regions 1 and 2 of the *Tmem200c* gene (*n* = 6 per group). (*E*) RT-PCR data for mRNA levels of the Heat shock protein family H member 1 *(Hsph1*) gene (*n* = 10 per group). (*F*) Schematic diagram showing *Hsph1* genomic location and CpG island (green line). Red lines indicate the CpG rich regions amplified with specific primers designed within CpG island and gene promoter (*G*) MethylMiner quantitative PCR data showing relative promoter methylation for Regions 1 and 2 of the *Hsph1* gene (*n* = 6 per group). (*H*) RT-PCR data for mRNA levels of the Fibroblast growth factor receptor 4 *(Fgfr4)* gene (*n* = 10 per group). (*I*) Schematic diagram showing *Fgfr4* genomic location and CpG island (green line). Red lines indicate the CpG rich regions amplified with specific primers designed within CpG island and gene promoter. (*J*) MethylMiner quantitative PCR data showing relative promoter methylation for Regions 1 and 2 of the *Fgfr4* gene (*n* = 6 per group). (*K*) RT-PCR data for mRNA levels of the Serine/Threonine kinase 32C (*Stk32c*) gene (*n* = 10 per group). (*L*) Schematic diagram showing *Stk32* genomic location and CpG island (line underneath). Red lines indicate the CpG rich regions amplified with specific primers designed within CpG island and gene promoter (*M*) MethylMiner quantitative PCR data showing relative promoter methylation for Regions 1 and 2 of the *Stk32* gene (*n* = 6 per group). (*N*) RT-PCR data for mRNA levels of the Poly (ADP-Ribose) polymerase family member 14 (*Parp14*) gene (*n* = 10 per group). (*O*) Schematic diagram showing *Parp14* genomic location and CpG island (green line). Red lines indicate the CpG rich regions amplified with specific primers designed within CpG island and gene promoter (*P*) MethylMiner quantitative PCR data showing relative promoter methylation for Regions 1 and 2 of the *Parp14* gene (*n* = 6 per group). Data are shown as mean ± SEM. *P*-value was determined using an unpaired two-tailed *t*-test.

We next measured DNMT activity in cardiac tissues collected from animals at T2 and compared the IPC group to the NIPC group to identify IPC-mediated changes in DNMT activity at this specific time point. DNA methyltransferase activity was significantly reduced in cardiac tissue of rat exposed to IPC when compared with the NIPC group of animals (*Figure 4A*). In addition, promoters of DEGs regulated by IPC at T2 were analysed for DNA methylation using MethylMiner and qPCR. Similar to T1, five genes were selected based on the criteria stated above to comprehensively examine the methylation status of the CpG dense islands at the promoter regions in the two experimental groups at T2 (*Figure 4B–P*). Transmembrane protein 200C (*Tmem200c)* mRNA levels were significantly reduced in the IPC group when compared with the NIPC group consistent with the RNA-seq data (*Figure 4B* and Supplementary material online, *Figure S6F*). Two different sets of primers were designed to amplify the CpG rich regions in the *Tmem200c* promoter (−416/+220 bp of the transcription start site) and to comprehensively examine the methylation status of the CpG islands in the two experimental groups (*Figure 4C*). A significant increase of DNA methylation was observed in one of the investigated regions in the IPC group when compared with the NIPC group (*Figure 4D*). Heat shock protein family H member 1 *(Hsph1*) mRNA levels were significantly down-regulated in the IPC group when compared with the NIPC group consistent with the RNA-seq data (*Figure 4E* and Supplementary material online, *Figure S6G*). Two different sets of primers were designed to amplify the CpG dense regions in the CpG island at the *Hsph1* promoter (−460/+591 bp of the transcription start site) (*Figure 4F*). There were no significant changes in the DNA methylation status of both amplified regions in the IPC group when compared with the NIPC group (*Figure 4G*). Fibroblast growth factor receptor 4 *(Fgfr4)* mRNA levels were also significantly decreased in the IPC group when compared with NIPC group consistent with the RNA-seq data (*Figure 4H* and Supplementary material online, *Figure S6H*). Two different sets of primers were used to amplify the CpG dense regions at the *Fgfr4* promoter (−45/+464 bp of the transcription start site) (*Figure 4I*). There was a significant increase in the DNA methylation status of both amplified regions in the IPC group when compared with the NIPC group (*Figure 4J*). Serine/Threonine kinase 32C (*Stk32c*) mRNA levels were significantly reduced in the IPC group when compared with the NIPC group and this observation was consistent with the RNA-seq data (*Figure 4K* and Supplementary material online, *Figure S6I*). Two sets of primers were used to amplify the CpG rich regions at the *Stk32c* promoter (−506/+807 bp of the transcription start site) (*Figure 4L*). A significant increase of DNA methylation was observed in one of two regions in the IPC group when compared with the NIPC group (*Figure 4M*). Poly (ADP-Ribose) polymerase family member 14 (*Parp14*) mRNA levels were significantly increased in the IPC group when compared with the NIPC group consistent with the RNA-seq data (*Figure 4N* and Supplementary material online, *Figure S6J*). Two sets of primers were used to amplify the CpG islands at the *Parp14* promoter (−77/+196 bp of the transcription start site) (*Figure 4O*). A significant reduction of DNA methylation was observed in both regions in the IPC group when compared with the NIPC group (*Figure 4P*).

### Ischaemic preconditioning modulates temporal Dnmt1 binding to gene promoters

**Figure 5:**
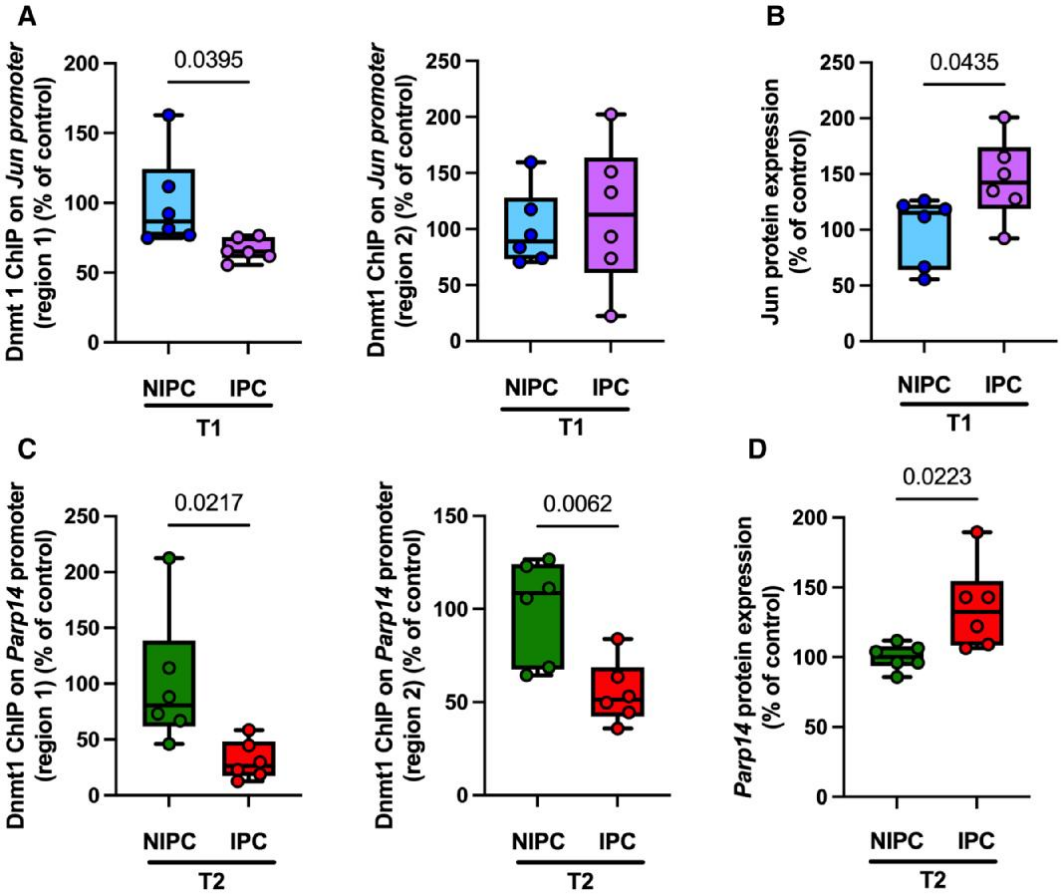
Ischaemic preconditioning reduces Dnmt1 occupancy on gene promoters and increases protein expression. (*A*) Chromatin immunoprecipitation analysis of Dnmt1 binding at selected promoter regions of *Jun*. (*B*) Enzyme-linked immunosorbent assay quantification of Jun protein post-ischaemic preconditioning. (*C*) Chromatin immunoprecipitation analysis of Dnmt1 binding at selected promoter regions of *Papr14*. (*D*) Enzyme-linked immunosorbent assay quantification of Parp14 protein post-ischaemic preconditioning. Data represent mean ± SEM. *P*-value was determined using an unpaired two-tailed *t*-test.

Given Dnmt1’s well-established role in maintaining DNA methylation, we next examined its promoter occupancy on DEGs at both time points following IPC. As a proof of principal, we performed ChIP assays targeting Dnmt1 at the promoters of two transcription factors, *Jun* and *Parp14*, both of which exhibited increased mRNA expression and reduced promoter DNA methylation in response to IPC. Dnmt1 binding was significantly reduced at one of the two examined regions within the *Jun* promoter at the early time point (T1) (*Figure 5A*). Consistent with the transcriptional upregulation observed via RT-PCR and RNA-seq, ELISA analysis confirmed elevated *Jun* protein levels following IPC (*Figure 5B*). Similarly, at the later reperfusion phase (T2), Dnmt1 occupancy was significantly reduced at both selected regions of the *Parp14* promoter post-IPC (*Figure 5C*). This reduction corresponded with a marked increase in *Parp14* protein levels as confirmed by ELISA, corroborating the transcriptomic data which showed increased *Parp14* expression post-IPC (*Figure 5D*). These findings collectively demonstrate that IPC influences gene regulation by modulating Dnmt1 promoter binding at both early (T1) and later (T2) time points, leading to transcriptional activation and consistent increases in protein expression.

## Discussion

The most important finding of this study is that IPC induced significant transcriptomic changes during both early (T1) and prolonged (T2) reperfusion phases, regulating critical genes involved in inflammation, oxidative stress, apoptosis, and DNA repair. We also identified differentially expressed LncRNAs, highlighting their potential as novel regulators in cardio-protection. In addition, IPC mediates cardio-protection through dynamic epigenetic changes, particularly DNA methylation modifications driven by reduced DNMT activity. As a proof of principal, we show expression of top DEGs is influenced by the status of DNA methylation at their promoters. These findings collectively underscore IPC’s role in reprogramming the cardiac transcriptome and epigenome, providing new insights into its therapeutic potential.

In rodent models, the conventional ischaemic occlusion time to induce myocardial infarction is typically 30 min.^12^ However, this longer duration often results in large infarcts, making it less suitable for studying the link between IPC and myocardial stunning. To overcome this challenge, we developed a novel I/R model with a 13.5-min ischaemic period, specifically aimed at investigating the relationship between infarct size, reversible contractile dysfunction, and IPC.^5^ This model consistently induces significant myocardial infarction in non-preconditioned animals, as confirmed by TTC staining.^5^ Importantly, IPC significantly reduces infarct size and helps preserve myocardial function, even with the shorter ischaemic duration. Furthermore, our study also reveals that IPC accentuates myocardial stunning, a reversible contractile dysfunction without permanent damage.^5^ This suggests that transient dysfunction is protective, associated with cell viability and recovery.^5,13^ These findings may shift clinical perspectives on post-reperfusion stunning, advocating for supportive management of transient dysfunction in ischaemia-reperfusion settings. Further studies in large animals and humans are needed to optimize IPC protocols and improve clinical outcomes.

While a previous study demonstrated that IPC modulates DNA methylation to protect the heart from I/R injury using an *ex vivo* Langendorff setup with 30 min of ischaemia followed by 60 min of reperfusion,^12^ our research focuses on a different concept. Specifically, we aimed to understand the molecular relationship between IPC, myocardial stunning, and necrosis using a novel rat I/R model. In our previous work, we demonstrated that IPC protects against necrosis by intensifying myocardial stunning, a phenomenon traditionally considered detrimental, but which may actually play a protective role in preventing necrosis.^5^ This study takes this a step further by exploring the transcriptomic and epigenetic changes triggered by IPC that enhance stunning while concurrently protecting against necrosis, an effect that, to our knowledge, has not been demonstrated by any prior study. Furthermore, our study provides a comprehensive transcriptome wide signature of gene expression changes associated with IPC during both early and prolonged reperfusion phases, which contrasts with the limited RT-PCR-based gene expression analysis of a few genes reported in the previous study.^12^ Most importantly, our findings demonstrate that IPC induces specific changes in the DNA methylation status of gene promoters, influencing their expression. This differs significantly from the previous study, which only reported global DNA methylation changes without identifying specific gene promoter modifications.^12^

Notably, we utilized an *in vivo* model of I/R injury, whereas the prior study employed an *ex vivo* approach with rat hearts. Additionally, our research provides a comprehensive transcriptomic signature of gene expression changes associated with IPC during both early and prolonged reperfusion phases, unlike that previous study which reported only RT-PCR-based gene expression changes of a few genes. Importantly, our study demonstrates that IPC induces changes in the DNA methylation status of gene promoters, affecting their expression, unlike that previous study which reported only global DNA methylation changes. Our findings demonstrate that IPC induces distinct DNA methylation patterns associated with myocardial stunning, an important consideration is whether these epigenetic changes are model-specific or represent fundamental IPC mechanisms. Previous studies using conventional IPC models (30-min ischaemia) have similarly reported global DNA methylation changes,^12^ suggesting common epigenetic regulation across different ischaemic durations. However, our novel 13.5-min model uniquely reveals how these modifications specifically favour reversible stunning over necrosis, a distinction not captured in longer ischaemia protocols. The coordinated DNMT suppression and promoter-specific methylation changes we observed (particularly at stress response and inflammation genes like *Cebpd* and *Nfkbia*) provide mechanistic plausibility for these findings. Future studies comparing epigenetic signatures across IPC models may help identify core protective pathways vs. context-dependent adaptations, potentially informing targeted therapies for different ischaemic injury phenotypes.

Ischaemic preconditioning may protect the heart by regulating gene expression across multiple phases of reperfusion. In early reperfusion (T1), IPC up-regulates key genes involved in multiple pathways including statistically top DEGs like *Cebpd, Nfkbia*, *Gadd45b*, *Jun*, and *Aplod1*, which help reduce inflammation, repair DNA, and support stress adaptation.^11,14–17^ In the later phase of reperfusion (T2), IPC suppresses harmful key genes involved in multiple pathways including statistically top DEGs such as *Tmem200c*, *Fgfr4*, *Stk32c*, and *Hsph1*, to prevent maladaptive responses.^18–21^  *Parp14* was the top most of 55 up-regulated genes in this comparative analysis. Additionally, the study identifies LncRNAs among the DEGs. While their specific roles remain to be elucidated, their presence underscores the potential importance of non-coding RNA in IPC-mediated cardioprotection. This finding aligns with emerging evidence that LncRNAs play pivotal roles in regulating cardiac gene expression and could serve as novel therapeutic targets.^22^

DNA methylation plays a key role in regulating gene expression, particularly during IPC.^23^ By studying DNA methylation changes and DNMT activity, we can better understand how IPC modulates genes involved in stress, inflammation, and repair, ultimately enhancing cardiac protection during ischaemic injury. Therefore, we next investigate how IPC regulates gene expression through dynamic DNA methylation changes driven by reduced DNMT activity. At T1, cardiac tissue from IPC-treated animals showed significantly lower DNMT activity compared with the NIPC group, facilitating selective hypomethylation of key genes and their activation, underscoring IPC’s role in shaping the cardiac transcriptome for cardio-protection. As a proof of principal, we selected top DEGs and investigated DNA methylation status at their promoter region. At T1, *Cebpd* (a key player in stress responses, including inflammation and apoptosis regulation),^14^  *Nfkbia* (gene encoding IκBα, an inhibitor of the inflammatory nuclear factor-kappa light-chain-enhancer of activated B cells),^16^  *Gadd45b* (regulates DNA repair, cell cycle arrest, and anti-apoptotic pathways),^15^  *Jun* (part of the activator protein 1 transcription factor complex involved in stress responses),^11^ and *Aplod1* (involved in angiogenesis and regulation of endothelial permeability)^17^ were investigated for promoter DNA methylation. By reducing DNMT activity and promoter methylation, IPC activated expression of these critical genes which may support cellular repair and survival under ischaemic stress,^14^ reduces inflammation and mitigates excessive inflammatory signalling during ischaemia-reperfusion injury,^16^ and helps to preserve myocardial integrity during ischaemic stress,^15^ and counteract oxidative damage, support vascular stability, and maintain energy homeostasis, thereby promoting cardiac resilience under stress conditions.^17,24^ This analysis shows how reduced DNMT activity in IPC lowers DNA methylation, activating genes involved in inflammation (*Nfkbia*), DNA repair (*Gadd45b*), stress response (*Cebpd* and *Jun*), and metabolism (*Aplod1*).^11,14–17^ These observations support the role of reduced DNMT activity in upregulating protective genes. This selective gene regulation by IPC may enhance myocardial recovery, offering the potential for DNMT-targeted therapies to mimic IPC's protective effects in ischaemic heart disease. Further mechanistic studies are necessary to fully elucidate the functional roles of these genes in ischaemia-reperfusion injury. In addition, we recognize the importance of establishing causal links between the epigenetic modifications observed and the cardioprotective effects of IPC. While the current study focused on *in vivo* profiling of DNA methylation and transcriptional changes in IPC-treated myocardium, mechanistic dissection of individual targets was beyond its scope. Nevertheless, several hypomethylated and up-regulated genes identified, such as *Gadd45b*, *Parp14*, *Nfkbia*, and *Cebpd*, are well-documented mediators of cardioprotection, DNA repair, and inflammation regulation.^14–16^ Their coordinated hypomethylation and transcriptional activation, alongside hypermethylation and repression of maladaptive genes like *Fgfr4*,^18^ suggest a non-random, targeted epigenetic response to IPC. Furthermore, the observed global reduction in DNMT activity preceding gene expression changes supports a potential causal relationship. However, definitive evidence will require future mechanistic studies, including isoform-specific DNMT inhibition, targeted epigenetic editing (e.g. dCas9-based methylation tools), and rescue experiments to determine whether reversing these modifications alters gene expression and cardiac outcomes.^25^ These approaches represent promising next steps to move beyond correlation and clarify the functional role of epigenetic regulation in IPC-mediated cardioprotection.

DNA methyltransferase activity remained significantly lower at the later time point in the cardiac tissue of IPC-treated animals compared with the NIPC group, indicating a consistent suppression of DNA methylation. At T2, however, the gene regulatory pattern became more complex, with certain genes showing hypermethylation linked to reduced expression despite reduction in global DNMT activity. This suggests a potential phase-specific adjustment in IPC’s epigenetic response. Importantly, this phase also distinguishes the IPC-treated group, which experienced myocardial stunning, from the non-IPC group, which developed myocardial necrosis. Since myocardial stunning is reversible and DNA methylation is also a reversible process, it raises the possibility of a connection between the two. Further research is needed to explore whether altering epigenetic mechanisms could modify the outcome from necrosis to stunning or vice versa. A recent study by Guida *et al*.^26^ further supports the functional significance of DNA methylation in ischaemic contexts, although in the brain. In that work, stroke-induced recruitment of the DNMT1/MeCP2/REST complex led to hypermethylation of the Ncx1 heart promoter in neurons, repressing its transcription and contributing to neuronal injury. Although this regulatory mechanism was observed in cerebral tissue, it illustrates how ischaemic stress can target heart-relevant gene promoters via methylation, even outside of cardiac tissue. In our study, we observed IPC-induced hypomethylation of several gene promoters in the myocardium, most notably *Nfkbia*, *Gadd45b*, and *Ju*n, accompanied by increased transcription. Unlike the hypermethylation-mediated repression observed in the brain, the hypomethylation in our IPC model appears to facilitate expression of genes associated with DNA repair, anti-inflammatory signalling, and stress adaptation, all consistent with the protective phenotype of IPC. Together, these findings underscore the organ and context-specific role of DNA methylation in modulating ischaemic responses and suggest that dynamic epigenetic regulation, whether suppressive or permissive, may be a shared feature of adaptive and maladaptive outcomes in ischaemia.

Like T1, we selected the top DEGs (down-regulated: *Tmem200c*, *Hsph1* (involved in protein folding and stress responses),^21^  *Fgfr4* (chronic kidney disease and age-related left ventricular hypertrophy),^18^  *Stk32c* (a kinase involved in cellular signalling)^19^ and up-regulated: *Parp14* (linked to DNA repair and stress adaptation)),^20^ and investigated DNA methylation status at their promoter region at T2. In the later phase of reperfusion at T2, upregulation of *Parp14* and promoter hypomethylation was consistent with reduced global DNMT activity. Interestingly, the *Hsph1 gene* showed lower mRNA levels in the IPC group, but without significant changes in its promoter methylation. This underscores that IPC down-regulates *Hsph1* through mechanisms independent of DNA methylation, such as histone modifications or shifts in transcription factor activity. Further studies are warranted to investigate methylation independent effects of IPC on the transcriptome. At T2, despite reduced global DNMT activity in IPC, we observed selective hypermethylation of specific gene promoters (e.g. *Tmem200c*, *Fgfr4*, *Stk32c*). This reflects that while global DNMT activity is reduced, gene-specific DNA hypermethylation supresses gene expression. IPC appears to finely regulate gene expression through DNA methylation, suppressing harmful pathways and activating protective ones, thus optimizing myocardial recovery. To further elucidate the mechanistic basis of IPC-induced gene activation, we conducted ChIP assays to evaluate Dnmt1 occupancy at gene promoters. Our results demonstrate that IPC markedly reduces Dnmt1 binding at the *Jun* promoter during early reperfusion (T1) and at the *Parp14* promoter during late reperfusion (T2). These time-specific reductions in promoter occupancy were accompanied by hypomethylation, increased mRNA expression, and elevated protein levels of both genes, supporting a direct link between Dnmt1 displacement and transcriptional activation. Notably, *Jun* and *Parp14* are key regulators of stress response and DNA repair pathways,^11,20^ highlighting their relevance to IPC-mediated cardioprotection. Collectively, these findings provide direct evidence that IPC modulates gene expression through temporally distinct epigenetic regulation of Dnmt1 binding, adding a novel dimension to the protective mechanisms of IPC during distinct phases of reperfusion.

Although we did not perform direct statistical correlations between transcriptomic or epigenetic changes and cardiac function in this study, multiple lines of evidence suggest a biologically meaningful relationship. First, the temporal patterns of gene regulation align with established phases of IPC: early upregulation of protective genes such as *Cebpd* and *Nfkbia* at T1 corresponds with the initial window of cardioprotection, while downregulation of maladaptive genes like *Fgfr4* at T2 coincides with the resolution of myocardial stunning. Second, our previous study using this IPC model^5^ demonstrated reduced infarct size and preserved ejection fraction, confirming functional cardioprotection *in vivo*. Third, the DEGs are enriched in pathways essential for cardiac recovery, including inflammation, oxidative stress response, angiogenesis, and DNA repair.

### Limitations of the study

Despite its strengths, this study has several limitations (i) while transcriptomic and methylation data are robust, the descriptive nature of the study requires additional studies to confirm these findings at the protein level and assess post-translational modifications. (ii) The focus on early and intermediate time points prevents an understanding of IPC’s long-term effects on cardiac structure and function. (iii) Although the rat model is well-suited for mechanistic studies, differences between rodent and human cardiac physiology necessitate caution in extrapolating these findings. (iv) Our focus on early and intermediate time points precludes insights into long-term cardiac remodelling post-IPC. Given the pivotal role of the identified transcriptomic and epigenetic changes in acute adaptation, their influence on later remodelling warrants further investigation using this unique model.

## Conclusions

In conclusion, this study provides valuable insights into the complex mechanisms underlying IPC and its role in myocardial protection. The findings suggest that IPC regulates a broad spectrum of genes involved in inflammation, DNA repair, stress response, and metabolism through dynamic epigenetic changes, particularly altered DNMT activity and associated promoter DNA methylation. These changes may facilitate the selective activation of protective genes and the suppression of maladaptive pathways, optimizing myocardial recovery and reducing necrosis and infarct size. Further mechanistic studies are required to confirm these findings at the protein level and explore the long-term effects of IPC on cardiac function.

## Lead author biography


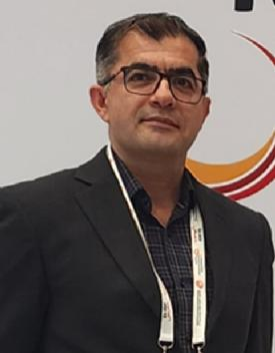


Dr Abdul Waheed Khan is a research fellow at the Department of Diabetes, School of Translational Medicine, Monash University, Australia. He holds a PhD in Biomedical Sciences from the University of Melbourne and has postdoctoral training from the Karolinska Institute, Sweden. His research focuses on molecular biology, epigenetics, and transcription factors particularly in diabetes-associated cardiovascular disease. Dr Khan has secured multiple competitive funding. His current research interests explore novel therapeutic strategies including drug repurposing, with a strong emphasis on translational impact.

## Supplementary Material

oeaf124_Supplementary_Data

## Data Availability

The authors declare that all data are available with the article and the data supplement. All sequencing data sets in this article are deposited in international public repository, Gene Expression Omnibus, under accession identification as GSE284606.

## References

[1] Buja LM . Pathobiology of myocardial ischemia and reperfusion injury: models, modes, molecular mechanisms, modulation, and clinical applications. Cardiol Rev 2023;31:252–264. 35175958 10.1097/CRD.0000000000000440 PMC10399947

[2] Iliodromitis EK, Lazou A, Kremastinos DT. Ischemic preconditioning: protection against myocardial necrosis and apoptosis. Vasc Health Risk Manag 2007;3:629–637. 18078014 PMC2291307

[3] Kloner RA, Jennings RB. Consequences of brief ischemia: stunning, preconditioning, and their clinical implications: part 1. Circulation 2001;104:2981–2989. 11739316 10.1161/hc4801.100038

[4] Carmeliet E . Myocardial ischemia: reversible and irreversible changes. Circulation 1984;70:149–151. 6327116 10.1161/01.cir.70.1.149

[5] Elmahdy A, Shekka Espinosa A, Kakaei Y, Pylova T, Jha A, Zulfaj E, Krasnikova M, Al-Awar A, Sheybani Z, Sevastianova V, Berger E, Nejat A, Molander L, Andersson EA, Omerovic E, Hussain S, Redfors B. Ischemic preconditioning affects phosphosites and accentuates myocardial stunning while reducing infarction size in rats. Front Cardiovasc Med 2024;11:1376367. 38559672 10.3389/fcvm.2024.1376367 PMC10978780

[6] Wang K, Li Y, Qiang T, Chen J, Wang X. Role of epigenetic regulation in myocardial ischemia/reperfusion injury. Pharmacol Res 2021;170:105743. 34182132 10.1016/j.phrs.2021.105743

[7] Kaipananickal H, Waheed Khan A, Okabe J, Corcoran SJ, Esler MD, El-Osta A. Targeting treatment refractory NET by EZH2 inhibition in postural tachycardia syndrome. Circ Res 2020;126:1058–1060. 32122262 10.1161/CIRCRESAHA.119.315654

[8] Jiang H, Lei R, Ding SW, Zhu S. Skewer: a fast and accurate adapter trimmer for next-generation sequencing paired-end reads. BMC Bioinformatics 2014;15:182. 24925680 10.1186/1471-2105-15-182 PMC4074385

[9] Dobin A, Davis CA, Schlesinger F, Drenkow J, Zaleski C, Jha S, Batut P, Chaisson M, Gingeras TR. STAR: ultrafast universal RNA-Seq aligner. Bioinformatics 2013;29:15–21. 23104886 10.1093/bioinformatics/bts635 PMC3530905

[10] Streese L, Khan AW, Deiseroth A, Hussain S, Suades R, Tiaden A, Kyburz D, Cosentino F, Hanssen H. High-intensity interval training modulates retinal microvascular phenotype and DNA methylation of p66Shc gene: a randomized controlled trial (EXAMIN AGE). Eur Heart J 2020;41:1514–1519. 31323685 10.1093/eurheartj/ehz196

[11] Hussain S, Khan AW, Akhmedov A, Suades R, Costantino S, Paneni F, Caidahl K, Mohammed SA, Hage C, Gkolfos C, Bjorck H, Pernow J, Lund LH, Luscher TF, Cosentino F. Hyperglycemia induces myocardial dysfunction via epigenetic regulation of JunD. Circ Res 2020;127:1261–1273. 32815777 10.1161/CIRCRESAHA.120.317132

[12] Boovarahan SR, Kurian GA. Ischemic preconditioning modulates the DNA methylation process of the rat heart to provide tolerance to withstand ischemia reperfusion injury and its associated mitochondrial dysfunction. 3 Biotech 2024;14:121. 10.1007/s13205-024-03965-0 PMC10965879 38550905

[13] Kalil-Filho R, de Albuquerque CP, Weiss RG, Mocelim A, Bellotti G, Cerri G, Pileggi F. Normal high energy phosphate ratios in “stunned” human myocardium. J Am Coll Cardiol 1997;30:1228–1232. 9350920 10.1016/s0735-1097(97)00306-9

[14] Huang GN, Thatcher JE, McAnally J, Kong Y, Qi X, Tan W, DiMaio JM, Amatruda JF, Gerard RD, Hill JA, Bassel-Duby R, Olson EN. C/EBP transcription factors mediate epicardial activation during heart development and injury. Science 2012;338:1599–1603. 23160954 10.1126/science.1229765 PMC3613149

[15] Liu B, Zhang YH, Jiang Y, Li LL, Chen Q, He GQ, Tan XD, Li CQ. Gadd45b is a novel mediator of neuronal apoptosis in ischemic stroke. Int J Biol Sci 2015;11:353–360. 25678854 10.7150/ijbs.9813 PMC4323375

[16] Song KY, Zhang XZ, Li F, Ji QR. Silencing of ATP2B1-AS1 contributes to protection against myocardial infarction in mouse via blocking NFKBIA-mediated NF-kappaB signalling pathway. J Cell Mol Med 2020;24:4466–4479. 32155320 10.1111/jcmm.15105 PMC7176878

[17] Stritt S, Nurden P, Nurden AT, Schved JF, Bordet JC, Roux M, Alessi MC, Tregouet DA, Makinen T, Giansily-Blaizot M. APOLD1 loss causes endothelial dysfunction involving cell junctions, cytoskeletal architecture, and Weibel-Palade bodies, while disrupting hemostasis. Haematologica 2023;108:772–784. 35638551 10.3324/haematol.2022.280816 PMC9973481

[18] Grabner A, Schramm K, Silswal N, Hendrix M, Yanucil C, Czaya B, Singh S, Wolf M, Hermann S, Stypmann J, Di Marco GS, Brand M, Wacker MJ, Faul C. FGF23/FGFR4-mediated left ventricular hypertrophy is reversible. Sci Rep 2017;7:1993. 28512310 10.1038/s41598-017-02068-6 PMC5434018

[19] Sun E, Liu K, Zhao K, Wang L. Serine/threonine kinase 32C is overexpressed in bladder cancer and contributes to tumor progression. Cancer Biol Ther 2019;20:307–320. 30359551 10.1080/15384047.2018.1529098 PMC6370379

[20] Tang Y, Liu J, Wang Y, Yang L, Han B, Zhang Y, Bai Y, Shen L, Li M, Jiang T, Ye Q, Yu X, Huang R, Zhang Z, Xu Y, Yao H. PARP14 inhibits microglial activation via LPAR5 to promote post-stroke functional recovery. Autophagy 2021;17:2905–2922. 33317392 10.1080/15548627.2020.1847799 PMC8525999

[21] Kraemer BF, Mannell H, Lamkemeyer T, Franz-Wachtel M, Lindemann S. Heat-shock protein 27 (HSPB1) is upregulated and phosphorylated in human platelets during ST-elevation myocardial infarction. Int J Mol Sci 2019;20:5968. 31783528 10.3390/ijms20235968 PMC6928972

[22] Bar C, Chatterjee S, Thum T. Long noncoding RNAs in cardiovascular pathology, diagnosis, and therapy. Circulation 2016;134:1484–1499. 27821419 10.1161/CIRCULATIONAHA.116.023686

[23] Cai M, Zhu Y, Li Z, Josephs-Spaulding J, Zhou Y, Hu Y, Chen H, Liu Y, He W, Zhang J. Profiling the gene expression and DNA methylation in the mouse brain after ischemic preconditioning. Neuroscience 2019;406:249–261. 30902679 10.1016/j.neuroscience.2019.03.023

[24] Bates DO, Harper SJ. Regulation of vascular permeability by vascular endothelial growth factors. Vascul Pharmacol 2002;39:225–237. 12747962 10.1016/s1537-1891(03)00011-9

[25] Cai R, Lv R, Shi X, Yang G, Jin J. CRISPR/dCas9 tools: epigenetic mechanism and application in gene transcriptional regulation. Int J Mol Sci 2023;24:14865. 37834313 10.3390/ijms241914865 PMC10573330

[26] Guida N, Serani A, Sanguigno L, Mascolo L, Cuomo O, Fioriniello S, Marano D, Ragione FD, Anzilotti S, Brancaccio P, Molinaro P, Pignataro G, Annunziato L, Formisano L. Stroke causes DNA methylation at Ncx1 heart promoter in the brain via DNMT1/MeCP2/REST epigenetic complex. J Am Heart Assoc 2024;13:e030460. 38456444 10.1161/JAHA.123.030460 PMC11010005

