# Supplementary material for: Ischaemic preconditioning regulates cardiac transcriptome via DNA methylation conferring cardio-protection from ischaemic reperfusion injury
Source: Eur Heart J Open. 2025 Oct 10;5(5):oeaf124. doi: 10.1093/ehjopen/oeaf124 (PMC12541389; doi:10.1093/ehjopen/oeaf124)
Supplement: oeaf124_Supplementary_Data [file oeaf124_supplementary_data.zip › Supp Fig 2.pdf]

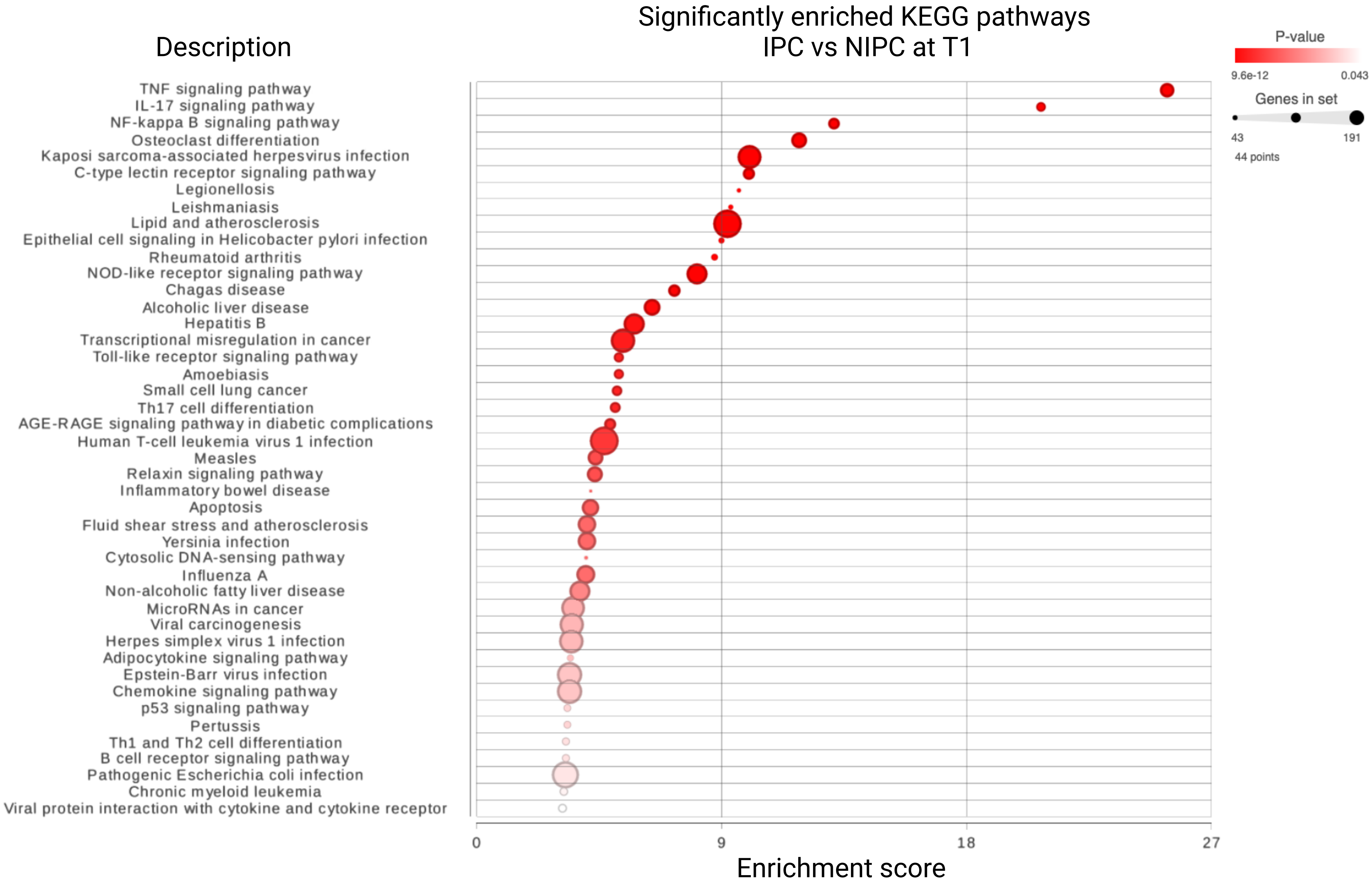
Significantly enriched KEGG pathways
IPC vs NIPC at T1
Description
TNF signaling pathway
IL-17 signaling pathway
NF-kappa B signaling pathway
Osteoclast differentiation
Kaposi sarcoma-associated herpesvirus infection
C-type lectin receptor signaling pathway
Legionellosis
Leishmaniasis
Lipid and atherosclerosis
Epithelial cell signaling in Helicobacter pylori infection
Rheumatoid arthritis
NOD-like receptor signaling pathway
Chagas disease
Alcoholic liver disease
Hepatitis B
Transcriptional misregulation in cancer
Toll-like receptor signaling pathway
Amoebiasis
Small cell lung cancer
Th17 cell differentiation
AGE-RAGE signaling pathway in diabetic complications
Human T-cell leukemia virus 1 infection
Measles
Relaxin signaling pathway
Inflammatory bowel disease
Apoptosis
Fluid shear stress and atherosclerosis
Yersinia infection
Cytosolic DNA-sensing pathway
Influenza A
Non-alcoholic fatty liver disease
MicroRNAs in cancer
Viral carcinogenesis
Herpes simplex virus 1 infection
Adipocytokine signaling pathway
Epstein-Barr virus infection
Chemokine signaling pathway
p53 signaling pathway
Pertussis
Th1 and Th2 cell differentiation
B cell receptor signaling pathway
Pathogenic Escherichia coli infection
Chronic myeloid leukemia
Viral protein interaction with cytokine and cytokine receptor
P-value
9.6e-12
0.043
Genes in set
43
191
44 points
0
9
18
27
Enrichment score
