# Supplementary material for: Ischaemic preconditioning regulates cardiac transcriptome via DNA methylation conferring cardio-protection from ischaemic reperfusion injury
Source: Eur Heart J Open. 2025 Oct 10;5(5):oeaf124. doi: 10.1093/ehjopen/oeaf124 (PMC12541389; doi:10.1093/ehjopen/oeaf124)
Supplement: oeaf124_Supplementary_Data [file oeaf124_supplementary_data.zip › Supp Fig 4.pdf]

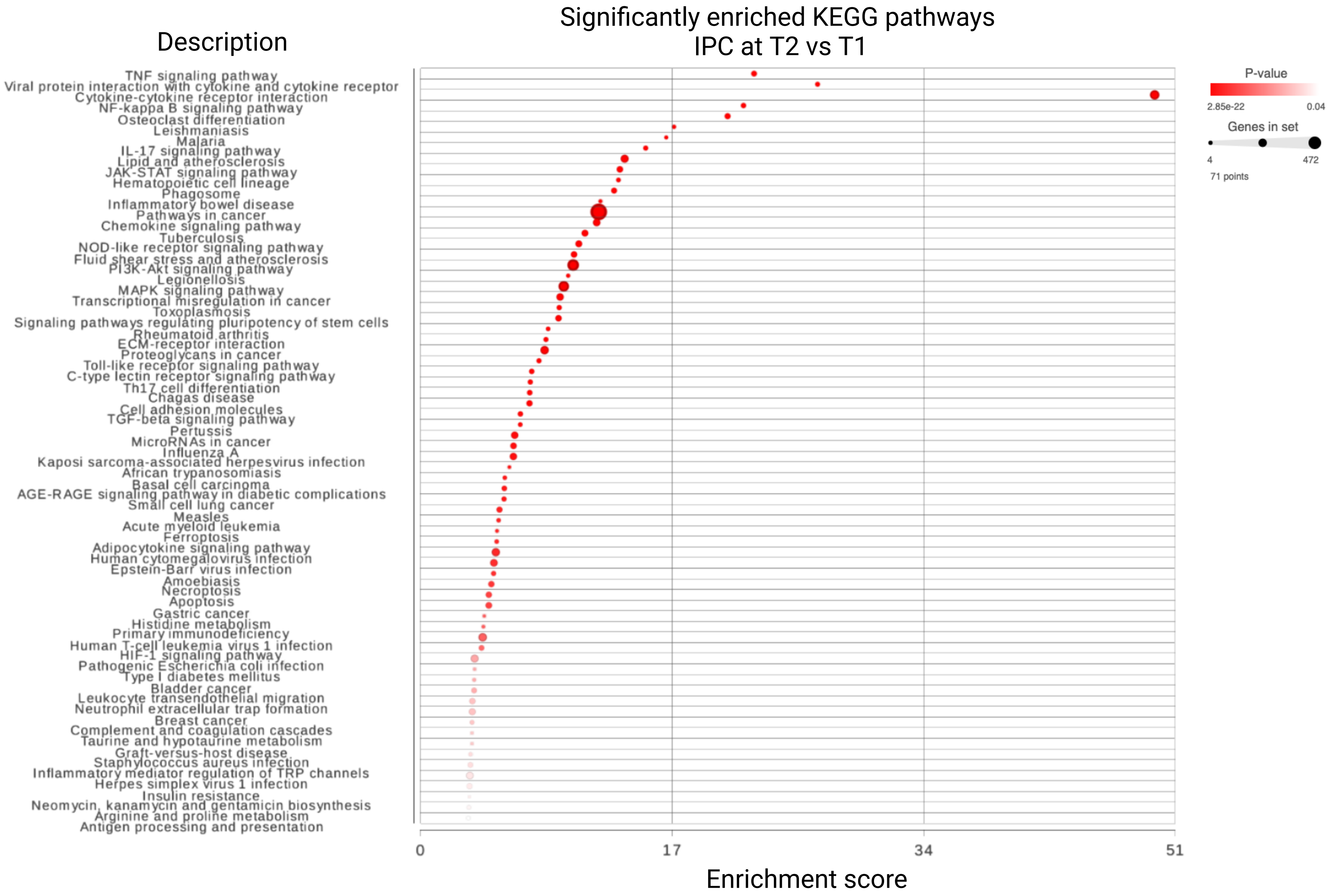

Significantly enriched KEGG pathways
IPC at T2 vs T1
Description
TNF signaling pathway
Viral protein interaction with cytokine and cytokine receptor
Cytokine-cytokine receptor interaction
NF-kappa B signaling pathway
Osteoclast differentiation
Leishmaniasis
Malaria
IL-17 signaling pathway
Lipid and atherosclerosis
JAK-STAT signaling pathway
Hematopoietic cell lineage
Phagosome
Inflammatory bowel disease
Pathways in cancer
Chemokine signaling pathway
Tuberculosis
NOD-like receptor signaling pathway
Fluid shear stress and atherosclerosis
PI3K-Akt signaling pathway
Legionellosis
MAPK signaling pathway
Transcriptional misregulation in cancer
Toxoplasmosis
Signaling pathways regulating pluripotency of stem cells
Rheumatoid arthritis
ECM-receptor interaction
Proteoglycans in cancer
Toll-like receptor signaling pathway
C-type lectin receptor signaling pathway
Th17 cell differentiation
Chagas disease
Cell adhesion molecules
TGF-beta signaling pathway
Pertussis
MicroRNAs in cancer
Influenza A
Kaposi sarcoma-associated herpesvirus infection
African trypanosomiasis
Basal cell carcinoma
AGE-RAGE signaling pathway in diabetic complications
Small cell lung cancer
Measles
Acute myeloid leukemia
Ferroptosis
Adipocytokine signaling pathway
Human cytomegalovirus infection
Epstein-Barr virus infection
Amoebiasis
Necroptosis
Apoptosis
Gastric cancer
Histidine metabolism
Primary immunodeficiency
Human T-cell leukemia virus 1 infection
HIF-1 signaling pathway
Pathogenic Escherichia coli infection
Type I diabetes mellitus
Bladder cancer
Leukocyte transendothelial migration
Neutrophil extracellular trap formation
Breast cancer
Complement and coagulation cascades
Taurine and hypotaurine metabolism
Graft-versus-host disease
Staphylococcus aureus infection
Inflammatory mediator regulation of TRP channels
Herpes simplex virus 1 infection
Insulin resistance
Neomycin, kanamycin and gentamicin biosynthesis
Arginine and proline metabolism
Antigen processing and presentation
0
17
34
51
Enrichment score
P-value
2.85e-22
0.04
Genes in set
4
472
71 points
