# Supplementary material for: Ischaemic preconditioning regulates cardiac transcriptome via DNA methylation conferring cardio-protection from ischaemic reperfusion injury
Source: Eur Heart J Open. 2025 Oct 10;5(5):oeaf124. doi: 10.1093/ehjopen/oeaf124 (PMC12541389; doi:10.1093/ehjopen/oeaf124)
Supplement: oeaf124_Supplementary_Data [file oeaf124_supplementary_data.zip › Supp Fig 5.pdf]

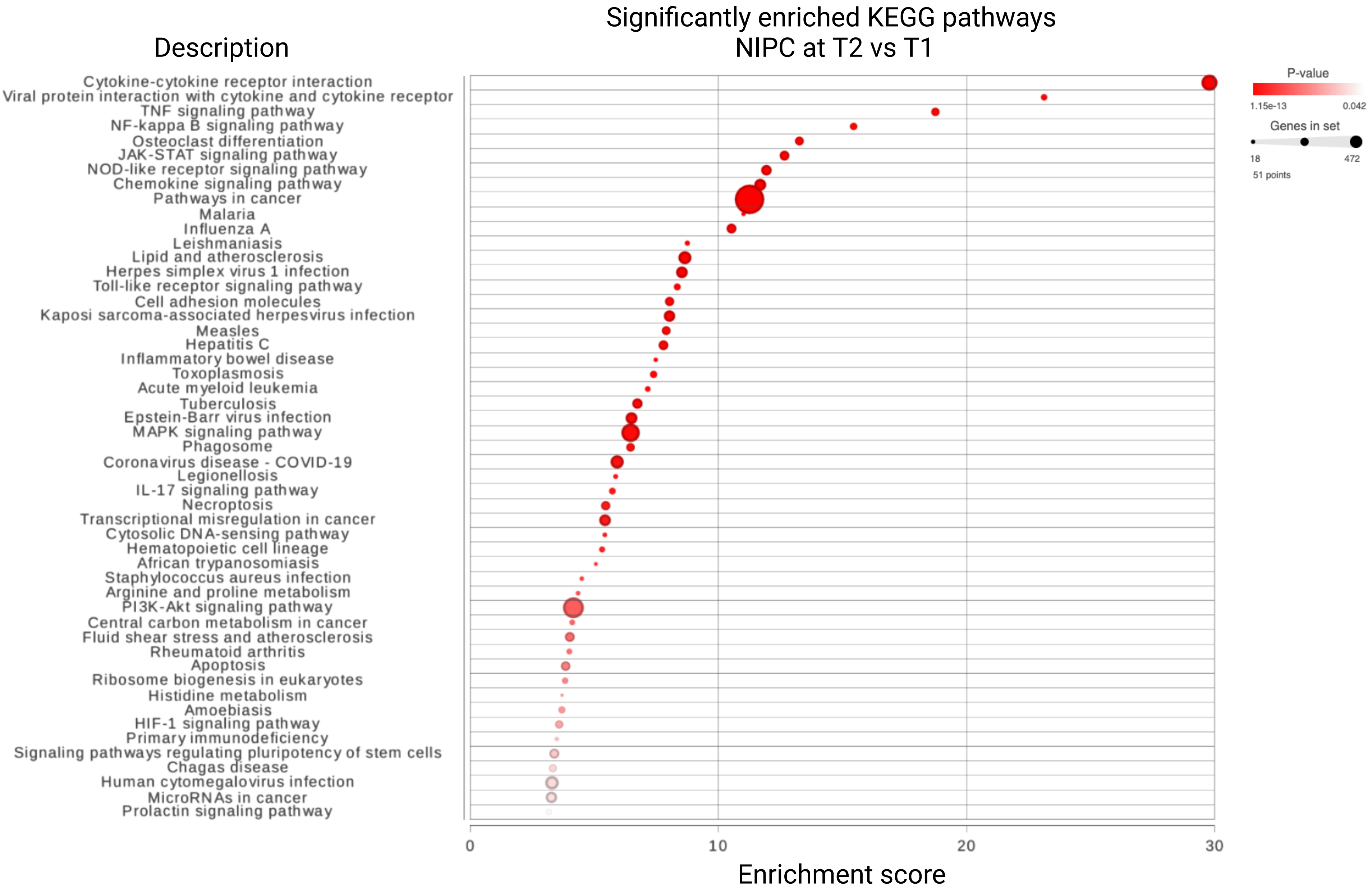
Significantly enriched KEGG pathways
NIPC at T2 vs T1
Description
Cytokine-cytokine receptor interaction
Viral protein interaction with cytokine and cytokine receptor
TNF signaling pathway
NF-kappa B signaling pathway
Osteoclast differentiation
JAK-STAT signaling pathway
NOD-like receptor signaling pathway
Chemokine signaling pathway
Pathways in cancer
Malaria
Influenza A
Leishmaniasis
Lipid and atherosclerosis
Herpes simplex virus 1 infection
Toll-like receptor signaling pathway
Cell adhesion molecules
Kaposi sarcoma-associated herpesvirus infection
Measles
Hepatitis C
Inflammatory bowel disease
Toxoplasmosis
Acute myeloid leukemia
Tuberculosis
Epstein-Barr virus infection
MAPK signaling pathway
Phagosome
Coronavirus disease - COVID-19
Legionellosis
IL-17 signaling pathway
Necroptosis
Transcriptional misregulation in cancer
Cytosolic DNA-sensing pathway
Hematopoietic cell lineage
African trypanosomiasis
Staphylococcus aureus infection
Arginine and proline metabolism
PI3K-Akt signaling pathway
Central carbon metabolism in cancer
Fluid shear stress and atherosclerosis
Rheumatoid arthritis
Apoptosis
Ribosome biogenesis in eukaryotes
Histidine metabolism
Amoebiasis
HIF-1 signaling pathway
Primary immunodeficiency
Signaling pathways regulating pluripotency of stem cells
Chagas disease
Human cytomegalovirus infection
MicroRNAs in cancer
Prolactin signaling pathway
0
10
20
30
Enrichment score
P-value
1.15e-13
0.042
Genes in set
18
472
51 points
