# Supplementary material for: Ischaemic preconditioning regulates cardiac transcriptome via DNA methylation conferring cardio-protection from ischaemic reperfusion injury
Source: Eur Heart J Open. 2025 Oct 10;5(5):oeaf124. doi: 10.1093/ehjopen/oeaf124 (PMC12541389; doi:10.1093/ehjopen/oeaf124)
Supplement: oeaf124_Supplementary_Data [file oeaf124_supplementary_data.zip › Supp Fig legends.docx]

**Supp Fig legends.**

**Supp Fig 1.**

Percentage of differentially expressed long non-coding RNAs (LncRNAs) and protein coding genes.

**Supp Fig 2.**

The Gene Set Enrichment Analysis (GSEA) identified enriched pathways include immune-related pathways such as TNF signalling, IL-17 signalling and NK-Kappa B signalling pathways when IPC was compared to NIPC at time point 1.

**Supp Fig 3.**

The Gene Set Enrichment Analysis (GSEA) identified enriched pathways include immune-related responses to viral infections such as cytokine-cytokine receptor interaction, Influenza A and RIG-I-like receptor signalling pathway when IPC was compared to NIPC at time point 1.

**Supp Fig 4.**

The Gene Set Enrichment Analysis (GSEA) identified enriched pathways include immune-related pathways, metabolism, apoptosis, necroptosis, transcriptional mis-regulation and hypoxia-related pathways when IPC was compared to NIPC at time point 2.

**Supp Fig 5.**

The Gene Set Enrichment Analysis (GSEA) identified enriched pathways when DEGs from the comparative analysis of NIPC at T2 vs T1 were used which included immune-related pathways, metabolism, apoptosis, necroptosis, transcriptional mis-regulation and hypoxia-related pathways with reduced enrichment score relative to IPC at T2 vs T1.

**Supp Fig 6. RNA-seq data of DEGs under investigation for DNA methylation mediated gene regulation by IPC.**

RNA sequencing data (CPM, counts per million) for the **A**) CCAAT enhancer binding protein delta (*Cebpd)*, **B**) NFKB inhibitor alpha *(Nfkbia*), **C**) Growth arrest and DNA damage inducible beta *(Gadd45b),* **D**) Jun proto-oncogene (*Jun*), **E**) Apolipoprotein L domain containing 1 (*Aplod1*), **F**) Transmembrane protein 200C (*Tmem200c*), **G**) Heat shock protein family H member 1 *(Hsph1*), **H**) Fibroblast growth factor receptor 4 *(Fgfr4)* ,**I**) Serine/Threonine kinase 32C (*Stk32c*), **J**) Poly (ADP-Ribose) polymerase family member 14 (*Parp14*).
