# Supplementary material for: Ischaemic preconditioning regulates cardiac transcriptome via DNA methylation conferring cardio-protection from ischaemic reperfusion injury
Source: Eur Heart J Open. 2025 Oct 10;5(5):oeaf124. doi: 10.1093/ehjopen/oeaf124 (PMC12541389; doi:10.1093/ehjopen/oeaf124)
Supplement: oeaf124_Supplementary_Data [file oeaf124_supplementary_data.zip › Supp Table S1.docx]

**Supp Table S1**

| **Gene name** | **Forward sequence** | **Reverse sequence** |
| --- | --- | --- |
| *Cebpd* | AATGGTAGCGTTTTCTACG | AAAGTCTGTCGGAAAAGTC |
| *Nfkbia* | CAGAATTCACGGAAGATGAG | CCACTTTCCTCTTATAACGTC |
| *Gadd45b* | AGGAGGATGATATTGCTCTG | AATCTGTATGACAGTTCGTG |
| *Jun* | AAAAGTGAAAACCTTGAAAGC | CGTGGTTCATGACTTTCTG |
| *Apold1* | AAAAAGCCATCTTCTGACTC | ACTCTTTCTCTGGCTTCTG |
| *Tmem200c* | AAAACAAACAAGCAAAGTCG | CGGGGCAAAGAAAAATAGG |
| *Hsph1* | ATGGCATTTTCACCATATCC | ATTTTTATCCACATCCGAGC |
| *Fgfr4* | CTGTGAAGATGCTGAAAGAC | GTTCTTGTGTCTTCCGATTAG |
| *Skt32c* | TACATGAACAAGCAGCAATG | GGAATGAATACCAGAGGTTC |
| *Parp14* | ACACAAGGTATCTATGGGAG | CCCCTGATTTGAGATTAAACG |
