# Supplementary material for: Ischaemic preconditioning regulates cardiac transcriptome via DNA methylation conferring cardio-protection from ischaemic reperfusion injury
Source: Eur Heart J Open. 2025 Oct 10;5(5):oeaf124. doi: 10.1093/ehjopen/oeaf124 (PMC12541389; doi:10.1093/ehjopen/oeaf124)
Supplement: oeaf124_Supplementary_Data [file oeaf124_supplementary_data.zip › Supp Table S2.docx]

**Supp Table S2**

| **Gene name** | **Forward sequence** | **Reverse sequence** |
| --- | --- | --- |
| *Cebpd-R1* | CCAAGACAGAAGCCATGGA | CCTTCCTGTTTGTGCGGTTT |
| *Cebpd-R2* | TTTTCAGCCTAGACAGCCCA | TTGAAGAGGTCGGCGAAGA |
| *Nfkbia-R1* | TCTGCTCGTAATCCTCGTCC | ATCCCCTGCCAGCGTTTATA |
| *Nfkbia-R2* | TCTGCTCGTAATCCTCGTCC | GAGCAGATGGTGAAGGAGCT |
| *Gadd45b-R1* | TCCAAGAAGACGGAGGTGAC | CCTTTCCTACCTCCAGCCA |
| *Gadd45b-R2* | TACTCACTTCTGAACCGCGT | GAAAGCCTCGGACACTTCT |
| *Jun-R1* | CCGGGACTTGTGAGCTTCTT | GTGTTGAGCTCAGGCTGGAT |
| *Jun-R2* | GAGGGCATCGTCGTAGAAGG | CCAACCAACGTGAGTGCAAG |
| *Apold1-R1* | TTATCACTCAGCCCGGTCAC | GATCTCTTGCACCCTCCGTA |
| *Apold1-R2* | ATCTTCTGCAATTCCCGGGA | GCCGAAGAAGACGATGAAGT |
| *Tmem200c-R1* | CGGTTGCAGGTGTCATCTAC | TAAGAGACCGGAGCCATGAC |
| *Tmem200c-R2* | ACTCTTGGAAATGGCTCCCG | CGAGAACGGCTGGACAGT |
| *Hsph1-R1* | GGATCTACCGGCCAGACTAC | ATCGGGAACTTCTGTGGCTT |
| *Hsph1-R2* | ACAGAAGTTCCCGATCCGTT | GAGGCCTGAGTTACCATGGT |
| *Fgfr4-R1* | CAGAGTTAGAGGGTCGGCTC | GACATTCCTGGCTCTTCGG |
| *Fgfr4-R2* | TTCTGATCTGAGAGGCGTGG | CAAGGTGGTGTAGAGCAAAGA |
| *Skt32c-R1* | GGCGAGCAATCAGGAATTCC | CGCACGGCAGCTTAAATACT |
| *Skt32c-R2* | GCTCCTAGTACTGGCTCTGG | TAGCATCTGTCCTCTCTCGC |
| *Parp14-R1* | AATGTACTTCCAGAGCCGGA | ATTCTGCCTCCACGATCTCC |
| *Parp14-R2* | GGAGATCGTGGAGGCAGAAT | ATTCTTTTCCTGCCCCACCT |
