# Supplementary material for: Ischaemic preconditioning regulates cardiac transcriptome via DNA methylation conferring cardio-protection from ischaemic reperfusion injury
Source: Eur Heart J Open. 2025 Oct 10;5(5):oeaf124. doi: 10.1093/ehjopen/oeaf124 (PMC12541389; doi:10.1093/ehjopen/oeaf124)
Supplement: oeaf124_Supplementary_Data [file oeaf124_supplementary_data.zip › Supp Table S4.docx]

**Supp Table S6: NIPC at T2 vs T1**

| Gene ID | Gene name | P-value | FDR step up | Fold change |
| --- | --- | --- | --- | --- |
| Mt2A | Mt2A | 3.68E-89 | 5.56E-85 | 2.30E+02 |
| Hmox1 | Hmox1 | 1.75E-60 | 1.32E-56 | 1.17E+01 |
| Pdk4 | Pdk4 | 1.92E-57 | 9.67E-54 | 1.19E+01 |
| Sbno2 | Sbno2 | 9.08E-49 | 3.43E-45 | 8.06E+00 |
| Lysmd4 | Lysmd4 | 3.23E-48 | 9.76E-45 | -2.44E+00 |
| Fcgr2b | Fcgr2b | 1.86E-46 | 4.69E-43 | 1.00E+01 |
| Cd14 | Cd14 | 3.66E-45 | 7.91E-42 | 5.72E+00 |
| AC128848.1 | AC128848.1 | 3.71E-44 | 7.00E-41 | 1.27E+01 |
| Tinagl1 | Tinagl1 | 2.88E-41 | 4.84E-38 | 3.55E+00 |
| Igf1r | Igf1r | 9.33E-40 | 1.28E-36 | 2.02E+00 |
| Gabarapl1 | Gabarapl1 | 1.06E-38 | 1.33E-35 | 2.03E+00 |
| Rab30 | Rab30 | 5.80E-38 | 6.75E-35 | 2.96E+00 |
| Pla1a | Pla1a | 7.42E-33 | 8.02E-30 | 3.40E+00 |
| Fgf2 | Fgf2 | 1.00E-32 | 1.01E-29 | 1.09E+01 |
| Glul | Glul | 1.22E-32 | 1.09E-29 | 2.19E+00 |
| Ptx3 | Ptx3 | 1.80E-32 | 1.51E-29 | 1.27E+02 |
| Chi3l1 | Chi3l1 | 2.84E-31 | 2.15E-28 | 7.96E+00 |
| Tiam1 | Tiam1 | 1.53E-30 | 1.10E-27 | 4.45E+00 |
| Thbs1 | Thbs1 | 1.57E-29 | 1.08E-26 | 6.05E+01 |
| Csf2rb | Csf2rb | 2.45E-29 | 1.61E-26 | 8.97E+00 |
| C3 | C3 | 6.91E-29 | 4.35E-26 | 7.65E+00 |
| Selp | Selp | 1.69E-28 | 9.82E-26 | 5.76E+01 |
| Msr1 | Msr1 | 2.79E-28 | 1.53E-25 | 4.31E+00 |
| Stat3 | Stat3 | 6.69E-28 | 3.46E-25 | 2.51E+00 |
| Cpne8 | Cpne8 | 6.86E-28 | 3.46E-25 | 3.18E+00 |
| ENSRNOG00000024479 | --- | 1.07E-27 | 5.21E-25 | 5.35E+00 |
| Cebpd | Cebpd | 1.51E-27 | 7.13E-25 | 8.22E+00 |
| Serpine1 | Serpine1 | 2.06E-27 | 9.17E-25 | 3.41E+01 |
| Myd88 | Myd88 | 2.06E-27 | 9.17E-25 | 2.88E+00 |
| Bcat1 | Bcat1 | 4.39E-27 | 1.90E-24 | 8.12E+00 |
| Wdr6 | Wdr6 | 5.21E-27 | 2.19E-24 | -2.44E+00 |
| Olfm4 | Olfm4 | 1.08E-26 | 4.42E-24 | 3.64E+01 |
| Tmem44 | Tmem44 | 2.28E-26 | 9.07E-24 | -2.32E+00 |
| Tnfrsf1a | Tnfrsf1a | 3.60E-26 | 1.40E-23 | 2.19E+00 |
| Ccl22 | Ccl22 | 6.28E-26 | 2.37E-23 | 2.03E+01 |
| Cxcr2 | Cxcr2 | 6.53E-26 | 2.41E-23 | 2.31E+01 |
| Dnajb4 | Dnajb4 | 1.32E-25 | 4.76E-23 | 4.04E+00 |
| Slc3a2 | Slc3a2 | 1.58E-25 | 5.56E-23 | 2.14E+00 |
| Rnd1 | Rnd1 | 1.91E-25 | 6.57E-23 | 1.88E+01 |
| Myc | Myc | 2.14E-25 | 7.18E-23 | 1.40E+01 |
| Ell | Ell | 2.47E-25 | 8.12E-23 | 2.19E+00 |
| Nrp1 | Nrp1 | 3.07E-25 | 9.86E-23 | -2.04E+00 |
| Fkbp5 | Fkbp5 | 8.57E-25 | 2.70E-22 | 3.92E+00 |
| Acvr1b | Acvr1b | 9.03E-25 | 2.78E-22 | 2.63E+00 |
| Ifngr1 | Ifngr1 | 1.14E-24 | 3.45E-22 | 2.25E+00 |
| Gda | Gda | 2.12E-24 | 6.28E-22 | 2.31E+00 |
| ENSRNOG00000050647 | --- | 4.37E-24 | 1.27E-21 | 1.85E+02 |
| Sdc4 | Sdc4 | 1.73E-23 | 4.85E-21 | 5.64E+00 |
| Osmr | Osmr | 2.10E-23 | 5.77E-21 | 6.29E+00 |
| Gfpt2 | Gfpt2 | 3.40E-23 | 9.17E-21 | 6.25E+00 |
| Has1 | Has1 | 4.06E-23 | 1.08E-20 | 1.51E+01 |
| ENSRNOG00000062480 | --- | 7.03E-23 | 1.83E-20 | 8.39E+00 |
| Fgl2 | Fgl2 | 8.44E-23 | 2.13E-20 | 7.69E+00 |
| Cblb | Cblb | 8.37E-23 | 2.13E-20 | 2.71E+00 |
| Slc7a5 | Slc7a5 | 1.01E-22 | 2.51E-20 | 7.74E+00 |
| Ly75 | Ly75 | 1.10E-22 | 2.68E-20 | 4.25E+00 |
| Cdo1 | Cdo1 | 1.16E-22 | 2.77E-20 | 4.02E+00 |
| ENSRNOG00000065519 | --- | 1.36E-22 | 3.20E-20 | 3.40E+00 |
| Prdm16 | Prdm16 | 1.61E-22 | 3.74E-20 | -2.41E+00 |
| Cxcl16 | Cxcl16 | 3.12E-22 | 7.15E-20 | 3.45E+00 |
| S100a8 | S100a8 | 3.72E-22 | 8.40E-20 | 2.94E+01 |
| Bcl3 | Bcl3 | 4.51E-22 | 1.00E-19 | 5.90E+00 |
| Map3k6 | Map3k6 | 7.52E-22 | 1.65E-19 | 5.10E+00 |
| Xdh | Xdh | 7.80E-22 | 1.68E-19 | 2.96E+00 |
| Ptger2 | Ptger2 | 8.19E-22 | 1.74E-19 | 1.00E+01 |
| Slc11a1 | Slc11a1 | 8.90E-22 | 1.87E-19 | 3.60E+00 |
| Afap1l2 | Afap1l2 | 9.49E-22 | 1.96E-19 | -2.50E+00 |
| Ncf4 | Ncf4 | 9.94E-22 | 2.03E-19 | 3.81E+00 |
| Socs3 | Socs3 | 1.58E-21 | 3.18E-19 | 1.37E+01 |
| Trem1 | Trem1 | 1.73E-21 | 3.45E-19 | 1.81E+01 |
| Cited4 | Cited4 | 1.88E-21 | 3.70E-19 | -3.28E+00 |
| Dmp1 | Dmp1 | 1.94E-21 | 3.77E-19 | 4.18E+00 |
| Cebpb | Cebpb | 2.08E-21 | 3.98E-19 | 4.39E+00 |
| Gadd45a | Gadd45a | 2.87E-21 | 5.43E-19 | 4.84E+00 |
| S100a9 | S100a9 | 2.98E-21 | 5.56E-19 | 2.88E+01 |
| Midn | Midn | 3.80E-21 | 7.01E-19 | 2.84E+00 |
| Ncald | Ncald | 1.14E-20 | 2.02E-18 | -2.20E+00 |
| Zfand2a | Zfand2a | 1.15E-20 | 2.02E-18 | 1.09E+01 |
| Mmp8 | Mmp8 | 1.22E-20 | 2.13E-18 | 3.10E+01 |
| Cd177 | Cd177 | 1.85E-20 | 3.18E-18 | 3.05E+01 |
| Sele | Sele | 2.17E-20 | 3.68E-18 | 1.43E+01 |
| Baz1a | Baz1a | 2.28E-20 | 3.82E-18 | 2.64E+00 |
| Csf1 | Csf1 | 2.45E-20 | 4.07E-18 | 5.82E+00 |
| Clec10a | Clec10a | 2.66E-20 | 4.37E-18 | 2.79E+00 |
| Hp | Hp | 2.85E-20 | 4.63E-18 | 8.36E+00 |
| Uhrf1bp1l | Uhrf1bp1l | 4.28E-20 | 6.88E-18 | 2.51E+00 |
| ENSRNOG00000061530 | --- | 4.71E-20 | 7.42E-18 | -2.73E+00 |
| Serpinb1a | Serpinb1a | 5.08E-20 | 7.91E-18 | 7.28E+00 |
| Adamts9 | Adamts9 | 6.84E-20 | 1.04E-17 | 3.01E+00 |
| Tnfrsf12a | Tnfrsf12a | 7.77E-20 | 1.17E-17 | 1.43E+01 |
| Atp8b1 | Atp8b1 | 9.05E-20 | 1.35E-17 | 2.83E+00 |
| Mthfd2 | Mthfd2 | 9.36E-20 | 1.39E-17 | 4.21E+00 |
| Pcare | Pcare | 1.58E-19 | 2.29E-17 | 2.59E+00 |
| Mocos | Mocos | 2.08E-19 | 2.96E-17 | 2.25E+00 |
| ENSRNOG00000031167 | --- | 2.61E-19 | 3.65E-17 | 9.25E+00 |
| Mmp9 | Mmp9 | 2.73E-19 | 3.78E-17 | 2.95E+01 |
| Maff | Maff | 3.05E-19 | 4.19E-17 | 5.65E+00 |
| Ifnlr1 | Ifnlr1 | 3.34E-19 | 4.54E-17 | 4.59E+00 |
| Itgam | Itgam | 4.28E-19 | 5.77E-17 | 3.85E+00 |
| Inhbb | Inhbb | 5.78E-19 | 7.66E-17 | 1.08E+01 |
| Gfap | Gfap | 6.24E-19 | 8.21E-17 | 6.89E+00 |
| Ern1 | Ern1 | 8.31E-19 | 1.07E-16 | 3.29E+00 |
| Retnlg | Retnlg | 1.76E-18 | 2.22E-16 | 5.87E+01 |
| Il6st | Il6st | 2.02E-18 | 2.50E-16 | 2.06E+00 |
| Pla2g4a | Pla2g4a | 3.04E-18 | 3.70E-16 | 4.04E+00 |
| Fign | Fign | 3.33E-18 | 4.01E-16 | -2.11E+00 |
| Nuak1 | Nuak1 | 3.35E-18 | 4.01E-16 | 2.05E+00 |
| Chka | Chka | 3.75E-18 | 4.46E-16 | 2.19E+00 |
| Mcemp1 | Mcemp1 | 3.85E-18 | 4.55E-16 | 2.43E+01 |
| Igfbp3 | Igfbp3 | 5.08E-18 | 5.86E-16 | 2.41E+00 |
| Fhad1 | Fhad1 | 5.42E-18 | 6.21E-16 | 5.52E+01 |
| Eif1a | Eif1a | 5.55E-18 | 6.30E-16 | 3.15E+00 |
| Slc7a6 | Slc7a6 | 8.89E-18 | 9.96E-16 | 2.19E+00 |
| Hspa8 | Hspa8 | 1.00E-17 | 1.11E-15 | 2.64E+00 |
| Timp1 | Timp1 | 1.37E-17 | 1.50E-15 | 8.31E+00 |
| Hilpda | Hilpda | 1.56E-17 | 1.69E-15 | 3.33E+00 |
| Clec4d | Clec4d | 1.72E-17 | 1.84E-15 | 2.10E+01 |
| Chac1 | Chac1 | 1.77E-17 | 1.88E-15 | 2.78E+01 |
| Orai2 | Orai2 | 2.30E-17 | 2.40E-15 | 2.20E+00 |
| Il1rn | Il1rn | 2.41E-17 | 2.49E-15 | 1.02E+01 |
| Siah2 | Siah2 | 2.46E-17 | 2.52E-15 | 2.74E+00 |
| Pim3 | Pim3 | 2.50E-17 | 2.53E-15 | 2.62E+00 |
| Cst7 | Cst7 | 2.54E-17 | 2.56E-15 | 2.71E+00 |
| Runx1 | Runx1 | 2.74E-17 | 2.72E-15 | 5.17E+00 |
| Slc1a5 | Slc1a5 | 2.85E-17 | 2.80E-15 | 6.20E+00 |
| Fcnb | Fcnb | 2.98E-17 | 2.90E-15 | 9.39E+00 |
| Upp1 | Upp1 | 4.84E-17 | 4.63E-15 | 3.86E+00 |
| Capn5 | Capn5 | 5.60E-17 | 5.32E-15 | -2.47E+00 |
| H6pd | H6pd | 6.40E-17 | 6.01E-15 | 2.17E+00 |
| Zfp189 | Zfp189 | 6.44E-17 | 6.01E-15 | 2.09E+00 |
| Olr1 | Olr1 | 6.85E-17 | 6.35E-15 | 1.39E+01 |
| Tet1 | Tet1 | 7.93E-17 | 7.31E-15 | -2.00E+00 |
| Asns | Asns | 1.01E-16 | 9.16E-15 | 4.53E+00 |
| Il18rap | Il18rap | 1.65E-16 | 1.50E-14 | 3.29E+00 |
| Oscar | Oscar | 1.89E-16 | 1.70E-14 | 1.40E+01 |
| Hsph1 | Hsph1 | 1.95E-16 | 1.74E-14 | 1.05E+01 |
| ENSRNOG00000002820 | --- | 1.98E-16 | 1.76E-14 | 7.92E+00 |
| Trem3 | Trem3 | 2.21E-16 | 1.94E-14 | 1.58E+01 |
| Scd2 | Scd2 | 2.41E-16 | 2.11E-14 | -2.32E+00 |
| Mthfd1l | Mthfd1l | 2.51E-16 | 2.18E-14 | 2.56E+00 |
| B4galt5 | B4galt5 | 2.71E-16 | 2.33E-14 | 3.72E+00 |
| Pprc1 | Pprc1 | 3.03E-16 | 2.59E-14 | 2.71E+00 |
| Ypel2 | Ypel2 | 3.09E-16 | 2.62E-14 | -2.15E+00 |
| Csf3r | Csf3r | 3.35E-16 | 2.83E-14 | 1.03E+01 |
| Cxcl1 | Cxcl1 | 3.64E-16 | 3.04E-14 | 4.49E+01 |
| Mamstr | Mamstr | 3.69E-16 | 3.07E-14 | -2.53E+00 |
| Slc7a11 | Slc7a11 | 3.73E-16 | 3.08E-14 | 1.92E+01 |
| Mxd1 | Mxd1 | 3.81E-16 | 3.13E-14 | 4.16E+00 |
| Pstpip1 | Pstpip1 | 4.11E-16 | 3.36E-14 | 3.13E+00 |
| Arl5c | Arl5c | 6.37E-16 | 5.17E-14 | 8.39E+00 |
| Whamm | Whamm | 6.77E-16 | 5.47E-14 | 2.33E+00 |
| Pvr | Pvr | 7.39E-16 | 5.91E-14 | 7.96E+00 |
| Tent5b | Tent5b | 7.96E-16 | 6.33E-14 | 3.29E+00 |
| Pgf | Pgf | 8.10E-16 | 6.41E-14 | 6.21E+00 |
| Camta1 | Camta1 | 8.89E-16 | 7.00E-14 | 2.18E+00 |
| Kcnt2 | Kcnt2 | 9.57E-16 | 7.45E-14 | -4.76E+00 |
| Meox1 | Meox1 | 1.13E-15 | 8.68E-14 | 3.50E+00 |
| Ripk3 | Ripk3 | 1.15E-15 | 8.81E-14 | 3.72E+00 |
| Lmod2 | Lmod2 | 1.16E-15 | 8.84E-14 | 2.06E+00 |
| Sema6b | Sema6b | 1.21E-15 | 9.13E-14 | 2.77E+00 |
| Eif4ebp1 | Eif4ebp1 | 1.46E-15 | 1.09E-13 | 2.23E+00 |
| C1rl | C1rl | 1.59E-15 | 1.18E-13 | 2.44E+00 |
| Gnl3 | Gnl3 | 1.72E-15 | 1.26E-13 | 2.52E+00 |
| Bcl6 | Bcl6 | 1.86E-15 | 1.36E-13 | 3.00E+00 |
| Slc35f1 | Slc35f1 | 1.92E-15 | 1.38E-13 | -2.56E+00 |
| Tmem140 | Tmem140 | 1.95E-15 | 1.39E-13 | 2.90E+00 |
| Ppp1r10 | Ppp1r10 | 1.95E-15 | 1.39E-13 | 2.12E+00 |
| Hsp90aa1 | Hsp90aa1 | 2.05E-15 | 1.44E-13 | 6.29E+00 |
| Fzd2 | Fzd2 | 2.05E-15 | 1.44E-13 | -2.64E+00 |
| RGD1563072 | RGD1563072 | 2.07E-15 | 1.45E-13 | -2.41E+00 |
| Plekhg6 | Plekhg6 | 2.68E-15 | 1.85E-13 | 2.39E+00 |
| Plaur | Plaur | 2.75E-15 | 1.88E-13 | 1.22E+01 |
| Mphosph10 | Mphosph10 | 2.79E-15 | 1.90E-13 | 2.23E+00 |
| Trib1 | Trib1 | 2.89E-15 | 1.93E-13 | 4.48E+00 |
| Mnda | Mnda | 2.89E-15 | 1.93E-13 | 5.81E+00 |
| Polr1g | Polr1g | 3.21E-15 | 2.13E-13 | 4.61E+00 |
| Litaf | Litaf | 3.21E-15 | 2.13E-13 | 3.49E+00 |
| Dipk2b | Dipk2b | 3.36E-15 | 2.21E-13 | -2.63E+00 |
| Abhd2 | Abhd2 | 3.40E-15 | 2.23E-13 | 2.67E+00 |
| Icam1 | Icam1 | 4.26E-15 | 2.76E-13 | 7.85E+00 |
| Sgms2 | Sgms2 | 4.25E-15 | 2.76E-13 | 1.23E+01 |
| Ky | Ky | 4.54E-15 | 2.93E-13 | -3.51E+00 |
| Txnrd1 | Txnrd1 | 5.32E-15 | 3.39E-13 | 2.80E+00 |
| Zbtb16 | Zbtb16 | 6.09E-15 | 3.85E-13 | 2.71E+00 |
| Scimp | Scimp | 6.10E-15 | 3.85E-13 | 5.84E+00 |
| ENSRNOG00000045654 | --- | 6.94E-15 | 4.33E-13 | 4.19E+02 |
| Ebi3 | Ebi3 | 7.14E-15 | 4.42E-13 | 8.47E+00 |
| Dnaja1 | Dnaja1 | 7.27E-15 | 4.49E-13 | 5.98E+00 |
| Ceacam4 | Ceacam4 | 7.61E-15 | 4.67E-13 | 1.25E+01 |
| Ddx21 | Ddx21 | 7.69E-15 | 4.71E-13 | 2.55E+00 |
| Ifitm1 | Ifitm1 | 7.92E-15 | 4.83E-13 | 2.95E+00 |
| Hck | Hck | 8.02E-15 | 4.87E-13 | 3.35E+00 |
| Steap4 | Steap4 | 8.14E-15 | 4.92E-13 | 2.60E+00 |
| Hcar2 | Hcar2 | 8.45E-15 | 5.09E-13 | 4.45E+01 |
| Tec | Tec | 8.65E-15 | 5.17E-13 | 2.14E+00 |
| Angptl4 | Angptl4 | 8.75E-15 | 5.20E-13 | 2.76E+01 |
| Ctsz | Ctsz | 9.95E-15 | 5.88E-13 | 2.33E+00 |
| Ptpn2 | Ptpn2 | 1.07E-14 | 6.30E-13 | 2.25E+00 |
| Mafk | Mafk | 1.12E-14 | 6.57E-13 | 2.90E+00 |
| ENSRNOG00000067170 | --- | 1.14E-14 | 6.65E-13 | -2.72E+00 |
| ENSRNOG00000067729 | --- | 1.16E-14 | 6.70E-13 | 7.25E+00 |
| Uap1 | Uap1 | 1.25E-14 | 7.23E-13 | 3.77E+00 |
| Hbegf | Hbegf | 1.30E-14 | 7.49E-13 | 1.78E+01 |
| Fjx1 | Fjx1 | 1.37E-14 | 7.84E-13 | 7.07E+00 |
| RGD1564664 | RGD1564664 | 1.40E-14 | 8.01E-13 | 4.42E+00 |
| Lilrb3 | Lilrb3 | 1.50E-14 | 8.47E-13 | -9.99E+00 |
| B3gnt7 | B3gnt7 | 1.49E-14 | 8.47E-13 | -2.19E+00 |
| Rab44 | Rab44 | 1.58E-14 | 8.90E-13 | 1.08E+01 |
| Il1b | Il1b | 1.59E-14 | 8.93E-13 | 1.40E+01 |
| Fam241b | Fam241b | 1.79E-14 | 1.00E-12 | -3.20E+00 |
| Cyb561 | Cyb561 | 1.96E-14 | 1.09E-12 | 2.47E+00 |
| Ptpn1 | Ptpn1 | 2.09E-14 | 1.15E-12 | 2.53E+00 |
| Gadd45b | Gadd45b | 2.20E-14 | 1.21E-12 | 5.28E+00 |
| Itga2 | Itga2 | 2.37E-14 | 1.29E-12 | 3.57E+00 |
| Kcnj12 | Kcnj12 | 2.42E-14 | 1.31E-12 | -2.39E+00 |
| Il4r | Il4r | 2.74E-14 | 1.49E-12 | 2.59E+00 |
| Il1r1 | Il1r1 | 2.90E-14 | 1.54E-12 | 2.18E+00 |
| Klhdc7a | Klhdc7a | 3.02E-14 | 1.60E-12 | -3.47E+00 |
| Rhou | Rhou | 3.13E-14 | 1.65E-12 | 2.84E+00 |
| Mcl1 | Mcl1 | 3.14E-14 | 1.65E-12 | 2.77E+00 |
| Prkcz | Prkcz | 3.40E-14 | 1.78E-12 | -2.55E+00 |
| ENSRNOG00000066951 | --- | 3.61E-14 | 1.88E-12 | 2.85E+00 |
| Spsb1 | Spsb1 | 3.78E-14 | 1.96E-12 | 3.13E+00 |
| Ccl2 | Ccl2 | 4.43E-14 | 2.28E-12 | 2.25E+01 |
| Xirp1 | Xirp1 | 5.30E-14 | 2.70E-12 | 1.04E+01 |
| Sell | Sell | 5.70E-14 | 2.88E-12 | 6.50E+00 |
| Mgam | Mgam | 6.56E-14 | 3.29E-12 | 1.66E+01 |
| Gclc | Gclc | 7.11E-14 | 3.56E-12 | 2.41E+00 |
| AABR07012054.1 | AABR07012054.1 | 7.19E-14 | 3.59E-12 | -2.04E+00 |
| Nppb | Nppb | 7.43E-14 | 3.69E-12 | 3.69E+00 |
| Resf1 | Resf1 | 7.75E-14 | 3.83E-12 | 2.31E+00 |
| Spred3 | Spred3 | 7.75E-14 | 3.83E-12 | 3.94E+00 |
| Wdr43 | Wdr43 | 7.78E-14 | 3.83E-12 | 2.57E+00 |
| Tspan17 | Tspan17 | 8.93E-14 | 4.35E-12 | -2.82E+00 |
| Smim5 | Smim5 | 9.14E-14 | 4.44E-12 | -2.61E+00 |
| Fcgr2a | Fcgr2a | 9.74E-14 | 4.72E-12 | 3.16E+00 |
| Kbtbd8 | Kbtbd8 | 1.10E-13 | 5.31E-12 | 2.32E+00 |
| Klra2 | Klra2 | 1.15E-13 | 5.53E-12 | -2.75E+00 |
| ENSRNOG00000026336 | --- | 1.24E-13 | 5.93E-12 | -2.32E+00 |
| Batf | Batf | 1.27E-13 | 6.05E-12 | 6.37E+00 |
| Cebpz | Cebpz | 1.29E-13 | 6.13E-12 | 2.05E+00 |
| Atf4 | Atf4 | 1.35E-13 | 6.37E-12 | 2.86E+00 |
| Nudt6 | Nudt6 | 1.41E-13 | 6.64E-12 | 2.12E+00 |
| Dgat2 | Dgat2 | 1.43E-13 | 6.71E-12 | 4.01E+00 |
| Kank1 | Kank1 | 1.58E-13 | 7.38E-12 | 2.25E+00 |
| Lyz2 | Lyz2 | 1.76E-13 | 8.13E-12 | 3.17E+00 |
| Osm | Osm | 1.80E-13 | 8.29E-12 | 1.84E+01 |
| Mybbp1a | Mybbp1a | 1.98E-13 | 9.09E-12 | 2.01E+00 |
| Gpat3 | Gpat3 | 2.18E-13 | 9.97E-12 | 6.11E+00 |
| Heatr1 | Heatr1 | 2.43E-13 | 1.10E-11 | 2.68E+00 |
| Il2rg | Il2rg | 2.56E-13 | 1.16E-11 | 2.20E+00 |
| Cyp4f18 | Cyp4f18 | 3.06E-13 | 1.37E-11 | 9.61E+00 |
| Cdr2 | Cdr2 | 3.15E-13 | 1.41E-11 | 2.85E+00 |
| Rel | Rel | 3.18E-13 | 1.41E-11 | 2.59E+00 |
| Rai14 | Rai14 | 3.34E-13 | 1.48E-11 | 2.66E+00 |
| Fosl2 | Fosl2 | 3.92E-13 | 1.72E-11 | 3.75E+00 |
| Tlr2 | Tlr2 | 4.23E-13 | 1.84E-11 | 4.12E+00 |
| Pygl | Pygl | 4.22E-13 | 1.84E-11 | 4.98E+00 |
| Ftsj3 | Ftsj3 | 4.33E-13 | 1.88E-11 | 2.16E+00 |
| Iqsec3 | Iqsec3 | 4.36E-13 | 1.88E-11 | -2.37E+00 |
| Plek | Plek | 4.61E-13 | 1.97E-11 | 3.97E+00 |
| Cp | Cp | 4.70E-13 | 2.00E-11 | 3.25E+00 |
| Nabp1 | Nabp1 | 4.94E-13 | 2.10E-11 | 2.68E+00 |
| Ch25h | Ch25h | 5.11E-13 | 2.15E-11 | 5.15E+00 |
| Sh2b2 | Sh2b2 | 5.12E-13 | 2.15E-11 | 2.63E+00 |
| Col7a1 | Col7a1 | 5.31E-13 | 2.21E-11 | 3.10E+00 |
| Cflar | Cflar | 5.33E-13 | 2.21E-11 | 2.53E+00 |
| Ifit1bl | Ifit1bl | 5.38E-13 | 2.22E-11 | 7.32E+00 |
| Adamts4 | Adamts4 | 5.37E-13 | 2.22E-11 | 1.03E+01 |
| Tnip2 | Tnip2 | 6.92E-13 | 2.82E-11 | 2.09E+00 |
| Hspb1 | Hspb1 | 7.28E-13 | 2.96E-11 | 7.30E+00 |
| Adamts1 | Adamts1 | 7.54E-13 | 3.05E-11 | 4.78E+00 |
| Jak2 | Jak2 | 7.56E-13 | 3.05E-11 | 3.74E+00 |
| ENSRNOG00000015491 | --- | 7.78E-13 | 3.12E-11 | 2.87E+00 |
| Otud1 | Otud1 | 7.84E-13 | 3.14E-11 | 1.06E+01 |
| Lrg1 | Lrg1 | 8.72E-13 | 3.47E-11 | 2.77E+00 |
| Nop58 | Nop58 | 1.05E-12 | 4.14E-11 | 2.94E+00 |
| Mmp28 | Mmp28 | 1.08E-12 | 4.27E-11 | -2.36E+00 |
| Sphk1 | Sphk1 | 1.11E-12 | 4.34E-11 | 1.43E+01 |
| Adam8 | Adam8 | 1.17E-12 | 4.57E-11 | 8.22E+00 |
| Klhl3 | Klhl3 | 1.18E-12 | 4.62E-11 | -2.26E+00 |
| Nop2 | Nop2 | 1.23E-12 | 4.79E-11 | 2.11E+00 |
| Rrp9 | Rrp9 | 1.32E-12 | 5.10E-11 | 3.13E+00 |
| Tmem171 | Tmem171 | 1.42E-12 | 5.47E-11 | -2.30E+00 |
| Il17ra | Il17ra | 1.42E-12 | 5.47E-11 | 2.63E+00 |
| Pglyrp1 | Pglyrp1 | 1.70E-12 | 6.50E-11 | 1.55E+01 |
| Dhcr7 | Dhcr7 | 1.70E-12 | 6.50E-11 | -2.12E+00 |
| Gar1 | Gar1 | 1.73E-12 | 6.58E-11 | 2.79E+00 |
| Pla2g15 | Pla2g15 | 1.83E-12 | 6.92E-11 | -2.17E+00 |
| Ccdc168 | Ccdc168 | 1.94E-12 | 7.32E-11 | 7.31E+00 |
| Tgif1 | Tgif1 | 2.04E-12 | 7.70E-11 | 3.85E+00 |
| Ptgs2 | Ptgs2 | 2.17E-12 | 8.13E-11 | 1.01E+01 |
| Pawr | Pawr | 2.22E-12 | 8.27E-11 | 3.59E+00 |
| Bid | Bid | 2.22E-12 | 8.27E-11 | 3.08E+00 |
| Rhobtb1 | Rhobtb1 | 2.33E-12 | 8.67E-11 | 3.12E+00 |
| Gpatch4 | Gpatch4 | 2.37E-12 | 8.79E-11 | 2.93E+00 |
| Nolc1 | Nolc1 | 2.51E-12 | 9.24E-11 | 2.13E+00 |
| Arid5a | Arid5a | 2.62E-12 | 9.58E-11 | 3.25E+00 |
| Il1rap | Il1rap | 2.70E-12 | 9.85E-11 | 2.30E+00 |
| Prox1 | Prox1 | 2.81E-12 | 1.02E-10 | -2.46E+00 |
| Tuba1c | Tuba1c | 2.82E-12 | 1.02E-10 | 3.80E+00 |
| Zfp281 | Zfp281 | 2.85E-12 | 1.03E-10 | 2.65E+00 |
| Osgin1 | Osgin1 | 3.03E-12 | 1.09E-10 | 3.20E+00 |
| Ms4a6a | Ms4a6a | 3.03E-12 | 1.09E-10 | 2.59E+00 |
| Mybph | Mybph | 3.12E-12 | 1.11E-10 | 1.07E+01 |
| Cnksr3 | Cnksr3 | 3.23E-12 | 1.15E-10 | 2.33E+00 |
| Utp20 | Utp20 | 3.33E-12 | 1.18E-10 | 2.05E+00 |
| Taf4b | Taf4b | 3.44E-12 | 1.22E-10 | 2.21E+00 |
| Irak2 | Irak2 | 3.46E-12 | 1.22E-10 | 3.65E+00 |
| Col27a1 | Col27a1 | 3.55E-12 | 1.25E-10 | 2.26E+00 |
| Aen | Aen | 3.70E-12 | 1.29E-10 | 3.00E+00 |
| Ccl7 | Ccl7 | 3.93E-12 | 1.37E-10 | 1.06E+01 |
| Qtrt2 | Qtrt2 | 3.94E-12 | 1.37E-10 | 2.64E+00 |
| ENSRNOG00000003809 | --- | 4.00E-12 | 1.39E-10 | 3.33E+00 |
| Aatf | Aatf | 4.25E-12 | 1.47E-10 | 2.12E+00 |
| Lrrd1 | Lrrd1 | 4.49E-12 | 1.55E-10 | 2.85E+00 |
| Sbk1 | Sbk1 | 4.55E-12 | 1.57E-10 | -2.22E+00 |
| Itgb4 | Itgb4 | 4.78E-12 | 1.64E-10 | -2.00E+00 |
| Kcna4 | Kcna4 | 4.98E-12 | 1.71E-10 | -2.06E+00 |
| Gpr176 | Gpr176 | 5.49E-12 | 1.87E-10 | 4.14E+00 |
| Rcor2 | Rcor2 | 5.73E-12 | 1.95E-10 | -2.46E+00 |
| Fam189a2 | Fam189a2 | 5.75E-12 | 1.95E-10 | 5.22E+00 |
| Irx4 | Irx4 | 6.02E-12 | 2.03E-10 | -2.23E+00 |
| Mfsd4b | Mfsd4b | 6.11E-12 | 2.06E-10 | -2.33E+00 |
| Nop14 | Nop14 | 6.27E-12 | 2.10E-10 | 2.03E+00 |
| Itga3 | Itga3 | 7.03E-12 | 2.33E-10 | 2.43E+00 |
| Tgfb2 | Tgfb2 | 7.06E-12 | 2.34E-10 | 2.50E+00 |
| Prrg4 | Prrg4 | 7.13E-12 | 2.35E-10 | 2.43E+00 |
| Zc3h12a | Zc3h12a | 7.22E-12 | 2.38E-10 | 3.79E+00 |
| Per2 | Per2 | 8.20E-12 | 2.69E-10 | 2.58E+00 |
| RGD1307182 | RGD1307182 | 8.28E-12 | 2.71E-10 | 6.18E+00 |
| Pus7 | Pus7 | 8.50E-12 | 2.77E-10 | 2.02E+00 |
| ENSRNOG00000067892 | --- | 9.22E-12 | 2.98E-10 | 2.61E+00 |
| Dmtn | Dmtn | 9.47E-12 | 3.06E-10 | -2.66E+00 |
| RGD1309534 | RGD1309534 | 9.92E-12 | 3.20E-10 | -2.17E+00 |
| Nrip3 | Nrip3 | 1.07E-11 | 3.45E-10 | 2.90E+00 |
| Flnc | Flnc | 1.08E-11 | 3.47E-10 | 7.11E+00 |
| Abra | Abra | 1.11E-11 | 3.55E-10 | 6.88E+00 |
| Cyba | Cyba | 1.12E-11 | 3.57E-10 | 2.01E+00 |
| Gnat1 | Gnat1 | 1.18E-11 | 3.74E-10 | 9.16E+00 |
| Tmem63c | Tmem63c | 1.22E-11 | 3.84E-10 | 4.05E+00 |
| Zyx | Zyx | 1.25E-11 | 3.93E-10 | 2.15E+00 |
| Ppp2r2b | Ppp2r2b | 1.39E-11 | 4.36E-10 | -3.48E+00 |
| Srm | Srm | 1.41E-11 | 4.40E-10 | 2.34E+00 |
| Bag3 | Bag3 | 1.47E-11 | 4.59E-10 | 2.67E+00 |
| ENSRNOG00000011094 | --- | 1.48E-11 | 4.59E-10 | 2.73E+00 |
| Atf3 | Atf3 | 1.52E-11 | 4.70E-10 | 1.42E+01 |
| Exo5 | Exo5 | 1.62E-11 | 4.98E-10 | 2.16E+00 |
| Susd2 | Susd2 | 1.65E-11 | 5.04E-10 | -2.18E+00 |
| Arid5b | Arid5b | 1.68E-11 | 5.12E-10 | 2.58E+00 |
| Trib3 | Trib3 | 1.69E-11 | 5.12E-10 | 6.04E+00 |
| Slc7a1 | Slc7a1 | 1.77E-11 | 5.36E-10 | 3.48E+00 |
| Rrp12 | Rrp12 | 1.81E-11 | 5.45E-10 | 3.01E+00 |
| Ppan | Ppan | 1.81E-11 | 5.45E-10 | 2.65E+00 |
| Lgals3 | Lgals3 | 1.92E-11 | 5.74E-10 | 2.42E+00 |
| Nlrp3 | Nlrp3 | 1.99E-11 | 5.93E-10 | 3.58E+00 |
| Rbm47 | Rbm47 | 2.14E-11 | 6.32E-10 | 3.38E+00 |
| Tnfrsf22 | Tnfrsf22 | 2.18E-11 | 6.44E-10 | 2.75E+00 |
| ENSRNOG00000062084 | --- | 2.21E-11 | 6.50E-10 | 1.31E+01 |
| Hivep2 | Hivep2 | 2.23E-11 | 6.55E-10 | 2.54E+00 |
| Zfp593 | Zfp593 | 2.26E-11 | 6.63E-10 | 2.73E+00 |
| Ncf2 | Ncf2 | 2.33E-11 | 6.80E-10 | 3.67E+00 |
| Irak3 | Irak3 | 2.37E-11 | 6.88E-10 | 2.66E+00 |
| Met | Met | 2.40E-11 | 6.97E-10 | 3.22E+00 |
| Ccl12 | Ccl12 | 2.44E-11 | 7.05E-10 | 7.74E+00 |
| Ston2 | Ston2 | 2.45E-11 | 7.07E-10 | -2.11E+00 |
| Aif1l | Aif1l | 2.55E-11 | 7.34E-10 | -2.23E+00 |
| ENSRNOG00000065587 | --- | 2.59E-11 | 7.43E-10 | 3.77E+00 |
| Slc2a1 | Slc2a1 | 2.63E-11 | 7.54E-10 | 2.31E+00 |
| Stx11 | Stx11 | 2.79E-11 | 7.92E-10 | 2.88E+00 |
| Il2ra | Il2ra | 2.83E-11 | 8.02E-10 | 4.02E+00 |
| Vash2 | Vash2 | 2.93E-11 | 8.26E-10 | -2.18E+00 |
| Mdn1 | Mdn1 | 3.22E-11 | 9.02E-10 | 2.37E+00 |
| Itpkc | Itpkc | 3.24E-11 | 9.08E-10 | 3.22E+00 |
| F2rl2 | F2rl2 | 3.27E-11 | 9.12E-10 | 5.17E+00 |
| Tfec | Tfec | 3.48E-11 | 9.72E-10 | 3.27E+00 |
| Ccdc71l | Ccdc71l | 3.67E-11 | 1.02E-09 | 5.06E+00 |
| N4bp2l1 | N4bp2l1 | 3.73E-11 | 1.03E-09 | 2.78E+00 |
| Noct | Noct | 4.01E-11 | 1.10E-09 | 5.02E+00 |
| ENSRNOG00000070558 | --- | 4.16E-11 | 1.13E-09 | 8.42E+00 |
| Aoah | Aoah | 4.23E-11 | 1.15E-09 | 2.62E+00 |
| Ankrd34a | Ankrd34a | 4.59E-11 | 1.24E-09 | -2.99E+00 |
| Nol8 | Nol8 | 4.58E-11 | 1.24E-09 | 2.27E+00 |
| Treml2 | Treml2 | 4.64E-11 | 1.24E-09 | 4.99E+00 |
| Slit1 | Slit1 | 4.77E-11 | 1.28E-09 | 4.80E+00 |
| Asb10 | Asb10 | 4.89E-11 | 1.31E-09 | -2.33E+00 |
| Rell1 | Rell1 | 5.50E-11 | 1.45E-09 | 2.36E+00 |
| Wnt5a | Wnt5a | 5.77E-11 | 1.52E-09 | -2.04E+00 |
| Lmcd1 | Lmcd1 | 6.18E-11 | 1.62E-09 | 7.00E+00 |
| Glis3 | Glis3 | 6.32E-11 | 1.66E-09 | 3.11E+00 |
| Chic2 | Chic2 | 6.60E-11 | 1.73E-09 | 2.06E+00 |
| RGD1307461 | RGD1307461 | 6.82E-11 | 1.77E-09 | -2.52E+00 |
| Bmp2 | Bmp2 | 6.81E-11 | 1.77E-09 | 2.55E+00 |
| Ugdh | Ugdh | 6.83E-11 | 1.77E-09 | 3.66E+00 |
| Ly49si2 | Ly49si2 | 6.94E-11 | 1.80E-09 | -2.43E+00 |
| Slc39a14 | Slc39a14 | 7.13E-11 | 1.84E-09 | 2.37E+00 |
| Lilra5 | Lilra5 | 7.30E-11 | 1.88E-09 | -3.07E+00 |
| Dusp26 | Dusp26 | 7.33E-11 | 1.88E-09 | 3.06E+00 |
| Doc2b | Doc2b | 7.59E-11 | 1.94E-09 | -4.45E+00 |
| Birc3 | Birc3 | 8.01E-11 | 2.04E-09 | 6.16E+00 |
| Rassf10 | Rassf10 | 8.24E-11 | 2.09E-09 | -3.50E+00 |
| Skil | Skil | 8.34E-11 | 2.11E-09 | 2.05E+00 |
| St8sia6 | St8sia6 | 8.40E-11 | 2.12E-09 | -2.70E+00 |
| Slc25a25 | Slc25a25 | 8.47E-11 | 2.13E-09 | 3.47E+00 |
| Cemip2 | Cemip2 | 8.56E-11 | 2.15E-09 | 3.37E+00 |
| Yod1 | Yod1 | 8.91E-11 | 2.22E-09 | 2.79E+00 |
| Fam110c | Fam110c | 9.89E-11 | 2.45E-09 | 1.20E+01 |
| RGD1563354 | RGD1563354 | 9.91E-11 | 2.45E-09 | -2.51E+00 |
| Lilrb4 | Lilrb4 | 1.02E-10 | 2.52E-09 | 4.30E+00 |
| Enc1 | Enc1 | 1.02E-10 | 2.52E-09 | 3.08E+00 |
| Lrrc3b | Lrrc3b | 1.06E-10 | 2.61E-09 | -2.27E+00 |
| Hey2 | Hey2 | 1.12E-10 | 2.74E-09 | -2.03E+00 |
| Scn3a | Scn3a | 1.13E-10 | 2.75E-09 | 2.07E+00 |
| Mmp15 | Mmp15 | 1.20E-10 | 2.89E-09 | -2.39E+00 |
| Fgr | Fgr | 1.21E-10 | 2.92E-09 | 2.92E+00 |
| Nr6a1 | Nr6a1 | 1.35E-10 | 3.22E-09 | 2.98E+00 |
| Rspo1 | Rspo1 | 1.37E-10 | 3.27E-09 | -3.24E+00 |
| Armcx6 | Armcx6 | 1.39E-10 | 3.29E-09 | -2.05E+00 |
| Yars1 | Yars1 | 1.39E-10 | 3.30E-09 | 2.44E+00 |
| ENSRNOG00000068391 | --- | 1.41E-10 | 3.32E-09 | 9.44E+00 |
| Rimbp2 | Rimbp2 | 1.43E-10 | 3.37E-09 | -2.23E+00 |
| Pik3r5 | Pik3r5 | 1.44E-10 | 3.39E-09 | 2.70E+00 |
| Dnah17 | Dnah17 | 1.47E-10 | 3.45E-09 | 3.57E+00 |
| Ccnl1 | Ccnl1 | 1.47E-10 | 3.45E-09 | 2.38E+00 |
| Tcap | Tcap | 1.52E-10 | 3.56E-09 | 2.33E+00 |
| Tars1 | Tars1 | 1.56E-10 | 3.64E-09 | 2.15E+00 |
| Slc9a3 | Slc9a3 | 1.68E-10 | 3.92E-09 | 3.30E+00 |
| Npy | Npy | 1.75E-10 | 4.06E-09 | 1.15E+01 |
| Nopchap1 | Nopchap1 | 1.80E-10 | 4.15E-09 | 2.29E+00 |
| Wdr46 | Wdr46 | 1.83E-10 | 4.21E-09 | 2.20E+00 |
| Bach1 | Bach1 | 1.86E-10 | 4.28E-09 | 2.13E+00 |
| Ippk | Ippk | 2.00E-10 | 4.57E-09 | 2.87E+00 |
| ENSRNOG00000063812 | --- | 2.04E-10 | 4.64E-09 | 5.00E+00 |
| ENSRNOG00000068731 | --- | 2.04E-10 | 4.64E-09 | 2.90E+00 |
| Tspan5 | Tspan5 | 2.08E-10 | 4.71E-09 | 2.04E+00 |
| Kbtbd3 | Kbtbd3 | 2.10E-10 | 4.76E-09 | -2.11E+00 |
| Nepro | Nepro | 2.12E-10 | 4.79E-09 | 2.37E+00 |
| Il18 | Il18 | 2.28E-10 | 5.12E-09 | 2.54E+00 |
| Uspl1 | Uspl1 | 2.30E-10 | 5.15E-09 | 2.39E+00 |
| Cdin1 | Cdin1 | 2.77E-10 | 6.15E-09 | -2.03E+00 |
| Ccn2 | Ccn2 | 2.87E-10 | 6.36E-09 | 6.45E+00 |
| Dnajb5 | Dnajb5 | 2.87E-10 | 6.36E-09 | 2.67E+00 |
| Spire1 | Spire1 | 2.95E-10 | 6.49E-09 | 2.29E+00 |
| Polr1e | Polr1e | 3.02E-10 | 6.63E-09 | 2.27E+00 |
| Nab2 | Nab2 | 3.23E-10 | 7.06E-09 | 2.19E+00 |
| Glipr2 | Glipr2 | 3.36E-10 | 7.32E-09 | 2.14E+00 |
| Spi1 | Spi1 | 3.42E-10 | 7.43E-09 | 2.23E+00 |
| Tnfaip6 | Tnfaip6 | 3.72E-10 | 8.05E-09 | 8.28E+00 |
| Hivep3 | Hivep3 | 3.77E-10 | 8.15E-09 | 2.75E+00 |
| Coq10b | Coq10b | 3.78E-10 | 8.16E-09 | 2.65E+00 |
| Fads1 | Fads1 | 3.93E-10 | 8.46E-09 | -2.04E+00 |
| Uck2 | Uck2 | 4.25E-10 | 9.10E-09 | 2.94E+00 |
| Cxcl2 | Cxcl2 | 4.29E-10 | 9.16E-09 | 2.87E+01 |
| Ankrd23 | Ankrd23 | 4.33E-10 | 9.21E-09 | 2.70E+00 |
| Csrnp1 | Csrnp1 | 4.33E-10 | 9.21E-09 | 5.04E+00 |
| Rbm19 | Rbm19 | 4.54E-10 | 9.63E-09 | 2.03E+00 |
| Rassf5 | Rassf5 | 4.69E-10 | 9.92E-09 | 2.21E+00 |
| Map3k8 | Map3k8 | 4.77E-10 | 1.01E-08 | 2.72E+00 |
| Cx3cr1 | Cx3cr1 | 4.82E-10 | 1.02E-08 | -2.91E+00 |
| Gask1a | Gask1a | 4.98E-10 | 1.05E-08 | -3.26E+00 |
| Ptpn12 | Ptpn12 | 5.35E-10 | 1.12E-08 | 2.10E+00 |
| Dusp27 | Dusp27 | 5.37E-10 | 1.12E-08 | 2.77E+00 |
| Pop1 | Pop1 | 5.43E-10 | 1.13E-08 | 2.64E+00 |
| Ctxnd1 | Ctxnd1 | 5.60E-10 | 1.17E-08 | -2.17E+00 |
| Arhgap20 | Arhgap20 | 6.29E-10 | 1.30E-08 | -2.77E+00 |
| Tubb6 | Tubb6 | 6.64E-10 | 1.36E-08 | 3.22E+00 |
| Htr2b | Htr2b | 6.97E-10 | 1.42E-08 | 2.08E+00 |
| Rnd3 | Rnd3 | 7.56E-10 | 1.54E-08 | 2.73E+00 |
| Cdkn1a | Cdkn1a | 7.72E-10 | 1.57E-08 | 2.42E+00 |
| ENSRNOG00000070522 | --- | 8.11E-10 | 1.64E-08 | -2.12E+00 |
| Cars | Cars | 8.33E-10 | 1.68E-08 | 2.18E+00 |
| Sh3pxd2b | Sh3pxd2b | 8.35E-10 | 1.69E-08 | 2.15E+00 |
| ENSRNOG00000009609 | --- | 8.51E-10 | 1.71E-08 | 2.10E+00 |
| Pak1ip1 | Pak1ip1 | 8.59E-10 | 1.73E-08 | 3.01E+00 |
| Ifrd1 | Ifrd1 | 8.62E-10 | 1.73E-08 | 2.66E+00 |
| Lrp8 | Lrp8 | 8.71E-10 | 1.75E-08 | 3.23E+00 |
| E2f5 | E2f5 | 9.24E-10 | 1.84E-08 | 2.62E+00 |
| ENSRNOG00000068569 | --- | 9.56E-10 | 1.89E-08 | -3.08E+00 |
| Dnajb1 | Dnajb1 | 1.01E-09 | 2.00E-08 | 6.49E+00 |
| Tpsab1 | Tpsab1 | 1.04E-09 | 2.04E-08 | 2.02E+00 |
| Hacd4 | Hacd4 | 1.08E-09 | 2.12E-08 | -2.52E+00 |
| Hk3 | Hk3 | 1.17E-09 | 2.28E-08 | 8.85E+00 |
| Rrp15 | Rrp15 | 1.19E-09 | 2.30E-08 | 2.10E+00 |
| Pappa1 | Pappa1 | 1.25E-09 | 2.41E-08 | 2.66E+00 |
| Ccl3 | Ccl3 | 1.31E-09 | 2.50E-08 | 8.86E+00 |
| ENSRNOG00000022601 | --- | 1.31E-09 | 2.51E-08 | 3.54E+00 |
| ENSRNOG00000045733 | --- | 1.33E-09 | 2.53E-08 | 2.39E+00 |
| ENSRNOG00000007014 | --- | 1.34E-09 | 2.54E-08 | 2.56E+00 |
| Nrxn2 | Nrxn2 | 1.46E-09 | 2.76E-08 | -2.35E+00 |
| Tfpi2 | Tfpi2 | 1.47E-09 | 2.77E-08 | 5.34E+00 |
| Gpr22 | Gpr22 | 1.49E-09 | 2.81E-08 | -3.22E+00 |
| ENSRNOG00000023005 | --- | 1.50E-09 | 2.82E-08 | 2.90E+00 |
| Asic1 | Asic1 | 1.51E-09 | 2.84E-08 | -2.31E+00 |
| Ubash3b | Ubash3b | 1.60E-09 | 3.00E-08 | 2.41E+00 |
| Itprip | Itprip | 1.69E-09 | 3.15E-08 | 2.67E+00 |
| Ciart | Ciart | 1.71E-09 | 3.19E-08 | 5.09E+00 |
| ENSRNOG00000065120 | --- | 1.81E-09 | 3.36E-08 | 2.58E+00 |
| Fndc8 | Fndc8 | 1.82E-09 | 3.37E-08 | 3.62E+00 |
| Tiparp | Tiparp | 1.82E-09 | 3.37E-08 | 2.49E+00 |
| F5 | F5 | 1.87E-09 | 3.45E-08 | 5.94E+00 |
| Ackr1 | Ackr1 | 1.94E-09 | 3.57E-08 | 3.10E+00 |
| Polr1f | Polr1f | 1.97E-09 | 3.61E-08 | 2.23E+00 |
| ENSRNOG00000037845 | --- | 2.20E-09 | 4.00E-08 | -2.43E+00 |
| Cers6 | Cers6 | 2.21E-09 | 4.00E-08 | 2.04E+00 |
| ENSRNOG00000068207 | --- | 2.33E-09 | 4.18E-08 | -3.39E+00 |
| Tsc22d1 | Tsc22d1 | 2.47E-09 | 4.42E-08 | 2.31E+00 |
| Chp2 | Chp2 | 2.58E-09 | 4.60E-08 | -2.35E+00 |
| Jaml | Jaml | 2.59E-09 | 4.61E-08 | 3.52E+00 |
| Cndp1 | Cndp1 | 2.63E-09 | 4.66E-08 | -2.09E+00 |
| Fgf9 | Fgf9 | 2.64E-09 | 4.68E-08 | -2.34E+00 |
| Slc2a10 | Slc2a10 | 2.72E-09 | 4.80E-08 | -2.25E+00 |
| Rnf115 | Rnf115 | 2.72E-09 | 4.80E-08 | 2.25E+00 |
| Trmt61a | Trmt61a | 2.75E-09 | 4.85E-08 | 2.53E+00 |
| Peg10 | Peg10 | 2.83E-09 | 4.98E-08 | 4.89E+00 |
| Dram1 | Dram1 | 3.01E-09 | 5.27E-08 | 2.95E+00 |
| Rhof | Rhof | 3.12E-09 | 5.45E-08 | 2.01E+00 |
| Fermt3 | Fermt3 | 3.12E-09 | 5.45E-08 | 2.05E+00 |
| Col12a1 | Col12a1 | 3.17E-09 | 5.51E-08 | 4.26E+00 |
| AC128859.3 | AC128859.3 | 3.19E-09 | 5.53E-08 | 4.94E+00 |
| Smco1 | Smco1 | 3.18E-09 | 5.53E-08 | -2.25E+00 |
| Ier3 | Ier3 | 3.53E-09 | 6.10E-08 | 3.50E+00 |
| St6galnac3 | St6galnac3 | 3.54E-09 | 6.10E-08 | -2.15E+00 |
| Cxcl10 | Cxcl10 | 3.78E-09 | 6.49E-08 | 2.92E+01 |
| Cd200r1 | Cd200r1 | 3.88E-09 | 6.64E-08 | 2.16E+00 |
| Tiam2 | Tiam2 | 4.15E-09 | 7.08E-08 | 4.54E+00 |
| Nat8f4 | Nat8f4 | 4.19E-09 | 7.15E-08 | -2.31E+00 |
| Hdc | Hdc | 4.21E-09 | 7.18E-08 | 2.79E+00 |
| Zfp13 | Zfp13 | 4.32E-09 | 7.31E-08 | -2.10E+00 |
| Fam124b | Fam124b | 4.31E-09 | 7.31E-08 | -2.37E+00 |
| Susd4 | Susd4 | 4.31E-09 | 7.31E-08 | -3.14E+00 |
| ENSRNOG00000066647 | --- | 4.55E-09 | 7.66E-08 | -2.30E+00 |
| Skida1 | Skida1 | 4.82E-09 | 8.06E-08 | -2.46E+00 |
| Nupr1l1 | Nupr1l1 | 4.84E-09 | 8.09E-08 | 3.43E+00 |
| Slc16a3 | Slc16a3 | 4.93E-09 | 8.23E-08 | 5.58E+00 |
| Mmp25 | Mmp25 | 5.13E-09 | 8.53E-08 | 2.46E+00 |
| Slc1a1 | Slc1a1 | 5.18E-09 | 8.60E-08 | 2.93E+00 |
| Hsf2 | Hsf2 | 5.18E-09 | 8.60E-08 | 2.29E+00 |
| Slpi | Slpi | 5.26E-09 | 8.70E-08 | 4.46E+00 |
| P2ry6 | P2ry6 | 5.35E-09 | 8.85E-08 | 4.47E+00 |
| Gbp2 | Gbp2 | 5.40E-09 | 8.92E-08 | 5.58E+00 |
| Fstl3 | Fstl3 | 5.78E-09 | 9.49E-08 | 3.04E+00 |
| Irs2 | Irs2 | 6.04E-09 | 9.90E-08 | 2.35E+00 |
| Slitrk6 | Slitrk6 | 6.44E-09 | 1.05E-07 | -2.75E+00 |
| Mob3a | Mob3a | 6.84E-09 | 1.11E-07 | 2.05E+00 |
| Cacng6 | Cacng6 | 7.07E-09 | 1.14E-07 | -2.06E+00 |
| Myot | Myot | 7.18E-09 | 1.15E-07 | 2.57E+00 |
| Vcam1 | Vcam1 | 7.33E-09 | 1.18E-07 | 6.02E+00 |
| NEWGENE_2724 | NEWGENE_2724 | 7.83E-09 | 1.25E-07 | 7.42E+00 |
| Hcls1 | Hcls1 | 7.99E-09 | 1.27E-07 | 2.06E+00 |
| Rpf2 | Rpf2 | 8.51E-09 | 1.34E-07 | 2.11E+00 |
| Cacybp | Cacybp | 8.77E-09 | 1.38E-07 | 2.54E+00 |
| ENSRNOG00000068660 | --- | 9.68E-09 | 1.50E-07 | -2.99E+00 |
| Fam181b | Fam181b | 1.00E-08 | 1.55E-07 | -2.49E+00 |
| Hspa1l | Hspa1l | 1.01E-08 | 1.57E-07 | 3.69E+00 |
| Klhl40 | Klhl40 | 1.01E-08 | 1.57E-07 | 1.00E+01 |
| Sv2a | Sv2a | 1.02E-08 | 1.57E-07 | -2.65E+00 |
| Sgsm1 | Sgsm1 | 1.03E-08 | 1.57E-07 | -2.81E+00 |
| Dtx1 | Dtx1 | 1.05E-08 | 1.62E-07 | -3.85E+00 |
| Clec7a | Clec7a | 1.06E-08 | 1.63E-07 | 3.49E+00 |
| Gem | Gem | 1.14E-08 | 1.73E-07 | 3.61E+00 |
| Ddit4l | Ddit4l | 1.15E-08 | 1.74E-07 | -2.41E+00 |
| Mefv | Mefv | 1.15E-08 | 1.74E-07 | 3.08E+00 |
| Trpm2 | Trpm2 | 1.19E-08 | 1.79E-07 | 2.40E+00 |
| Vcan | Vcan | 1.26E-08 | 1.89E-07 | 3.05E+00 |
| Stip1 | Stip1 | 1.26E-08 | 1.89E-07 | 2.47E+00 |
| Flrt3 | Flrt3 | 1.27E-08 | 1.90E-07 | -2.16E+00 |
| Pfkfb3 | Pfkfb3 | 1.36E-08 | 2.02E-07 | 5.07E+00 |
| ENSRNOG00000045992 | --- | 1.43E-08 | 2.11E-07 | -2.75E+00 |
| Marchf3 | Marchf3 | 1.51E-08 | 2.22E-07 | 3.28E+00 |
| ENSRNOG00000065465 | --- | 1.54E-08 | 2.26E-07 | 2.88E+00 |
| Efhd2 | Efhd2 | 1.58E-08 | 2.31E-07 | 2.00E+00 |
| Rab32 | Rab32 | 1.60E-08 | 2.33E-07 | 2.77E+00 |
| Akap2 | Akap2 | 1.66E-08 | 2.42E-07 | 2.62E+00 |
| Trerf1 | Trerf1 | 1.71E-08 | 2.48E-07 | -2.57E+00 |
| Sertad1 | Sertad1 | 1.72E-08 | 2.49E-07 | 2.46E+00 |
| Akap12 | Akap12 | 1.82E-08 | 2.62E-07 | 3.12E+00 |
| Vwa5a | Vwa5a | 1.83E-08 | 2.63E-07 | 2.54E+00 |
| ENSRNOG00000014201 | --- | 1.94E-08 | 2.77E-07 | 2.34E+00 |
| ENSRNOG00000064749 | --- | 1.98E-08 | 2.82E-07 | 2.58E+00 |
| ENSRNOG00000062956 | --- | 2.05E-08 | 2.91E-07 | 2.45E+00 |
| Hs3st5 | Hs3st5 | 2.07E-08 | 2.94E-07 | 2.22E+00 |
| Ggn | Ggn | 2.08E-08 | 2.94E-07 | 2.64E+00 |
| Bcar1 | Bcar1 | 2.08E-08 | 2.95E-07 | 2.29E+00 |
| ENSRNOG00000066332 | --- | 2.24E-08 | 3.15E-07 | 3.05E+00 |
| Erbb3 | Erbb3 | 2.25E-08 | 3.15E-07 | -2.16E+00 |
| F2rl1 | F2rl1 | 2.28E-08 | 3.20E-07 | 3.98E+00 |
| Nfkb1 | Nfkb1 | 2.33E-08 | 3.25E-07 | 2.19E+00 |
| Napsa | Napsa | 2.52E-08 | 3.48E-07 | 3.11E+00 |
| Ripor3 | Ripor3 | 2.61E-08 | 3.59E-07 | 2.78E+00 |
| Enah | Enah | 2.64E-08 | 3.62E-07 | 2.58E+00 |
| Siglec5 | Siglec5 | 2.69E-08 | 3.68E-07 | -2.58E+00 |
| Bcl2a1 | Bcl2a1 | 2.84E-08 | 3.88E-07 | 2.61E+00 |
| Grhl1 | Grhl1 | 2.95E-08 | 4.00E-07 | 2.43E+00 |
| ENSRNOG00000011132 | --- | 3.00E-08 | 4.06E-07 | -2.21E+00 |
| Slc5a3 | Slc5a3 | 3.03E-08 | 4.09E-07 | 2.59E+00 |
| Slc20a1 | Slc20a1 | 3.06E-08 | 4.13E-07 | 2.11E+00 |
| AABR07032097.1 | AABR07032097.1 | 3.11E-08 | 4.19E-07 | 3.07E+00 |
| Ldb2 | Ldb2 | 3.62E-08 | 4.78E-07 | -2.46E+00 |
| Ercc1 | Ercc1 | 3.71E-08 | 4.88E-07 | 2.08E+00 |
| Scnn1a | Scnn1a | 3.79E-08 | 4.98E-07 | 2.94E+00 |
| Hsp90b1 | Hsp90b1 | 3.80E-08 | 5.00E-07 | 2.04E+00 |
| P4ha1 | P4ha1 | 3.81E-08 | 5.00E-07 | 2.79E+00 |
| Cxxc4 | Cxxc4 | 3.86E-08 | 5.06E-07 | -2.24E+00 |
| Acsl4 | Acsl4 | 3.88E-08 | 5.08E-07 | 2.30E+00 |
| Zbed5 | Zbed5 | 3.97E-08 | 5.18E-07 | 2.68E+00 |
| Ptrh1 | Ptrh1 | 4.07E-08 | 5.30E-07 | 3.09E+00 |
| RGD1565057 | RGD1565057 | 4.10E-08 | 5.33E-07 | 3.13E+00 |
| Elfn2 | Elfn2 | 4.20E-08 | 5.45E-07 | -3.13E+00 |
| Gadd45g | Gadd45g | 4.32E-08 | 5.59E-07 | 4.94E+00 |
| Rtn4rl2 | Rtn4rl2 | 4.40E-08 | 5.69E-07 | 3.16E+00 |
| ENSRNOG00000061508 | --- | 4.45E-08 | 5.75E-07 | -2.41E+00 |
| Slc52a3 | Slc52a3 | 4.52E-08 | 5.83E-07 | -2.51E+00 |
| Samsn1 | Samsn1 | 4.79E-08 | 6.14E-07 | 4.45E+00 |
| Kcnh1 | Kcnh1 | 5.04E-08 | 6.43E-07 | 6.54E+00 |
| Tubb1 | Tubb1 | 5.12E-08 | 6.53E-07 | 6.82E+00 |
| ENSRNOG00000065893 | --- | 5.32E-08 | 6.75E-07 | 2.02E+00 |
| Cemip | Cemip | 5.52E-08 | 7.00E-07 | 3.03E+00 |
| ENSRNOG00000070904 | --- | 5.81E-08 | 7.30E-07 | 2.04E+00 |
| Igf2bp2 | Igf2bp2 | 5.81E-08 | 7.30E-07 | 2.98E+00 |
| ENSRNOG00000070418 | --- | 5.86E-08 | 7.36E-07 | 5.14E+00 |
| Actn1 | Actn1 | 5.94E-08 | 7.44E-07 | 2.20E+00 |
| Diras2 | Diras2 | 5.96E-08 | 7.45E-07 | -2.28E+00 |
| Tsc22d2 | Tsc22d2 | 6.16E-08 | 7.69E-07 | 2.66E+00 |
| Ppp1r15a | Ppp1r15a | 6.27E-08 | 7.81E-07 | 5.29E+00 |
| Slamf7 | Slamf7 | 6.47E-08 | 8.04E-07 | 2.62E+00 |
| ENSRNOG00000066441 | --- | 6.51E-08 | 8.08E-07 | -2.33E+00 |
| Pdpn | Pdpn | 6.67E-08 | 8.26E-07 | 3.19E+00 |
| ENSRNOG00000064211 | --- | 6.75E-08 | 8.35E-07 | 2.43E+00 |
| Cacng4 | Cacng4 | 6.93E-08 | 8.53E-07 | -2.22E+00 |
| Fam81a | Fam81a | 7.13E-08 | 8.77E-07 | -2.14E+00 |
| Gprc5a | Gprc5a | 7.29E-08 | 8.93E-07 | 5.22E+00 |
| Nod2 | Nod2 | 7.64E-08 | 9.34E-07 | 3.20E+00 |
| Clec4a | Clec4a | 7.66E-08 | 9.34E-07 | -3.73E+00 |
| Aldh1a3 | Aldh1a3 | 7.85E-08 | 9.57E-07 | -2.38E+00 |
| ENSRNOG00000071198 | --- | 7.91E-08 | 9.63E-07 | 4.47E+00 |
| Megf6 | Megf6 | 8.06E-08 | 9.81E-07 | -3.03E+00 |
| Spon2 | Spon2 | 8.19E-08 | 9.93E-07 | -2.51E+00 |
| ENSRNOG00000065208 | --- | 8.39E-08 | 1.01E-06 | 4.59E+00 |
| ENSRNOG00000069248 | --- | 8.41E-08 | 1.02E-06 | 3.37E+00 |
| Il31ra | Il31ra | 8.43E-08 | 1.02E-06 | 3.99E+00 |
| Tagap | Tagap | 8.53E-08 | 1.03E-06 | 2.59E+00 |
| Pim1 | Pim1 | 8.95E-08 | 1.08E-06 | 3.09E+00 |
| Kcnmb4 | Kcnmb4 | 9.01E-08 | 1.08E-06 | 2.17E+00 |
| Hpdl | Hpdl | 9.52E-08 | 1.13E-06 | -2.07E+00 |
| Rab3d | Rab3d | 9.87E-08 | 1.17E-06 | -2.09E+00 |
| Cndp2 | Cndp2 | 9.91E-08 | 1.17E-06 | 2.53E+00 |
| Marchf1 | Marchf1 | 1.05E-07 | 1.23E-06 | 2.15E+00 |
| Rgs16 | Rgs16 | 1.06E-07 | 1.24E-06 | 4.34E+00 |
| Trmt6 | Trmt6 | 1.17E-07 | 1.37E-06 | 2.10E+00 |
| ENSRNOG00000066282 | --- | 1.20E-07 | 1.39E-06 | -2.34E+00 |
| AC112568.1 | AC112568.1 | 1.21E-07 | 1.40E-06 | 1.45E+01 |
| Ddx18 | Ddx18 | 1.22E-07 | 1.41E-06 | 2.05E+00 |
| Slc41a2 | Slc41a2 | 1.23E-07 | 1.42E-06 | 2.51E+00 |
| Ano2 | Ano2 | 1.26E-07 | 1.46E-06 | -2.09E+00 |
| Clec1b | Clec1b | 1.29E-07 | 1.48E-06 | 6.24E+00 |
| Gal3st3 | Gal3st3 | 1.34E-07 | 1.54E-06 | -2.11E+00 |
| Ahsa2 | Ahsa2 | 1.35E-07 | 1.54E-06 | 2.40E+00 |
| Cdkn2b | Cdkn2b | 1.40E-07 | 1.59E-06 | 4.91E+00 |
| Capn6 | Capn6 | 1.41E-07 | 1.61E-06 | -3.09E+00 |
| Rcan1 | Rcan1 | 1.47E-07 | 1.67E-06 | 2.52E+00 |
| AABR07026032.1 | AABR07026032.1 | 1.57E-07 | 1.77E-06 | -2.16E+00 |
| Relt | Relt | 1.69E-07 | 1.90E-06 | 2.30E+00 |
| Sp140 | Sp140 | 1.73E-07 | 1.94E-06 | 2.25E+00 |
| Tmem198b | Tmem198b | 1.80E-07 | 2.01E-06 | -2.25E+00 |
| Kcnc2 | Kcnc2 | 1.80E-07 | 2.01E-06 | -2.98E+00 |
| Tafa2 | Tafa2 | 1.83E-07 | 2.04E-06 | 2.37E+00 |
| Kcnab1 | Kcnab1 | 1.87E-07 | 2.08E-06 | 2.15E+00 |
| Mrc1 | Mrc1 | 1.88E-07 | 2.09E-06 | 2.06E+00 |
| Ccdc92b | Ccdc92b | 1.89E-07 | 2.10E-06 | -2.16E+00 |
| Slc16a7 | Slc16a7 | 1.91E-07 | 2.11E-06 | -2.07E+00 |
| Lmo3 | Lmo3 | 1.91E-07 | 2.11E-06 | -2.37E+00 |
| Cxcl9 | Cxcl9 | 1.93E-07 | 2.13E-06 | 1.18E+01 |
| Gpr37l1 | Gpr37l1 | 1.93E-07 | 2.14E-06 | -2.16E+00 |
| Ccdc117 | Ccdc117 | 1.94E-07 | 2.15E-06 | 2.28E+00 |
| Epha2 | Epha2 | 2.00E-07 | 2.20E-06 | 3.15E+00 |
| Hunk | Hunk | 2.03E-07 | 2.22E-06 | 3.29E+00 |
| Clec5a | Clec5a | 2.11E-07 | 2.31E-06 | 2.92E+00 |
| Ptprn | Ptprn | 2.17E-07 | 2.37E-06 | 4.89E+00 |
| Cxcl12 | Cxcl12 | 2.25E-07 | 2.45E-06 | -2.03E+00 |
| Tmem215 | Tmem215 | 2.25E-07 | 2.45E-06 | -4.21E+00 |
| Smad9 | Smad9 | 2.26E-07 | 2.46E-06 | -2.54E+00 |
| ENSRNOG00000067089 | --- | 2.27E-07 | 2.47E-06 | 2.01E+00 |
| Hspa5 | Hspa5 | 2.28E-07 | 2.47E-06 | 2.29E+00 |
| Pmaip1 | Pmaip1 | 2.32E-07 | 2.52E-06 | 2.61E+00 |
| Lilrb2 | Lilrb2 | 2.35E-07 | 2.54E-06 | 4.53E+00 |
| Itga2b | Itga2b | 2.35E-07 | 2.54E-06 | 5.63E+00 |
| Qtrt1 | Qtrt1 | 2.38E-07 | 2.57E-06 | 2.29E+00 |
| Adm | Adm | 2.39E-07 | 2.58E-06 | 2.33E+00 |
| Rnf225 | Rnf225 | 2.43E-07 | 2.61E-06 | 4.57E+00 |
| Abca5 | Abca5 | 2.54E-07 | 2.72E-06 | 2.77E+00 |
| Gna13 | Gna13 | 2.70E-07 | 2.87E-06 | 2.12E+00 |
| Pnma8b | Pnma8b | 2.72E-07 | 2.89E-06 | -2.75E+00 |
| ENSRNOG00000067696 | --- | 2.80E-07 | 2.97E-06 | 3.31E+00 |
| Fxyd4 | Fxyd4 | 2.83E-07 | 3.00E-06 | 2.64E+00 |
| Wnt9a | Wnt9a | 2.86E-07 | 3.02E-06 | -2.18E+00 |
| Xcr1 | Xcr1 | 2.88E-07 | 3.05E-06 | -3.37E+00 |
| Rassf1 | Rassf1 | 2.96E-07 | 3.11E-06 | 2.20E+00 |
| ENSRNOG00000067849 | --- | 2.98E-07 | 3.14E-06 | 3.65E+00 |
| Tubb3 | Tubb3 | 3.03E-07 | 3.19E-06 | 4.48E+00 |
| Dot1l | Dot1l | 3.05E-07 | 3.21E-06 | 3.47E+00 |
| Erich6 | Erich6 | 3.08E-07 | 3.24E-06 | 2.42E+00 |
| Galnt14 | Galnt14 | 3.16E-07 | 3.30E-06 | 2.69E+00 |
| Ass1 | Ass1 | 3.23E-07 | 3.37E-06 | 3.28E+00 |
| ENSRNOG00000066184 | --- | 3.26E-07 | 3.40E-06 | -2.12E+00 |
| Reep2 | Reep2 | 3.27E-07 | 3.41E-06 | 4.26E+00 |
| Pcdh17 | Pcdh17 | 3.38E-07 | 3.51E-06 | -2.36E+00 |
| ENSRNOG00000063237 | --- | 3.44E-07 | 3.56E-06 | -3.12E+00 |
| Inka2 | Inka2 | 3.47E-07 | 3.59E-06 | -2.11E+00 |
| AABR07063082.1 | AABR07063082.1 | 3.58E-07 | 3.70E-06 | 3.35E+00 |
| Frat2 | Frat2 | 3.60E-07 | 3.72E-06 | 2.73E+00 |
| ENSRNOG00000069883 | --- | 3.61E-07 | 3.72E-06 | -2.71E+00 |
| Pard6b | Pard6b | 3.85E-07 | 3.95E-06 | 3.23E+00 |
| ENSRNOG00000064113 | --- | 3.91E-07 | 4.01E-06 | -4.62E+00 |
| Maob | Maob | 3.98E-07 | 4.07E-06 | -2.87E+00 |
| Prg4 | Prg4 | 4.06E-07 | 4.13E-06 | 2.18E+00 |
| Lzts1 | Lzts1 | 4.10E-07 | 4.17E-06 | -2.15E+00 |
| Nfe2 | Nfe2 | 4.10E-07 | 4.18E-06 | 3.27E+00 |
| Smpd3 | Smpd3 | 4.28E-07 | 4.35E-06 | 2.09E+00 |
| Nlrc5 | Nlrc5 | 4.29E-07 | 4.35E-06 | 3.31E+00 |
| AC094212.1 | AC094212.1 | 4.37E-07 | 4.42E-06 | -2.36E+00 |
| Cd44 | Cd44 | 4.39E-07 | 4.43E-06 | 2.46E+00 |
| Slamf8 | Slamf8 | 4.42E-07 | 4.45E-06 | 3.30E+00 |
| Fam167a | Fam167a | 4.45E-07 | 4.48E-06 | 3.27E+00 |
| Ltb | Ltb | 4.45E-07 | 4.48E-06 | 2.93E+00 |
| C1qtnf2 | C1qtnf2 | 4.57E-07 | 4.57E-06 | -2.02E+00 |
| Fpgs | Fpgs | 4.60E-07 | 4.60E-06 | 2.37E+00 |
| Nr4a3 | Nr4a3 | 4.64E-07 | 4.63E-06 | 4.73E+00 |
| Lif | Lif | 4.67E-07 | 4.66E-06 | 9.36E+00 |
| Trpc3 | Trpc3 | 4.67E-07 | 4.66E-06 | -2.03E+00 |
| Trpv4 | Trpv4 | 4.73E-07 | 4.71E-06 | 2.31E+00 |
| Nrk | Nrk | 4.82E-07 | 4.80E-06 | -2.00E+00 |
| ENSRNOG00000063207 | --- | 4.90E-07 | 4.87E-06 | 2.10E+00 |
| Hsf2bp | Hsf2bp | 5.02E-07 | 4.98E-06 | 5.73E+00 |
| Gpnmb | Gpnmb | 5.02E-07 | 4.98E-06 | 2.06E+00 |
| ENSRNOG00000047204 | --- | 5.11E-07 | 5.06E-06 | 6.56E+00 |
| Zfyve28 | Zfyve28 | 5.12E-07 | 5.06E-06 | -2.17E+00 |
| ENSRNOG00000000875 | --- | 5.12E-07 | 5.06E-06 | 2.55E+00 |
| Ugcg | Ugcg | 5.22E-07 | 5.15E-06 | 2.17E+00 |
| Plac8 | Plac8 | 5.33E-07 | 5.24E-06 | 2.49E+00 |
| Rrad | Rrad | 5.50E-07 | 5.40E-06 | 2.43E+00 |
| Tbx2 | Tbx2 | 5.63E-07 | 5.51E-06 | -2.11E+00 |
| Lrrc17 | Lrrc17 | 5.71E-07 | 5.57E-06 | -2.26E+00 |
| Tg | Tg | 5.74E-07 | 5.60E-06 | 3.39E+00 |
| Samd10 | Samd10 | 6.02E-07 | 5.85E-06 | -2.06E+00 |
| Fam184a | Fam184a | 6.08E-07 | 5.90E-06 | 2.72E+00 |
| Tent5a | Tent5a | 6.47E-07 | 6.24E-06 | 3.57E+00 |
| Fam163b | Fam163b | 7.14E-07 | 6.83E-06 | -2.32E+00 |
| ENSRNOG00000066444 | --- | 7.26E-07 | 6.93E-06 | 2.45E+00 |
| Sesn2 | Sesn2 | 7.30E-07 | 6.97E-06 | 2.45E+00 |
| Mybpc2 | Mybpc2 | 7.41E-07 | 7.06E-06 | 2.18E+00 |
| Fscn2 | Fscn2 | 7.57E-07 | 7.19E-06 | 3.34E+00 |
| Adamts3 | Adamts3 | 7.61E-07 | 7.22E-06 | 2.18E+00 |
| Zcchc18 | Zcchc18 | 8.09E-07 | 7.65E-06 | -3.62E+00 |
| Dok4 | Dok4 | 8.42E-07 | 7.94E-06 | -2.37E+00 |
| Cish | Cish | 9.08E-07 | 8.49E-06 | 2.31E+00 |
| Tspyl4 | Tspyl4 | 9.18E-07 | 8.57E-06 | -2.50E+00 |
| AABR07002848.1 | AABR07002848.1 | 9.30E-07 | 8.66E-06 | 2.97E+00 |
| AABR07039336.2 | AABR07039336.2 | 9.36E-07 | 8.72E-06 | -2.02E+00 |
| Cd226 | Cd226 | 9.53E-07 | 8.83E-06 | 3.68E+00 |
| ENSRNOG00000029191 | --- | 9.56E-07 | 8.86E-06 | 5.12E+00 |
| Treml1 | Treml1 | 9.65E-07 | 8.92E-06 | 5.37E+00 |
| Npr3 | Npr3 | 9.82E-07 | 9.06E-06 | -2.79E+00 |
| ENSRNOG00000065682 | --- | 9.89E-07 | 9.12E-06 | 3.82E+00 |
| Ccl24 | Ccl24 | 1.01E-06 | 9.26E-06 | -2.89E+00 |
| Plagl1 | Plagl1 | 1.02E-06 | 9.35E-06 | 2.74E+00 |
| Bmpr1b | Bmpr1b | 1.04E-06 | 9.49E-06 | 2.89E+00 |
| Vxn | Vxn | 1.07E-06 | 9.74E-06 | -2.48E+00 |
| Itgb3 | Itgb3 | 1.08E-06 | 9.81E-06 | 2.72E+00 |
| Gdf15 | Gdf15 | 1.14E-06 | 1.03E-05 | 6.70E+00 |
| Edn1 | Edn1 | 1.15E-06 | 1.04E-05 | 3.33E+00 |
| Mpig6b | Mpig6b | 1.15E-06 | 1.04E-05 | 3.60E+00 |
| ENSRNOG00000065520 | --- | 1.17E-06 | 1.05E-05 | 2.59E+00 |
| Capn13 | Capn13 | 1.18E-06 | 1.06E-05 | -2.92E+00 |
| Il7r | Il7r | 1.24E-06 | 1.11E-05 | 3.13E+00 |
| Gp9 | Gp9 | 1.25E-06 | 1.12E-05 | 6.97E+00 |
| Angpt2 | Angpt2 | 1.27E-06 | 1.13E-05 | 2.51E+00 |
| Atp8b4 | Atp8b4 | 1.28E-06 | 1.14E-05 | 2.12E+00 |
| Nfkbid | Nfkbid | 1.33E-06 | 1.18E-05 | 2.82E+00 |
| Gpr39 | Gpr39 | 1.36E-06 | 1.20E-05 | 3.12E+00 |
| Cited2 | Cited2 | 1.41E-06 | 1.24E-05 | 3.54E+00 |
| Usp53 | Usp53 | 1.43E-06 | 1.25E-05 | 2.02E+00 |
| Tll1 | Tll1 | 1.43E-06 | 1.25E-05 | 2.23E+00 |
| Fhdc1 | Fhdc1 | 1.44E-06 | 1.25E-05 | 2.04E+00 |
| Ankdd1a | Ankdd1a | 1.44E-06 | 1.26E-05 | 2.59E+00 |
| Ccdc88b | Ccdc88b | 1.46E-06 | 1.27E-05 | 2.31E+00 |
| Cd86 | Cd86 | 1.51E-06 | 1.31E-05 | 2.25E+00 |
| P2ry2 | P2ry2 | 1.52E-06 | 1.32E-05 | 2.00E+00 |
| Muc20 | Muc20 | 1.54E-06 | 1.33E-05 | 6.94E+00 |
| Pcdh20 | Pcdh20 | 1.58E-06 | 1.36E-05 | 2.55E+00 |
| ENSRNOG00000069480 | --- | 1.58E-06 | 1.36E-05 | 2.65E+00 |
| Avil | Avil | 1.59E-06 | 1.37E-05 | 2.44E+00 |
| Xirp2 | Xirp2 | 1.61E-06 | 1.38E-05 | 3.57E+00 |
| Gp1ba | Gp1ba | 1.66E-06 | 1.43E-05 | 2.29E+00 |
| ENSRNOG00000064282 | --- | 1.70E-06 | 1.45E-05 | 6.31E+00 |
| Nos2 | Nos2 | 1.79E-06 | 1.52E-05 | 5.78E+00 |
| Cdh17 | Cdh17 | 1.82E-06 | 1.55E-05 | 4.06E+00 |
| Cdk5r1 | Cdk5r1 | 1.87E-06 | 1.58E-05 | 2.13E+00 |
| Loxl4 | Loxl4 | 1.89E-06 | 1.59E-05 | 2.55E+00 |
| Jag2 | Jag2 | 1.98E-06 | 1.66E-05 | -2.34E+00 |
| Ccdc146 | Ccdc146 | 2.00E-06 | 1.68E-05 | 2.90E+00 |
| ENSRNOG00000069279 | --- | 2.10E-06 | 1.75E-05 | 3.00E+00 |
| Cbln2 | Cbln2 | 2.11E-06 | 1.76E-05 | 7.56E+00 |
| Znrf4 | Znrf4 | 2.15E-06 | 1.79E-05 | -2.95E+00 |
| Rhov | Rhov | 2.21E-06 | 1.82E-05 | 3.87E+00 |
| Ddn | Ddn | 2.21E-06 | 1.82E-05 | -2.46E+00 |
| ENSRNOG00000064941 | --- | 2.22E-06 | 1.83E-05 | -2.09E+00 |
| Piezo2 | Piezo2 | 2.23E-06 | 1.83E-05 | -2.07E+00 |
| MGC105649 | MGC105649 | 2.25E-06 | 1.85E-05 | 2.69E+00 |
| ENSRNOG00000065725 | --- | 2.27E-06 | 1.86E-05 | 3.13E+00 |
| ENSRNOG00000063974 | --- | 2.37E-06 | 1.94E-05 | 2.14E+00 |
| Zfp879 | Zfp879 | 2.37E-06 | 1.94E-05 | -2.21E+00 |
| Slc2a6 | Slc2a6 | 2.41E-06 | 1.96E-05 | 2.35E+00 |
| Lonrf3 | Lonrf3 | 2.51E-06 | 2.04E-05 | 2.43E+00 |
| ENSRNOG00000063384 | --- | 2.54E-06 | 2.06E-05 | 2.19E+00 |
| RGD1561662 | RGD1561662 | 2.59E-06 | 2.10E-05 | 2.44E+00 |
| Zfp697 | Zfp697 | 2.59E-06 | 2.10E-05 | 2.13E+00 |
| AABR07062138.2 | AABR07062138.2 | 2.62E-06 | 2.11E-05 | 2.06E+00 |
| ENSRNOG00000062648 | --- | 2.68E-06 | 2.16E-05 | 8.29E+00 |
| ENSRNOG00000070737 | --- | 2.72E-06 | 2.19E-05 | 2.82E+00 |
| ENSRNOG00000063474 | --- | 2.72E-06 | 2.19E-05 | -2.02E+00 |
| ENSRNOG00000067297 | --- | 2.81E-06 | 2.25E-05 | -2.67E+00 |
| ENSRNOG00000070695 | --- | 2.87E-06 | 2.29E-05 | 2.99E+00 |
| ENSRNOG00000002844 | --- | 2.98E-06 | 2.37E-05 | 2.02E+00 |
| Pde1c | Pde1c | 3.04E-06 | 2.42E-05 | -2.45E+00 |
| Pf4 | Pf4 | 3.23E-06 | 2.55E-05 | 4.38E+00 |
| Fgfr3 | Fgfr3 | 3.37E-06 | 2.64E-05 | -2.49E+00 |
| ENSRNOG00000063994 | --- | 3.39E-06 | 2.66E-05 | -2.84E+00 |
| Oas3 | Oas3 | 3.41E-06 | 2.67E-05 | 3.89E+00 |
| ENSRNOG00000067704 | --- | 3.52E-06 | 2.75E-05 | -2.10E+00 |
| Chst11 | Chst11 | 3.72E-06 | 2.89E-05 | 2.15E+00 |
| Klf5 | Klf5 | 3.72E-06 | 2.89E-05 | 4.53E+00 |
| Gck | Gck | 3.76E-06 | 2.91E-05 | -2.54E+00 |
| Ier5 | Ier5 | 3.79E-06 | 2.93E-05 | 2.69E+00 |
| Nfam1 | Nfam1 | 3.82E-06 | 2.95E-05 | 2.01E+00 |
| Insc | Insc | 3.83E-06 | 2.96E-05 | -2.97E+00 |
| Twist2 | Twist2 | 4.02E-06 | 3.09E-05 | 2.57E+00 |
| Kctd4 | Kctd4 | 4.12E-06 | 3.15E-05 | 2.09E+00 |
| ENSRNOG00000033256 | --- | 4.29E-06 | 3.26E-05 | 2.40E+00 |
| Cntfr | Cntfr | 4.32E-06 | 3.29E-05 | 2.68E+00 |
| Junb | Junb | 4.33E-06 | 3.29E-05 | 3.79E+00 |
| ENSRNOG00000017054 | --- | 4.72E-06 | 3.55E-05 | -2.29E+00 |
| AABR07030544.1 | AABR07030544.1 | 4.96E-06 | 3.70E-05 | 2.93E+00 |
| Csrp3 | Csrp3 | 4.96E-06 | 3.70E-05 | 2.14E+00 |
| Ngp | Ngp | 5.00E-06 | 3.73E-05 | 5.53E+00 |
| Oas1k | Oas1k | 5.16E-06 | 3.82E-05 | 2.49E+00 |
| Slc10a6 | Slc10a6 | 5.19E-06 | 3.84E-05 | 2.08E+00 |
| ENSRNOG00000070936 | --- | 5.42E-06 | 3.98E-05 | -2.04E+00 |
| ENSRNOG00000069485 | --- | 5.51E-06 | 4.04E-05 | 3.45E+00 |
| Gsg1l | Gsg1l | 5.57E-06 | 4.08E-05 | -2.11E+00 |
| Olfm2 | Olfm2 | 5.65E-06 | 4.13E-05 | 3.82E+00 |
| ENSRNOG00000063760 | --- | 5.69E-06 | 4.15E-05 | -2.52E+00 |
| Zswim4 | Zswim4 | 5.76E-06 | 4.20E-05 | 2.10E+00 |
| ENSRNOG00000070677 | --- | 6.02E-06 | 4.36E-05 | 2.70E+00 |
| ENSRNOG00000063037 | --- | 6.09E-06 | 4.40E-05 | 2.50E+00 |
| Ankrd1 | Ankrd1 | 6.17E-06 | 4.45E-05 | 2.04E+00 |
| Lamc2 | Lamc2 | 6.21E-06 | 4.47E-05 | 2.14E+00 |
| Tifa | Tifa | 6.26E-06 | 4.51E-05 | 3.96E+00 |
| Nfkbia | Nfkbia | 6.46E-06 | 4.63E-05 | 2.18E+00 |
| Exoc3l1 | Exoc3l1 | 6.62E-06 | 4.74E-05 | -2.06E+00 |
| ENSRNOG00000064615 | --- | 6.78E-06 | 4.84E-05 | -2.17E+00 |
| Ifit3 | Ifit3 | 6.87E-06 | 4.90E-05 | 5.26E+00 |
| ENSRNOG00000067344 | --- | 7.10E-06 | 5.04E-05 | 2.04E+00 |
| AABR07034648.1 | AABR07034648.1 | 7.22E-06 | 5.11E-05 | 3.77E+00 |
| Kif26a | Kif26a | 7.31E-06 | 5.17E-05 | -2.56E+00 |
| Ociad2 | Ociad2 | 7.33E-06 | 5.17E-05 | 2.58E+00 |
| R3hcc1l | R3hcc1l | 7.52E-06 | 5.29E-05 | 2.02E+00 |
| Klhl41 | Klhl41 | 7.60E-06 | 5.34E-05 | 2.29E+00 |
| Aass | Aass | 7.69E-06 | 5.40E-05 | -2.34E+00 |
| AABR07038983.1 | AABR07038983.1 | 8.25E-06 | 5.77E-05 | 2.49E+00 |
| Fam83e | Fam83e | 8.28E-06 | 5.79E-05 | 3.25E+00 |
| Azin2 | Azin2 | 8.77E-06 | 6.09E-05 | 2.23E+00 |
| Fzd9 | Fzd9 | 9.04E-06 | 6.27E-05 | 2.90E+00 |
| Tnf | Tnf | 9.11E-06 | 6.30E-05 | 2.49E+00 |
| Tmem14a | Tmem14a | 9.65E-06 | 6.63E-05 | -2.10E+00 |
| ENSRNOG00000070943 | --- | 9.93E-06 | 6.79E-05 | -2.36E+00 |
| Nrg2 | Nrg2 | 9.93E-06 | 6.79E-05 | -2.95E+00 |
| ENSRNOG00000046353 | --- | 1.09E-05 | 7.37E-05 | -2.20E+00 |
| Rnf19b | Rnf19b | 1.09E-05 | 7.41E-05 | 2.76E+00 |
| Col28a1 | Col28a1 | 1.10E-05 | 7.43E-05 | -2.48E+00 |
| ENSRNOG00000012358 | --- | 1.13E-05 | 7.66E-05 | -2.74E+00 |
| Derl3 | Derl3 | 1.16E-05 | 7.79E-05 | 2.34E+00 |
| Ripk2 | Ripk2 | 1.16E-05 | 7.83E-05 | 2.40E+00 |
| Ncoa7 | Ncoa7 | 1.19E-05 | 7.96E-05 | 2.01E+00 |
| Efna4 | Efna4 | 1.19E-05 | 7.99E-05 | -2.20E+00 |
| Gbp5 | Gbp5 | 1.20E-05 | 8.03E-05 | 5.09E+00 |
| Relb | Relb | 1.23E-05 | 8.20E-05 | 2.06E+00 |
| Sptbn4 | Sptbn4 | 1.24E-05 | 8.27E-05 | 3.58E+00 |
| Creld2 | Creld2 | 1.26E-05 | 8.41E-05 | 2.41E+00 |
| Aldh3b1 | Aldh3b1 | 1.28E-05 | 8.50E-05 | -2.01E+00 |
| Lrrn4 | Lrrn4 | 1.30E-05 | 8.63E-05 | -5.35E+00 |
| Spp1 | Spp1 | 1.33E-05 | 8.78E-05 | 6.29E+00 |
| ENSRNOG00000063319 | --- | 1.33E-05 | 8.79E-05 | 3.45E+00 |
| Plekhh3 | Plekhh3 | 1.35E-05 | 8.94E-05 | -2.01E+00 |
| Fst | Fst | 1.36E-05 | 9.01E-05 | 5.23E+00 |
| Pcsk1 | Pcsk1 | 1.39E-05 | 9.12E-05 | 3.14E+00 |
| Sfmbt2 | Sfmbt2 | 1.41E-05 | 9.29E-05 | 2.10E+00 |
| Nfkbiz | Nfkbiz | 1.45E-05 | 9.54E-05 | 3.53E+00 |
| Fscn3 | Fscn3 | 1.47E-05 | 9.63E-05 | 2.63E+00 |
| ENSRNOG00000069433 | --- | 1.50E-05 | 9.79E-05 | -2.25E+00 |
| Ism2 | Ism2 | 1.54E-05 | 1.00E-04 | 2.41E+00 |
| Hbb-bs | Hbb-bs | 1.58E-05 | 1.03E-04 | 4.86E+00 |
| Slc15a3 | Slc15a3 | 1.63E-05 | 1.05E-04 | 2.52E+00 |
| Ly49si1 | Ly49si1 | 1.64E-05 | 1.06E-04 | -2.24E+00 |
| Begain | Begain | 1.74E-05 | 1.12E-04 | 2.14E+00 |
| Plk3 | Plk3 | 1.75E-05 | 1.12E-04 | 2.23E+00 |
| Tap2 | Tap2 | 1.80E-05 | 1.15E-04 | 2.19E+00 |
| Lilrc2 | Lilrc2 | 1.80E-05 | 1.15E-04 | 3.28E+00 |
| Serpinf2 | Serpinf2 | 1.82E-05 | 1.16E-04 | 2.88E+00 |
| Mpz | Mpz | 1.82E-05 | 1.16E-04 | -3.10E+00 |
| Aplnr | Aplnr | 1.89E-05 | 1.20E-04 | -3.45E+00 |
| Tnfsf18 | Tnfsf18 | 1.95E-05 | 1.23E-04 | 6.00E+00 |
| ENSRNOG00000047004 | --- | 2.03E-05 | 1.28E-04 | 2.77E+00 |
| Ccn1 | Ccn1 | 2.03E-05 | 1.28E-04 | 5.31E+00 |
| Ermap | Ermap | 2.04E-05 | 1.29E-04 | 4.43E+00 |
| Ptgir | Ptgir | 2.12E-05 | 1.33E-04 | 2.49E+00 |
| Fam171a2 | Fam171a2 | 2.14E-05 | 1.34E-04 | -2.29E+00 |
| Rnf125 | Rnf125 | 2.21E-05 | 1.37E-04 | 2.40E+00 |
| Ffar2 | Ffar2 | 2.26E-05 | 1.40E-04 | 3.41E+00 |
| Pkhd1l1 | Pkhd1l1 | 2.28E-05 | 1.41E-04 | -2.29E+00 |
| Mapk13 | Mapk13 | 2.32E-05 | 1.43E-04 | 3.53E+00 |
| Plau | Plau | 2.32E-05 | 1.43E-04 | 2.97E+00 |
| Il15ra | Il15ra | 2.33E-05 | 1.44E-04 | 2.04E+00 |
| Zmynd15 | Zmynd15 | 2.35E-05 | 1.45E-04 | 2.50E+00 |
| Slc7a2 | Slc7a2 | 2.36E-05 | 1.45E-04 | 2.29E+00 |
| Cx3cl1 | Cx3cl1 | 2.38E-05 | 1.46E-04 | 2.19E+00 |
| Gata5 | Gata5 | 2.48E-05 | 1.52E-04 | -2.32E+00 |
| Nuak2 | Nuak2 | 2.53E-05 | 1.55E-04 | 2.14E+00 |
| Spry2 | Spry2 | 2.60E-05 | 1.58E-04 | 2.68E+00 |
| Chmp4bl1 | Chmp4bl1 | 2.66E-05 | 1.61E-04 | 3.40E+00 |
| RGD1308750 | RGD1308750 | 2.67E-05 | 1.61E-04 | 5.11E+00 |
| Psat1 | Psat1 | 2.67E-05 | 1.61E-04 | 2.71E+00 |
| Basp1 | Basp1 | 2.68E-05 | 1.62E-04 | 2.05E+00 |
| AABR07030771.1 | AABR07030771.1 | 2.69E-05 | 1.62E-04 | 3.87E+00 |
| Tnfrsf11b | Tnfrsf11b | 2.76E-05 | 1.65E-04 | 3.32E+00 |
| ENSRNOG00000064054 | --- | 2.77E-05 | 1.66E-04 | 2.06E+00 |
| Scn3b | Scn3b | 2.78E-05 | 1.66E-04 | 2.06E+00 |
| ENSRNOG00000067276 | --- | 2.82E-05 | 1.68E-04 | 2.42E+00 |
| Gcnt4 | Gcnt4 | 3.02E-05 | 1.79E-04 | 2.38E+00 |
| ENSRNOG00000070694 | --- | 3.04E-05 | 1.80E-04 | 2.39E+00 |
| ENSRNOG00000069929 | --- | 3.11E-05 | 1.84E-04 | -2.08E+00 |
| AABR07004269.3 | AABR07004269.3 | 3.20E-05 | 1.89E-04 | 2.82E+00 |
| AY172581.9 | AY172581.9 | 3.33E-05 | 1.96E-04 | 5.05E+00 |
| Trhde | Trhde | 3.34E-05 | 1.96E-04 | -2.11E+00 |
| Rem1 | Rem1 | 3.35E-05 | 1.97E-04 | -2.22E+00 |
| Gpm6a | Gpm6a | 3.37E-05 | 1.98E-04 | -2.53E+00 |
| NEWGENE_1310680 | NEWGENE_1310680 | 3.40E-05 | 1.99E-04 | -2.05E+00 |
| Fam205a | Fam205a | 3.58E-05 | 2.08E-04 | -2.93E+00 |
| Rtn4r | Rtn4r | 3.58E-05 | 2.09E-04 | -2.58E+00 |
| Arhgap8 | Arhgap8 | 3.80E-05 | 2.20E-04 | 2.47E+00 |
| Zfp865 | Zfp865 | 3.84E-05 | 2.22E-04 | -2.82E+00 |
| ENSRNOG00000062779 | --- | 3.86E-05 | 2.23E-04 | 2.35E+00 |
| ENSRNOG00000010775 | --- | 3.86E-05 | 2.23E-04 | 2.20E+00 |
| ENSRNOG00000067530 | --- | 3.88E-05 | 2.24E-04 | -2.58E+00 |
| Dedd2 | Dedd2 | 3.92E-05 | 2.26E-04 | 2.19E+00 |
| Herc6 | Herc6 | 4.03E-05 | 2.31E-04 | 3.35E+00 |
| Lurap1l | Lurap1l | 4.07E-05 | 2.33E-04 | -2.13E+00 |
| Rpp25 | Rpp25 | 4.10E-05 | 2.34E-04 | -2.14E+00 |
| Nynrin | Nynrin | 4.11E-05 | 2.35E-04 | -2.08E+00 |
| ENSRNOG00000062907 | --- | 4.18E-05 | 2.38E-04 | 3.17E+00 |
| B3gnt8 | B3gnt8 | 4.24E-05 | 2.41E-04 | -2.01E+00 |
| Tspy26 | Tspy26 | 4.25E-05 | 2.42E-04 | -2.10E+00 |
| Kdm6b | Kdm6b | 4.37E-05 | 2.47E-04 | 2.04E+00 |
| Fam83g | Fam83g | 4.41E-05 | 2.49E-04 | 2.54E+00 |
| Prr15 | Prr15 | 4.50E-05 | 2.54E-04 | -3.27E+00 |
| Ccdc170 | Ccdc170 | 4.59E-05 | 2.58E-04 | -2.11E+00 |
| Rbm44 | Rbm44 | 4.67E-05 | 2.62E-04 | 4.06E+00 |
| Sdcbp2 | Sdcbp2 | 4.71E-05 | 2.64E-04 | 3.69E+00 |
| Ccl4 | Ccl4 | 4.74E-05 | 2.65E-04 | 3.46E+00 |
| Tnfaip3 | Tnfaip3 | 4.74E-05 | 2.65E-04 | 3.44E+00 |
| ENSRNOG00000064728 | --- | 4.85E-05 | 2.70E-04 | 3.53E+00 |
| Tgm1 | Tgm1 | 4.86E-05 | 2.71E-04 | 3.02E+00 |
| Shfl | Shfl | 4.88E-05 | 2.71E-04 | 2.25E+00 |
| Ccdc73 | Ccdc73 | 4.94E-05 | 2.74E-04 | 2.25E+00 |
| Slamf1 | Slamf1 | 4.96E-05 | 2.75E-04 | 2.07E+00 |
| Lst1 | Lst1 | 4.97E-05 | 2.76E-04 | 2.96E+00 |
| Fsip1 | Fsip1 | 5.04E-05 | 2.79E-04 | 3.41E+00 |
| Cldn1 | Cldn1 | 5.06E-05 | 2.80E-04 | 2.77E+00 |
| Bdkrb2 | Bdkrb2 | 5.07E-05 | 2.80E-04 | 2.14E+00 |
| Dbh | Dbh | 5.11E-05 | 2.81E-04 | 2.18E+00 |
| Isg15 | Isg15 | 5.15E-05 | 2.83E-04 | 5.31E+00 |
| ENSRNOG00000063182 | --- | 5.15E-05 | 2.83E-04 | 2.25E+00 |
| Pdcd1lg2 | Pdcd1lg2 | 5.22E-05 | 2.86E-04 | -2.08E+00 |
| Tnip1 | Tnip1 | 5.24E-05 | 2.87E-04 | 2.04E+00 |
| Tnfrsf19 | Tnfrsf19 | 5.25E-05 | 2.88E-04 | -2.33E+00 |
| Ephb3 | Ephb3 | 5.34E-05 | 2.92E-04 | -2.09E+00 |
| Phldb3 | Phldb3 | 5.50E-05 | 2.99E-04 | 2.35E+00 |
| ENSRNOG00000037632 | --- | 5.66E-05 | 3.07E-04 | 2.56E+00 |
| Tspan8 | Tspan8 | 5.67E-05 | 3.08E-04 | -2.10E+00 |
| Gch1 | Gch1 | 5.69E-05 | 3.08E-04 | 2.26E+00 |
| Vgll3 | Vgll3 | 5.89E-05 | 3.18E-04 | 2.36E+00 |
| Gbp4 | Gbp4 | 5.93E-05 | 3.19E-04 | 3.81E+00 |
| Arf2 | Arf2 | 5.97E-05 | 3.22E-04 | 2.23E+00 |
| Tnc | Tnc | 6.04E-05 | 3.25E-04 | 3.97E+00 |
| ENSRNOG00000070674 | --- | 6.10E-05 | 3.27E-04 | -2.40E+00 |
| Hba-a2 | Hba-a2 | 6.16E-05 | 3.30E-04 | 2.26E+00 |
| Wars1 | Wars1 | 6.27E-05 | 3.35E-04 | 2.32E+00 |
| Gp1bb | Gp1bb | 6.37E-05 | 3.40E-04 | 3.25E+00 |
| Clec2e | Clec2e | 6.42E-05 | 3.42E-04 | 3.00E+00 |
| Upk3b | Upk3b | 6.70E-05 | 3.55E-04 | -5.91E+00 |
| Epcam | Epcam | 6.76E-05 | 3.58E-04 | 6.90E+00 |
| Themis2 | Themis2 | 6.77E-05 | 3.59E-04 | 2.27E+00 |
| Sh3tc2 | Sh3tc2 | 6.83E-05 | 3.61E-04 | -2.20E+00 |
| Lcn2 | Lcn2 | 7.24E-05 | 3.78E-04 | 9.54E+00 |
| Enho | Enho | 7.23E-05 | 3.78E-04 | -2.49E+00 |
| AABR07028349.1 | AABR07028349.1 | 7.29E-05 | 3.80E-04 | 2.07E+00 |
| Stac2 | Stac2 | 7.30E-05 | 3.80E-04 | 4.49E+00 |
| Hamp | Hamp | 7.44E-05 | 3.87E-04 | 5.02E+00 |
| Id4 | Id4 | 7.67E-05 | 3.98E-04 | 2.11E+00 |
| RGD1305184 | RGD1305184 | 7.71E-05 | 4.00E-04 | 8.71E+00 |
| Emb | Emb | 7.74E-05 | 4.02E-04 | 2.24E+00 |
| ENSRNOG00000068626 | --- | 7.75E-05 | 4.02E-04 | 2.19E+00 |
| Kcnc4 | Kcnc4 | 7.80E-05 | 4.04E-04 | -2.11E+00 |
| Fmo4 | Fmo4 | 7.83E-05 | 4.05E-04 | -2.32E+00 |
| Pcdh12 | Pcdh12 | 7.90E-05 | 4.08E-04 | -2.34E+00 |
| ENSRNOG00000064568 | --- | 7.92E-05 | 4.09E-04 | 3.00E+00 |
| Mcoln3 | Mcoln3 | 8.62E-05 | 4.41E-04 | 2.81E+00 |
| Dcx | Dcx | 9.39E-05 | 4.76E-04 | -2.91E+00 |
| Epha1 | Epha1 | 9.43E-05 | 4.78E-04 | 2.57E+00 |
| Garem2 | Garem2 | 9.60E-05 | 4.85E-04 | 2.37E+00 |
| Ly6g6d | Ly6g6d | 9.72E-05 | 4.90E-04 | 4.21E+00 |
| ENSRNOG00000068410 | --- | 9.80E-05 | 4.94E-04 | -2.05E+00 |
| Il17re | Il17re | 9.83E-05 | 4.95E-04 | -3.01E+00 |
| ENSRNOG00000066845 | --- | 9.85E-05 | 4.96E-04 | -2.04E+00 |
| Tap1 | Tap1 | 9.87E-05 | 4.97E-04 | 2.91E+00 |
| Zfp467 | Zfp467 | 1.06E-04 | 5.30E-04 | -2.05E+00 |
| Samd9 | Samd9 | 1.09E-04 | 5.42E-04 | 3.39E+00 |
| Nr5a2 | Nr5a2 | 1.14E-04 | 5.61E-04 | 3.45E+00 |
| Fgfr4 | Fgfr4 | 1.14E-04 | 5.62E-04 | -2.43E+00 |
| Mpzl2 | Mpzl2 | 1.15E-04 | 5.66E-04 | 3.83E+00 |
| Lhx6 | Lhx6 | 1.15E-04 | 5.66E-04 | -2.64E+00 |
| Pmepa1 | Pmepa1 | 1.16E-04 | 5.70E-04 | 2.12E+00 |
| Coro2b | Coro2b | 1.18E-04 | 5.80E-04 | -2.04E+00 |
| Lpar4 | Lpar4 | 1.19E-04 | 5.81E-04 | -2.07E+00 |
| Clcn1 | Clcn1 | 1.22E-04 | 5.97E-04 | -2.49E+00 |
| ENSRNOG00000070917 | --- | 1.24E-04 | 6.06E-04 | -2.29E+00 |
| ENSRNOG00000064219 | --- | 1.29E-04 | 6.26E-04 | 2.18E+00 |
| Rab11fip1 | Rab11fip1 | 1.30E-04 | 6.27E-04 | 2.05E+00 |
| Acy3 | Acy3 | 1.33E-04 | 6.42E-04 | -2.11E+00 |
| ENSRNOG00000069735 | --- | 1.35E-04 | 6.47E-04 | -2.49E+00 |
| Samd5 | Samd5 | 1.40E-04 | 6.68E-04 | -2.25E+00 |
| Postn | Postn | 1.41E-04 | 6.75E-04 | 2.59E+00 |
| Egr3 | Egr3 | 1.44E-04 | 6.84E-04 | 5.04E+00 |
| Vangl2 | Vangl2 | 1.45E-04 | 6.89E-04 | -2.14E+00 |
| S1pr3 | S1pr3 | 1.46E-04 | 6.93E-04 | 2.12E+00 |
| ENSRNOG00000065534 | --- | 1.47E-04 | 6.95E-04 | -2.04E+00 |
| Casp4 | Casp4 | 1.47E-04 | 6.96E-04 | 2.25E+00 |
| Sostdc1 | Sostdc1 | 1.56E-04 | 7.37E-04 | -2.36E+00 |
| Fmo3 | Fmo3 | 1.57E-04 | 7.39E-04 | 2.14E+00 |
| Wnt9b | Wnt9b | 1.59E-04 | 7.48E-04 | -2.82E+00 |
| Sh2d1b2 | Sh2d1b2 | 1.59E-04 | 7.48E-04 | 2.40E+00 |
| Errfi1 | Errfi1 | 1.60E-04 | 7.50E-04 | 2.84E+00 |
| RGD1564400 | RGD1564400 | 1.60E-04 | 7.53E-04 | 2.85E+00 |
| Fbxo39 | Fbxo39 | 1.63E-04 | 7.64E-04 | 2.88E+00 |
| Atcay | Atcay | 1.73E-04 | 8.05E-04 | -3.01E+00 |
| Mov10 | Mov10 | 1.83E-04 | 8.47E-04 | 2.04E+00 |
| Alox12 | Alox12 | 1.84E-04 | 8.50E-04 | 2.39E+00 |
| ENSRNOG00000063266 | --- | 1.87E-04 | 8.63E-04 | 2.26E+00 |
| Lim2 | Lim2 | 1.88E-04 | 8.65E-04 | -2.41E+00 |
| Ifi47 | Ifi47 | 1.90E-04 | 8.74E-04 | 2.73E+00 |
| ENSRNOG00000069551 | --- | 1.91E-04 | 8.78E-04 | 2.57E+00 |
| ENSRNOG00000066884 | --- | 1.95E-04 | 8.93E-04 | 2.49E+00 |
| Ms4a7 | Ms4a7 | 2.17E-04 | 9.84E-04 | -2.17E+00 |
| ENSRNOG00000011478 | --- | 2.23E-04 | 1.01E-03 | -2.33E+00 |
| AABR07044383.1 | AABR07044383.1 | 2.24E-04 | 1.01E-03 | 2.32E+00 |
| Myl1 | Myl1 | 2.27E-04 | 1.02E-03 | 2.55E+00 |
| Phlda1 | Phlda1 | 2.34E-04 | 1.05E-03 | 2.48E+00 |
| Ntf4 | Ntf4 | 2.34E-04 | 1.05E-03 | 3.02E+00 |
| ENSRNOG00000069167 | --- | 2.37E-04 | 1.06E-03 | -2.30E+00 |
| Socs1 | Socs1 | 2.38E-04 | 1.07E-03 | 4.44E+00 |
| Grem1 | Grem1 | 2.47E-04 | 1.10E-03 | 3.44E+00 |
| Bdnf | Bdnf | 2.64E-04 | 1.17E-03 | 2.08E+00 |
| Fam189a1 | Fam189a1 | 2.70E-04 | 1.19E-03 | -2.21E+00 |
| Cxcl11 | Cxcl11 | 2.76E-04 | 1.21E-03 | 4.37E+00 |
| ENSRNOG00000070699 | --- | 2.83E-04 | 1.24E-03 | 2.07E+00 |
| RGD1559747 | RGD1559747 | 2.85E-04 | 1.25E-03 | -2.06E+00 |
| ENSRNOG00000066846 | --- | 2.86E-04 | 1.25E-03 | 2.91E+00 |
| Mogat2 | Mogat2 | 2.95E-04 | 1.28E-03 | -2.59E+00 |
| P2rx6 | P2rx6 | 3.00E-04 | 1.30E-03 | -2.39E+00 |
| Inhba | Inhba | 3.04E-04 | 1.32E-03 | 3.01E+00 |
| Fam163a | Fam163a | 3.04E-04 | 1.32E-03 | -2.19E+00 |
| Exoc3l2 | Exoc3l2 | 3.05E-04 | 1.32E-03 | -2.65E+00 |
| ENSRNOG00000067235 | --- | 3.06E-04 | 1.33E-03 | -2.07E+00 |
| Cxcr5 | Cxcr5 | 3.06E-04 | 1.33E-03 | 2.25E+00 |
| Duox2 | Duox2 | 3.10E-04 | 1.34E-03 | -2.78E+00 |
| Cdh16 | Cdh16 | 3.14E-04 | 1.36E-03 | 2.44E+00 |
| ENSRNOG00000065214 | --- | 3.36E-04 | 1.44E-03 | 2.13E+00 |
| Lctl | Lctl | 3.45E-04 | 1.47E-03 | 3.00E+00 |
| Neto2 | Neto2 | 3.51E-04 | 1.49E-03 | 2.11E+00 |
| Btnl9 | Btnl9 | 3.61E-04 | 1.53E-03 | -2.82E+00 |
| Irf8 | Irf8 | 3.64E-04 | 1.54E-03 | 2.46E+00 |
| Mettl24 | Mettl24 | 3.72E-04 | 1.57E-03 | -2.29E+00 |
| Crtc2 | Crtc2 | 3.74E-04 | 1.58E-03 | 5.05E+00 |
| Pacrg | Pacrg | 3.75E-04 | 1.58E-03 | 3.13E+00 |
| Cdh20 | Cdh20 | 3.76E-04 | 1.59E-03 | -2.10E+00 |
| Lilrb3a | Lilrb3a | 3.81E-04 | 1.60E-03 | 2.37E+00 |
| ENSRNOG00000066335 | --- | 3.89E-04 | 1.63E-03 | 2.07E+00 |
| ENSRNOG00000067606 | --- | 3.91E-04 | 1.64E-03 | 2.01E+00 |
| ENSRNOG00000069624 | --- | 3.93E-04 | 1.65E-03 | 2.15E+00 |
| Tnfaip2 | Tnfaip2 | 4.00E-04 | 1.68E-03 | 2.67E+00 |
| Slitrk2 | Slitrk2 | 4.28E-04 | 1.78E-03 | -2.27E+00 |
| Slitrk5 | Slitrk5 | 4.38E-04 | 1.81E-03 | -2.35E+00 |
| Tril | Tril | 4.42E-04 | 1.82E-03 | -2.04E+00 |
| ENSRNOG00000066757 | --- | 4.49E-04 | 1.85E-03 | 2.61E+00 |
| Gata1 | Gata1 | 4.54E-04 | 1.87E-03 | 2.50E+00 |
| Neurl3 | Neurl3 | 4.56E-04 | 1.87E-03 | 3.23E+00 |
| Nfil3 | Nfil3 | 4.65E-04 | 1.90E-03 | 2.59E+00 |
| Tnfrsf9 | Tnfrsf9 | 4.71E-04 | 1.93E-03 | 2.61E+00 |
| Rnf133 | Rnf133 | 4.74E-04 | 1.94E-03 | 2.10E+00 |
| Slfn4 | Slfn4 | 5.04E-04 | 2.04E-03 | 2.76E+00 |
| Cldn15 | Cldn15 | 5.29E-04 | 2.13E-03 | -2.12E+00 |
| ENSRNOG00000067090 | --- | 5.30E-04 | 2.14E-03 | 2.07E+00 |
| Nanos1 | Nanos1 | 5.33E-04 | 2.15E-03 | 2.76E+00 |
| ENSRNOG00000030807 | --- | 5.41E-04 | 2.18E-03 | 2.61E+00 |
| ENSRNOG00000021110 | --- | 5.54E-04 | 2.22E-03 | 2.31E+00 |
| Btg2 | Btg2 | 5.82E-04 | 2.31E-03 | 2.29E+00 |
| Slc39a7 | Slc39a7 | 5.96E-04 | 2.36E-03 | 2.64E+00 |
| Ccl21 | Ccl21 | 6.05E-04 | 2.39E-03 | -2.04E+00 |
| ENSRNOG00000003435 | --- | 6.24E-04 | 2.46E-03 | -2.15E+00 |
| ENSRNOG00000067342 | --- | 6.33E-04 | 2.49E-03 | 2.31E+00 |
| ENSRNOG00000065123 | --- | 6.38E-04 | 2.50E-03 | -2.05E+00 |
| ENSRNOG00000025735 | --- | 6.58E-04 | 2.57E-03 | -2.07E+00 |
| Havcr2 | Havcr2 | 6.62E-04 | 2.58E-03 | 2.36E+00 |
| Ucn | Ucn | 6.79E-04 | 2.64E-03 | 2.27E+00 |
| Pacsin1 | Pacsin1 | 7.28E-04 | 2.80E-03 | 2.09E+00 |
| ENSRNOG00000069786 | --- | 7.39E-04 | 2.84E-03 | 2.11E+00 |
| ENSRNOG00000071071 | --- | 7.46E-04 | 2.86E-03 | 3.00E+00 |
| Reg3g | Reg3g | 7.53E-04 | 2.88E-03 | 6.65E+00 |
| Isg20 | Isg20 | 7.57E-04 | 2.89E-03 | 2.15E+00 |
| Il13ra2 | Il13ra2 | 7.95E-04 | 3.02E-03 | 3.33E+00 |
| Pla2g2a | Pla2g2a | 7.99E-04 | 3.03E-03 | 2.04E+00 |
| ENSRNOG00000067370 | --- | 8.13E-04 | 3.07E-03 | -2.01E+00 |
| Rbp7 | Rbp7 | 8.29E-04 | 3.12E-03 | -2.24E+00 |
| Rnf213 | Rnf213 | 8.72E-04 | 3.26E-03 | 2.61E+00 |
| Olr1058 | Olr1058 | 8.78E-04 | 3.28E-03 | -2.17E+00 |
| Trim6 | Trim6 | 8.85E-04 | 3.30E-03 | -2.24E+00 |
| Kcnt1 | Kcnt1 | 8.87E-04 | 3.31E-03 | 2.32E+00 |
| Plcxd2 | Plcxd2 | 8.94E-04 | 3.33E-03 | 2.70E+00 |
| AABR07071000.1 | AABR07071000.1 | 9.30E-04 | 3.45E-03 | 2.85E+00 |
| Trim16 | Trim16 | 9.57E-04 | 3.54E-03 | 2.02E+00 |
| ENSRNOG00000064524 | --- | 9.68E-04 | 3.57E-03 | 2.47E+00 |
| Dnah3 | Dnah3 | 9.80E-04 | 3.61E-03 | 2.41E+00 |
| Slc26a3 | Slc26a3 | 1.01E-03 | 3.70E-03 | -2.11E+00 |
| Arnt2 | Arnt2 | 1.01E-03 | 3.71E-03 | -2.01E+00 |
| ENSRNOG00000064417 | --- | 1.04E-03 | 3.79E-03 | -2.59E+00 |
| Syndig1 | Syndig1 | 1.06E-03 | 3.85E-03 | -2.00E+00 |
| Myb | Myb | 1.08E-03 | 3.92E-03 | 2.84E+00 |
| ENSRNOG00000064821 | --- | 1.08E-03 | 3.92E-03 | 3.33E+00 |
| Ggt1 | Ggt1 | 1.08E-03 | 3.94E-03 | 2.91E+00 |
| Prss12 | Prss12 | 1.09E-03 | 3.94E-03 | -2.20E+00 |
| Slc9b2 | Slc9b2 | 1.10E-03 | 3.98E-03 | 3.17E+00 |
| Akr1b8 | Akr1b8 | 1.10E-03 | 3.99E-03 | 2.82E+00 |
| Dhx58 | Dhx58 | 1.12E-03 | 4.04E-03 | 2.66E+00 |
| Thbs4 | Thbs4 | 1.21E-03 | 4.30E-03 | 2.84E+00 |
| Trh | Trh | 1.22E-03 | 4.35E-03 | 2.83E+00 |
| Oasl | Oasl | 1.23E-03 | 4.36E-03 | 2.92E+00 |
| Add2 | Add2 | 1.23E-03 | 4.38E-03 | 2.11E+00 |
| Ccn4 | Ccn4 | 1.29E-03 | 4.57E-03 | 2.23E+00 |
| Npffr2 | Npffr2 | 1.30E-03 | 4.59E-03 | -2.16E+00 |
| AC120310.1 | AC120310.1 | 1.34E-03 | 4.71E-03 | 2.95E+00 |
| ENSRNOG00000063877 | --- | 1.35E-03 | 4.76E-03 | -2.41E+00 |
| RT1-DOa | RT1-DOa | 1.45E-03 | 5.03E-03 | -2.16E+00 |
| ENSRNOG00000006324 | --- | 1.51E-03 | 5.22E-03 | 2.03E+00 |
| Wnt4 | Wnt4 | 1.52E-03 | 5.26E-03 | 2.08E+00 |
| Parp14 | Parp14 | 1.53E-03 | 5.27E-03 | 2.17E+00 |
| Dusp5 | Dusp5 | 1.54E-03 | 5.31E-03 | 2.71E+00 |
| Ubxn10 | Ubxn10 | 1.59E-03 | 5.45E-03 | -2.59E+00 |
| Tmem229a | Tmem229a | 1.60E-03 | 5.47E-03 | -2.18E+00 |
| Pla2g2c | Pla2g2c | 1.62E-03 | 5.53E-03 | -2.10E+00 |
| Gpr151 | Gpr151 | 1.62E-03 | 5.53E-03 | 2.19E+00 |
| Gjc3 | Gjc3 | 1.62E-03 | 5.54E-03 | -2.03E+00 |
| AABR07060872.1 | AABR07060872.1 | 1.64E-03 | 5.59E-03 | 2.27E+00 |
| Nat8 | Nat8 | 1.71E-03 | 5.79E-03 | -2.01E+00 |
| C4a | C4a | 1.83E-03 | 6.14E-03 | -2.60E+00 |
| Rhpn2 | Rhpn2 | 1.94E-03 | 6.46E-03 | -2.07E+00 |
| Igtp | Igtp | 1.99E-03 | 6.60E-03 | 2.26E+00 |
| Rab42 | Rab42 | 2.00E-03 | 6.62E-03 | -2.04E+00 |
| Klc3 | Klc3 | 2.01E-03 | 6.66E-03 | -2.29E+00 |
| Tspan1 | Tspan1 | 2.12E-03 | 6.97E-03 | 2.56E+00 |
| Cyld-ps1 | Cyld-ps1 | 2.15E-03 | 7.06E-03 | -2.12E+00 |
| Il1a | Il1a | 2.25E-03 | 7.34E-03 | 5.50E+00 |
| Six2 | Six2 | 2.34E-03 | 7.58E-03 | 2.09E+00 |
| ENSRNOG00000063555 | --- | 2.38E-03 | 7.70E-03 | -2.19E+00 |
| ENSRNOG00000059697 | --- | 2.38E-03 | 7.71E-03 | 2.23E+00 |
| Jchain | Jchain | 2.50E-03 | 8.03E-03 | 2.16E+00 |
| ENSRNOG00000067213 | --- | 2.54E-03 | 8.15E-03 | 2.03E+00 |
| ENSRNOG00000062834 | --- | 2.55E-03 | 8.18E-03 | 2.14E+00 |
| Hif3a | Hif3a | 2.61E-03 | 8.36E-03 | 2.80E+00 |
| ENSRNOG00000055496 | --- | 2.69E-03 | 8.54E-03 | 2.84E+00 |
| Slc38a8 | Slc38a8 | 2.76E-03 | 8.71E-03 | 2.95E+00 |
| Pcdh10 | Pcdh10 | 3.20E-03 | 9.89E-03 | 2.02E+00 |
| ENSRNOG00000066766 | --- | 3.23E-03 | 9.95E-03 | 3.70E+00 |
| AY172581.24 | AY172581.24 | 3.23E-03 | 9.96E-03 | 2.02E+00 |
| Reg3b | Reg3b | 3.24E-03 | 9.97E-03 | 3.79E+00 |
| U1 | U1 | 3.27E-03 | 1.00E-02 | 2.07E+00 |
| Zfp365 | Zfp365 | 3.31E-03 | 1.02E-02 | 2.07E+00 |
| Irf1 | Irf1 | 3.32E-03 | 1.02E-02 | 2.37E+00 |
| Pls1 | Pls1 | 3.34E-03 | 1.02E-02 | 2.94E+00 |
| ENSRNOG00000067850 | --- | 3.42E-03 | 1.04E-02 | 2.28E+00 |
| Shox2 | Shox2 | 3.45E-03 | 1.05E-02 | -2.35E+00 |
| Nxpe1 | Nxpe1 | 3.47E-03 | 1.06E-02 | -2.16E+00 |
| Lratd1 | Lratd1 | 3.54E-03 | 1.07E-02 | 2.50E+00 |
| Ccdc85b | Ccdc85b | 3.59E-03 | 1.09E-02 | -2.84E+00 |
| Ccn5 | Ccn5 | 3.60E-03 | 1.09E-02 | -3.04E+00 |
| ENSRNOG00000046469 | --- | 3.66E-03 | 1.10E-02 | -3.13E+00 |
| Tex14 | Tex14 | 3.67E-03 | 1.11E-02 | 3.38E+00 |
| ENSRNOG00000066033 | --- | 3.76E-03 | 1.13E-02 | 2.74E+00 |
| Tcf15 | Tcf15 | 3.76E-03 | 1.13E-02 | -2.01E+00 |
| Lvrn | Lvrn | 3.88E-03 | 1.16E-02 | 2.46E+00 |
| ENSRNOG00000064743 | --- | 3.93E-03 | 1.17E-02 | 3.39E+00 |
| Ccl1 | Ccl1 | 4.01E-03 | 1.19E-02 | 2.57E+00 |
| Kcnj6 | Kcnj6 | 4.06E-03 | 1.21E-02 | 2.01E+00 |
| Oasl2 | Oasl2 | 4.11E-03 | 1.22E-02 | 2.19E+00 |
| ENSRNOG00000069237 | --- | 4.16E-03 | 1.23E-02 | 2.19E+00 |
| Adam12 | Adam12 | 4.23E-03 | 1.25E-02 | 2.12E+00 |
| Fosl1 | Fosl1 | 4.33E-03 | 1.27E-02 | 2.14E+00 |
| Acsbg1 | Acsbg1 | 4.39E-03 | 1.29E-02 | 2.26E+00 |
| Dars2 | Dars2 | 4.47E-03 | 1.31E-02 | -5.44E+00 |
| Phyhipl | Phyhipl | 5.21E-03 | 1.49E-02 | -2.08E+00 |
| Ifit2 | Ifit2 | 5.60E-03 | 1.58E-02 | 2.30E+00 |
| Scd | Scd | 5.88E-03 | 1.65E-02 | -3.17E+00 |
| Lag3 | Lag3 | 5.90E-03 | 1.65E-02 | 2.32E+00 |
| ENSRNOG00000062985 | --- | 5.99E-03 | 1.68E-02 | 2.17E+00 |
| Hbb-b1 | Hbb-b1 | 6.02E-03 | 1.68E-02 | 2.08E+00 |
| Sbspon | Sbspon | 6.08E-03 | 1.70E-02 | -2.10E+00 |
| Kcna2 | Kcna2 | 6.13E-03 | 1.71E-02 | -2.00E+00 |
| Zfp36 | Zfp36 | 6.22E-03 | 1.73E-02 | 2.31E+00 |
| ENSRNOG00000024338 | --- | 6.28E-03 | 1.74E-02 | 2.21E+00 |
| ENSRNOG00000064849 | --- | 6.33E-03 | 1.75E-02 | -2.24E+00 |
| RGD1563285 | RGD1563285 | 6.36E-03 | 1.76E-02 | 2.19E+00 |
| AABR07018244.2 | AABR07018244.2 | 6.43E-03 | 1.78E-02 | 2.37E+00 |
| Nell2 | Nell2 | 6.52E-03 | 1.80E-02 | 2.58E+00 |
| AABR07038029.1 | AABR07038029.1 | 6.56E-03 | 1.80E-02 | 2.01E+00 |
| ENSRNOG00000063744 | --- | 6.90E-03 | 1.88E-02 | 5.52E+00 |
| Egr2 | Egr2 | 6.92E-03 | 1.89E-02 | 2.56E+00 |
| Arhgef16 | Arhgef16 | 6.97E-03 | 1.90E-02 | 2.16E+00 |
| Rag1 | Rag1 | 7.45E-03 | 2.01E-02 | 5.03E+00 |
| ENSRNOG00000022490 | --- | 7.59E-03 | 2.05E-02 | 2.20E+00 |
| Wnt10b | Wnt10b | 7.82E-03 | 2.10E-02 | 2.69E+00 |
| Fpr2 | Fpr2 | 8.02E-03 | 2.15E-02 | 2.61E+00 |
| ENSRNOG00000002635 | --- | 8.05E-03 | 2.15E-02 | 2.10E+00 |
| ENSRNOG00000068276 | --- | 8.16E-03 | 2.17E-02 | 2.06E+00 |
| ENSRNOG00000064013 | --- | 8.24E-03 | 2.19E-02 | -2.27E+00 |
| Mpl | Mpl | 8.39E-03 | 2.22E-02 | 2.24E+00 |
| Cd40 | Cd40 | 8.79E-03 | 2.31E-02 | 2.49E+00 |
| Dmrta1 | Dmrta1 | 9.02E-03 | 2.36E-02 | -2.56E+00 |
| Nos1 | Nos1 | 9.05E-03 | 2.37E-02 | -2.15E+00 |
| AC118772.1 | AC118772.1 | 9.20E-03 | 2.40E-02 | 2.05E+00 |
| Krt8 | Krt8 | 9.74E-03 | 2.53E-02 | 2.99E+00 |
| Smoc1 | Smoc1 | 9.75E-03 | 2.53E-02 | -2.13E+00 |
| Cd274 | Cd274 | 9.78E-03 | 2.53E-02 | 2.05E+00 |
| ENSRNOG00000070827 | --- | 9.79E-03 | 2.53E-02 | 2.18E+00 |
| Krt75 | Krt75 | 9.94E-03 | 2.57E-02 | 2.27E+00 |
| Acot1 | Acot1 | 1.00E-02 | 2.59E-02 | 2.07E+00 |
| ENSRNOG00000030228 | --- | 1.02E-02 | 2.62E-02 | 2.23E+00 |
| Samhd1 | Samhd1 | 1.02E-02 | 2.62E-02 | 2.00E+00 |
| Dlk1 | Dlk1 | 1.02E-02 | 2.63E-02 | 2.09E+00 |
| RT1-CE2 | RT1-CE2 | 1.03E-02 | 2.64E-02 | 2.06E+00 |
| Slamf6 | Slamf6 | 1.04E-02 | 2.66E-02 | 2.12E+00 |
| Gzma | Gzma | 1.04E-02 | 2.67E-02 | 2.21E+00 |
| Alox15 | Alox15 | 1.09E-02 | 2.77E-02 | 2.13E+00 |
| MGC108823 | MGC108823 | 1.17E-02 | 2.94E-02 | 2.37E+00 |
| Pirt | Pirt | 1.18E-02 | 2.98E-02 | -2.26E+00 |
| ENSRNOG00000062812 | --- | 1.26E-02 | 3.14E-02 | -2.17E+00 |
| ENSRNOG00000070853 | --- | 1.30E-02 | 3.23E-02 | 2.31E+00 |
| Tnfrsf13c | Tnfrsf13c | 1.32E-02 | 3.27E-02 | 2.01E+00 |
| Evpl | Evpl | 1.33E-02 | 3.30E-02 | -2.15E+00 |
| Chrdl2 | Chrdl2 | 1.35E-02 | 3.33E-02 | 2.27E+00 |
| ENSRNOG00000070425 | --- | 1.47E-02 | 3.58E-02 | 2.15E+00 |
| Mx1 | Mx1 | 1.50E-02 | 3.64E-02 | 2.69E+00 |
| AABR07069466.1 | AABR07069466.1 | 1.52E-02 | 3.69E-02 | 2.00E+00 |
| Ptgdr2 | Ptgdr2 | 1.61E-02 | 3.86E-02 | 2.48E+00 |
| Pnldc1 | Pnldc1 | 1.75E-02 | 4.13E-02 | 2.11E+00 |
| ENSRNOG00000063195 | --- | 1.76E-02 | 4.16E-02 | 2.38E+00 |
| Oxtr | Oxtr | 1.78E-02 | 4.20E-02 | 2.08E+00 |
| AABR07021871.1 | AABR07021871.1 | 1.79E-02 | 4.21E-02 | 2.19E+00 |
| Rnase1 | Rnase1 | 1.79E-02 | 4.22E-02 | 2.17E+00 |
| Ccdc89 | Ccdc89 | 1.83E-02 | 4.29E-02 | -2.07E+00 |
| Tnnt1 | Tnnt1 | 1.92E-02 | 4.46E-02 | 2.41E+00 |
| Tubb2b | Tubb2b | 1.95E-02 | 4.52E-02 | -2.24E+00 |
