# Supplementary figures and images for: Ischaemic preconditioning regulates cardiac transcriptome via DNA methylation conferring cardio-protection from ischaemic reperfusion injury
Source: Eur Heart J Open. 2025 Oct 10;5(5):oeaf124. doi: 10.1093/ehjopen/oeaf124 (PMC12541389; doi:10.1093/ehjopen/oeaf124)

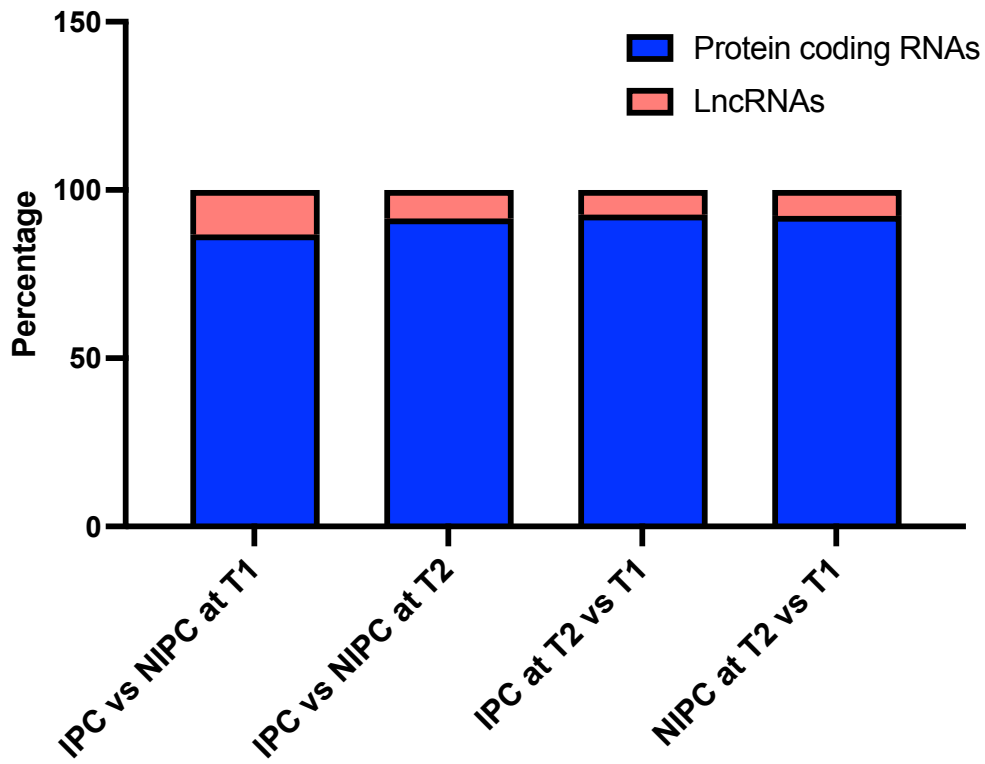

Supp Fig 1

Supplement: oeaf124_Supplementary_Data [file oeaf124_supplementary_data.zip › Supp Fig 1.pdf]

## Description

## Significantly enriched KEGG pathways IPC vs NIPC at T2

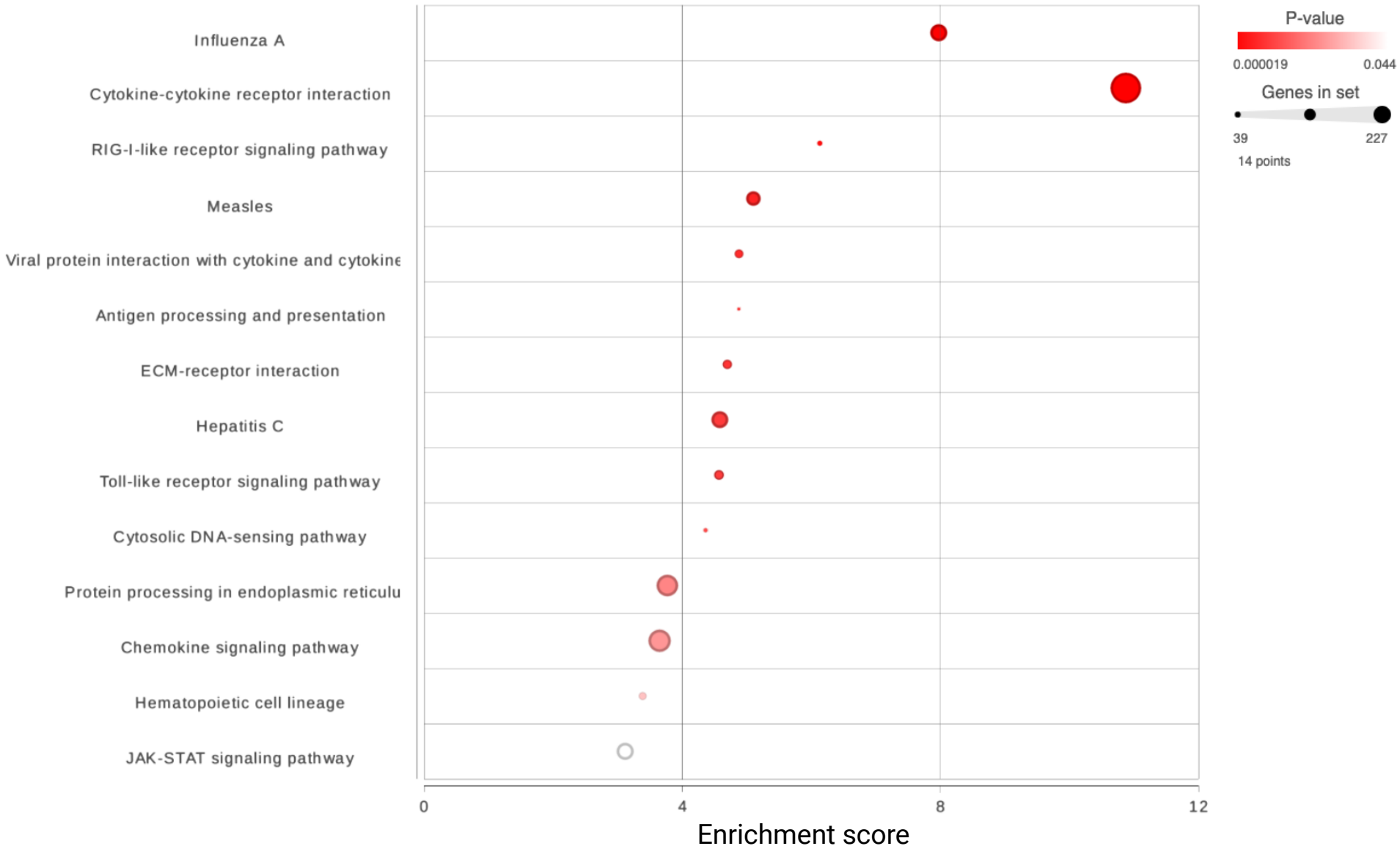

Supplement: oeaf124_Supplementary_Data [file oeaf124_supplementary_data.zip › Supp Fig 3.pdf]

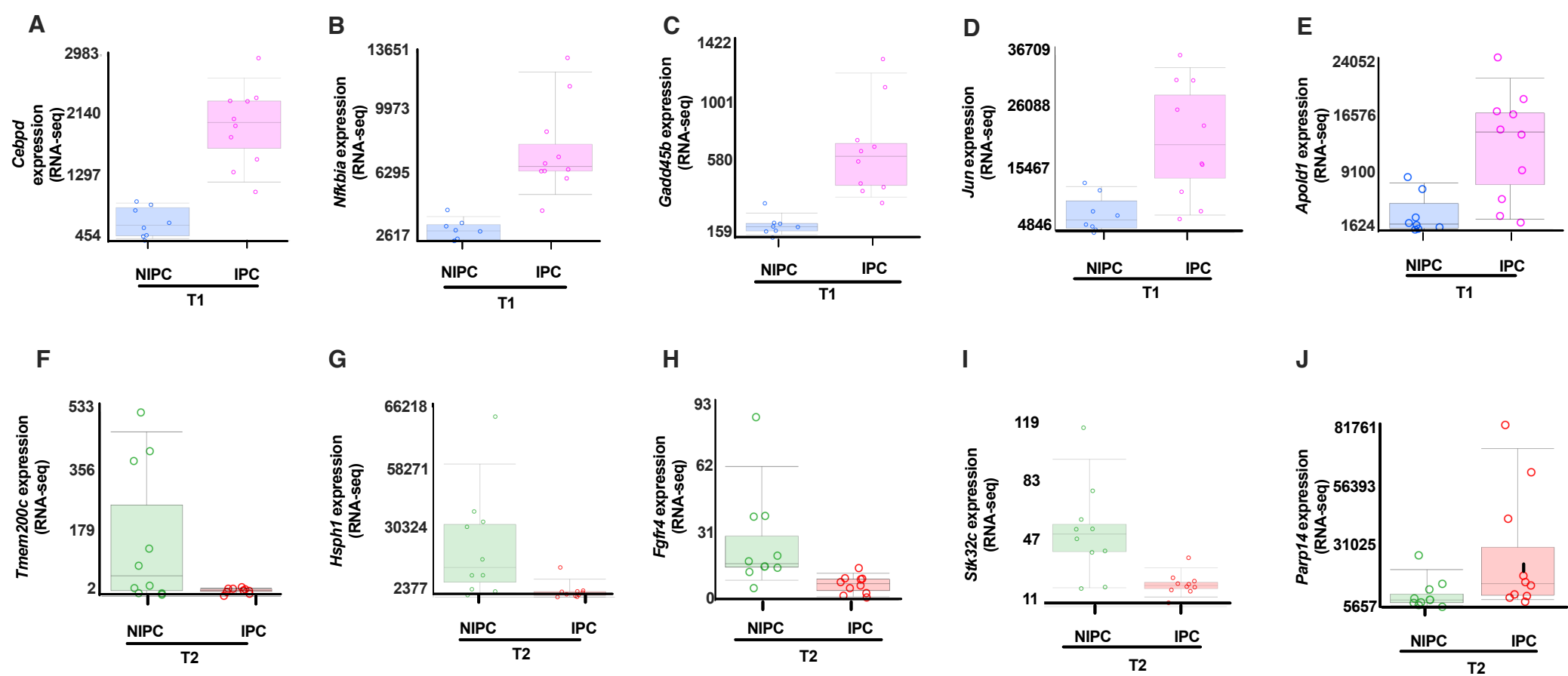

Supp Fig 6

Supplement: oeaf124_Supplementary_Data [file oeaf124_supplementary_data.zip › Supp Fig 6.pdf]
